# Artificial Intelligence to Assist in the Screening Fetal Anomaly Ultrasound Scan (PROMETHEUS): A Randomised Controlled Trial

**DOI:** 10.1056/AIoa2400747

**Published:** 2025-03-27

**Authors:** Thomas G Day, Jacqueline Matthew, Samuel F Budd, Alfonso Farruggia, Lorenzo Venturini, Robert Wright, Babak Jamshidi, Meekai To, Huazen Ling, Jonathon Lai, Min Yi Tan, Matthew Brown, Gavin Guy, Davide Casagrandi, Anastasija Arechvo, Argyro Syngelaki, David Lloyd, Vita Zidere, Trisha Vigneswaran, Owen Miller, Ranjit Akolekar, Surabhi Nanda, Kypros Nicolaides, Bernhard Kainz, John M Simpson, Jo V Hajnal, Reza Razavi

**Affiliations:** 1 School of Biomedical Engineering and Imaging Sciences, King’s College London, UK; 2 Department of Congenital Heart Disease, Evelina London Children’s Healthcare, Guy’s and St Thomas’ NHS Foundation Trust, London, UK; 3 Harris Birthright Centre, King’s College Hospital, London, UK; 4 Department of Fetal Medicine, Chelsea and Westminster Hospital NHS Foundation Trust, London, UK; 5 Department of Fetal Medicine, St Mary’s Hospital, Imperial College Healthcare NHS Trust, London, UK; 6 Department of Fetal Medicine, Medway Maritime Hospital, Medway NHS Foundation Trust, Gillingham, UK; 7 Department of Fetal Medicine, University College London Hospitals NHS Foundation Trust, London, UK; 8 Elizabeth Garrett Anderson Institute for Women’s health, University College London, UK; 9 Fetal Medicine Foundation, London, UK; 10 Department of Fetal Medicine, Guy’s and St Thomas’ NHS Foundation Trust, London, UK; 11 Department of Computing, Imperial College London, London, London, UK; 12 Image Data Exploration and Analysis Lab, Friedrich-Alexander-Universitat Erlangen-Nurnberg, Erlangen, Germany

## Abstract

**Background:**

Artificial intelligence (AI) has shown potential in improving the performance of screening fetal anomaly ultrasound scans. We aimed to assess the effect of AI on fetal ultrasound scanning in terms of diagnostic performance, biometry, scan duration, and sonographer cognitive load.

**Methods:**

This was a randomised, single centre, open label trial in a large teaching hospital. Pregnant participants with fetal congenital heart disease (CHD) and participants with healthy fetuses were recruited and scanned with both methods. Sonographers were recruited from regional hospitals and were randomised to scan with either the AI tool or the standard method, blinded to the fetal CHD status. For the AI-assisted scans, the AI models identified and saved 13 standard image planes and measured four biometrics (but did not automate CHD diagnosis). The primary outcome was the diagnostic performance of the scan; secondary outcomes were scan duration and sonographer cognitive load, as well as biometry performance.

**Results:**

78 pregnant participants (26 with fetal CHD) and 58 sonographers were recruited. The sensitivity and specificity of the AI-assisted scan in detecting fetal malformation was 88.9% and 98.0% respectively, with the standard scan achieving 81.5% and 92.2% (differences in proportions 7.4% for sensitivity (97.5% CI -15.9 – 30.7 %) and 5.9% for specificity (97.5% CI -3.8 – 15.5%)). AI-assisted scans were shorter than standard scans (median 11.4 min vs. 19.7 min, 95% CI for mean difference 7.4 - 11.1 min). Sonographer cognitive load was lower in the AI-assisted group (median NASA TLX score 35.2 vs. 46.5, 95% CI for mean difference 4.6 - 15.4). For all biometrics, the AI repeatability and reproducibility was superior to manual measurements. No adverse events were noted during the trial.

**Conclusion:**

AI assistance in the routine fetal anomaly ultrasound scan results in significant time savings, and a reduction in sonographer cognitive load, without a reduction in diagnostic performance.

## Background

Congenital malformations are the most common causes of infant mortality in high-income countries such as the United Kingdom (UK) and the United States (US), and are becoming increasingly important worldwide as other causes of child death become less common.^1^ Antenatal diagnosis has been shown to reduce postnatal mortality and morbidity for some lesions, may lead to therapeutic intervention in selected cases, and allows parents to make an informed decision about whether they wish the pregnancy to continue.^2,3^ The mainstay of antenatal diagnosis is the fetal anomaly screening ultrasound scan. In the UK, the Fetal Anomaly Screening Programme (FASP) stipulates an offer of this scan between 18^+0^ and 20^+6^ weeks gestation, with the aim of detecting 11 specific fetal conditions.^4^

Fetal anomaly scans have very high rates of uptake but universal detection of major fetal malformations has not been achieved. In the UK only 50.4% of infants requiring congenital heart disease (CHD) surgery have received an antenatal diagnosis and there is wide regional variation across the country.^5^ Artificial intelligence (AI) has been proposed to improve medical task performance, including the fetal anomaly ultrasound scan.^6^ Previous studies have described the development of AI models to automate aspects of the scan such as plane detection and fetal biometry^7–11^, including a pilot study by our group examining prospective real-time use with normal fetuses.^6^

Despite recent interest in AI across medicine, including obstetrics, high-quality prospective clinical trials remain scarce.^12^ While many studies report good model performance using retrospective ultrasound data, no prospective randomised trial has examined the real-world effect of AI on fetal anomaly scans, including abnormal fetuses.

We have created a clinical tool that combines AI models for real-time plane detection, image saving, and biometric measurement with live sonographer feedback. By automating these tasks, the tool streamlines workflow, reducing interruptions and potentially improving fetal malformation detection. This randomised controlled trial examines the effects of this tool in a population including fetuses with known major structural malformations, involving sonographers from a variety of professional backgrounds. We selected CHD as the focus of the study due to its prevalence, high infant mortality, and frequent missed diagnoses.^13,14^ Trial outcome measures were sensitivity and specificity for CHD detection, scan duration, sonographer cognitive load, the quality of saved images, and repeatability and reproducibility of automated measurements. To assess diagnostic performance within a feasible sample size, a novel trial design enriched with CHD-affected fetuses was employed. Variation between participants (both pregnant participants and sonographers) was controlled for by having all pregnant participants undergo both AI-assisted and standard scans, with sonographers randomly assigned to each method.

## Methods

### Study design and participants

The PROMETHEUS trial (Prospective tRial of Machine lEarning To Help fEtal Ultrasound Scanning) was a single-centre randomised controlled open-label trial of AI-assisted vs. standard unassisted fetal anomaly ultrasound scans. The study was designed so that on a given study day three pregnant participants (two with a fetus with a normal heart, one with a fetus with CHD) were invited to attend the study site (although on some days fewer than three pregnant participants actually attended), along with two sonographers, who were randomised to perform scans either with or without AI assistance. Each pregnant participant was scanned twice sequentially, once using each method. The scans were research investigations, not intended to perform a clinical purpose, and performed in addition to standard clinical investigations, thus standard care was not affected by participation in the trial. The trial was conducted in the Clinical Research Facility of a large urban teaching hospital in the UK. The study protocol was prospectively registered with ISRCTN, number 65824874. The study was conducted in line with Good Clinical Practice, and all staff members involved in the running of the trial were fully trained in these guidelines.

Pregnant participants were recruited from a tertiary centre of fetal cardiology, either following a diagnosis of fetal congenital heart disease (“affected group”), or in whom the fetus had been confirmed to have a normal heart structure after detailed fetal echocardiography (“unaffected group”). The unaffected group had been offered detailed fetal cardiac screening because of a risk factor for CHD, such as family history, maternal diabetes, or drug exposure. Inclusion criteria were either diagnosis of fetal CHD between 12^+0^ and 27^+6^ weeks gestation (for the affected group) or confirmation of normal fetal cardiac anatomy between 18^+0^ and 27^+6^ weeks gestation (for the unaffected group), with at least one week between CHD diagnosis and recruitment, if present. Exclusion criteria for pregnant participants were: any plan for termination of pregnancy; any known fetal extracardiac structural abnormality at the time of recruitment; any known fetal genetic abnormality; multiple pregnancy; refusal of consent; insufficient English language skills to provide informed consent; or age under 18. Potential participants were contacted by telephone after approval from the clinical specialist nursing team caring for the patient. The research anomaly scans were performed between 18^+0^ - 27^+6^ weeks’ gestation. Each pregnant participant attended for a single research session. Participants were enrolled on the day of the scan session, with written consent obtained that day.

Sonography professionals were recruited from units within South East England via email invitation to the sonographer lead at each department, with a request for them to cascade to their staff. Advertisements were also placed in electronic newsletters of professional groups (British Medical Ultrasound Society and The Society of Radiographers). The inclusion criterion was the regular independent performance of fetal anomaly screening ultrasound scans as part of their clinical work. Exclusion criteria were any previous involvement in our research projects or refusal of consent. Written consent was obtained from both pregnant participants and sonographers. They could be from any professional background (e.g., radiography, midwifery, nursing, or medical), and all were termed ‘sonographers’ for the purposes of this study.

Ethics approval was granted by the London Dulwich Research Ethics Committee, reference 22/LO/0163.

### Randomisation and masking

Randomisation was performed at a sonographer level on the day of the study session. The pair of sonographers attending for that study session were randomised such that one performed the scan with AI assistance, and the other performed a standard manual scan. An online tool was used for the randomisation (randomizer.org). This was used to generate a sequence of two unique numbers between the range 1-2, with 1 corresponding to the AI-assisted scan and 2 corresponding to the manual scan. The first number in the sequence was used to assign the scan type to the sonographer with the surname that was first alphabetically, and the second number used for the other sonographer. The sonographers were blinded to the clinical status of the pregnant participants (i.e., healthy or CHD), and CHD as the focus of the study.

### Procedures

The AI models used in this study performed two tasks: 1) the detection and labelling of standard image planes from a stream of ultrasound video, and 2) automatic fetal biometry. The models were developed using a prospectively acquired dataset of 7,309 complete videos of routine anomaly ultrasound scans performed in a single institution. The training and testing of the AI models are described in Supplementary Information 1, and described more fully in Venturini *et al*. and Baumgartner *et al*.^7,15^ The standard planes and biometrics used in the study are shown in Table 1, based on the UK Fetal Anomaly Screening Programme (FASP).^4^

**Figure 1:**
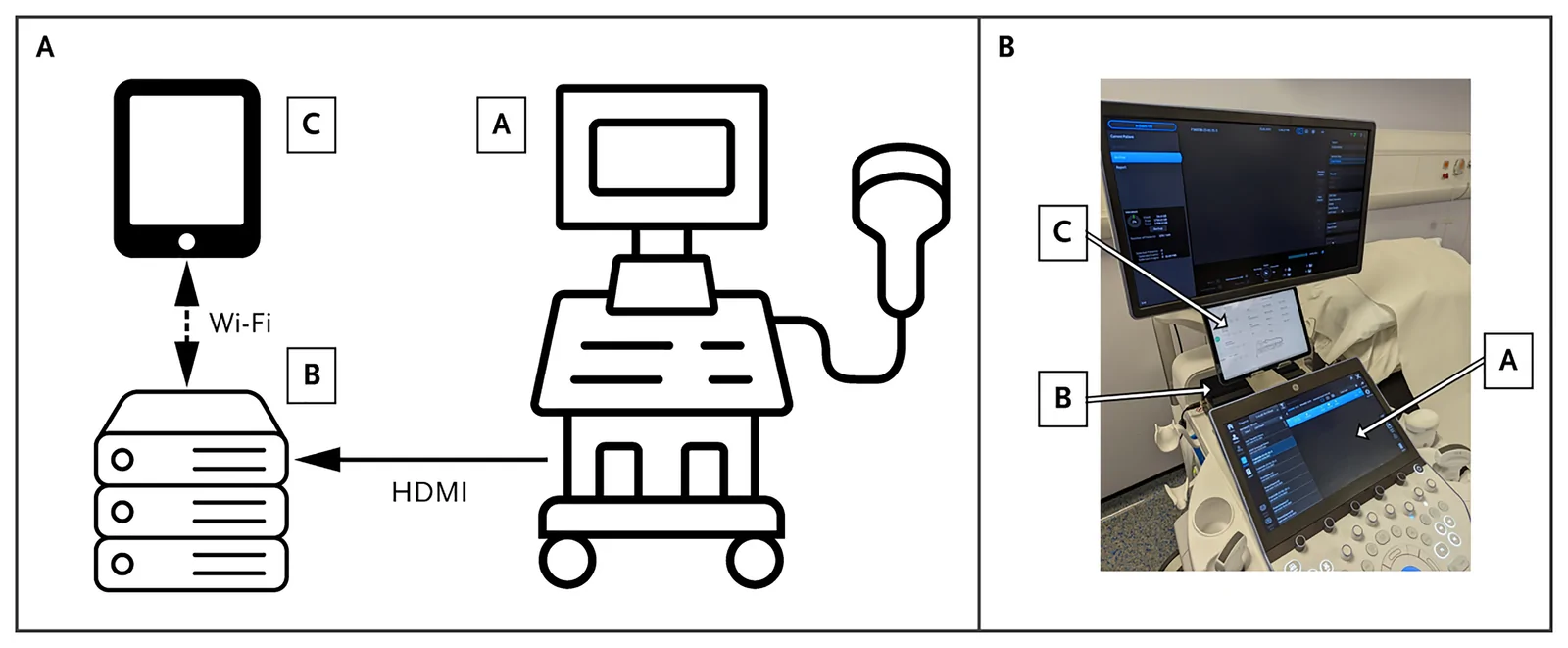
technical integration of artificial intelligence assistance tool. 1: schematic diagram of study setup. 2: photograph of study setup. A: standard ultrasound machine; B: computer mounted on ultrasound machine, receiving video stream of ultrasound scan via HDMI cable; C: tablet displaying outputs of AI models; HDMI: high-definition multimedia interface; solid black arrow: physical cable connection; dashed black arrow: connection via internet.

The ultrasound machine used was a GE Voluson Expert 22. Our clinical AI tool consisted of a computer (Boxer-8641AI, Aaeon Technology Inc., Taipei, Taiwan) mounted on the ultrasound machine, receiving the stream of ultrasound video via a high-definition multimedia interface (HDMI) connection. The individual images within the video stream were analysed in real time by AI models as described in Supplementary Information 1 and 2, with outputs immediately displayed to the sonographer via a tablet (iPad Air 5^th^ Generation, Apple Inc. Cupertino, US). The tablet was connected to the computer via a Wi-Fi connection. A schematic diagram and photograph of the technical setup is shown in Figure 1.

**Figure 2:**
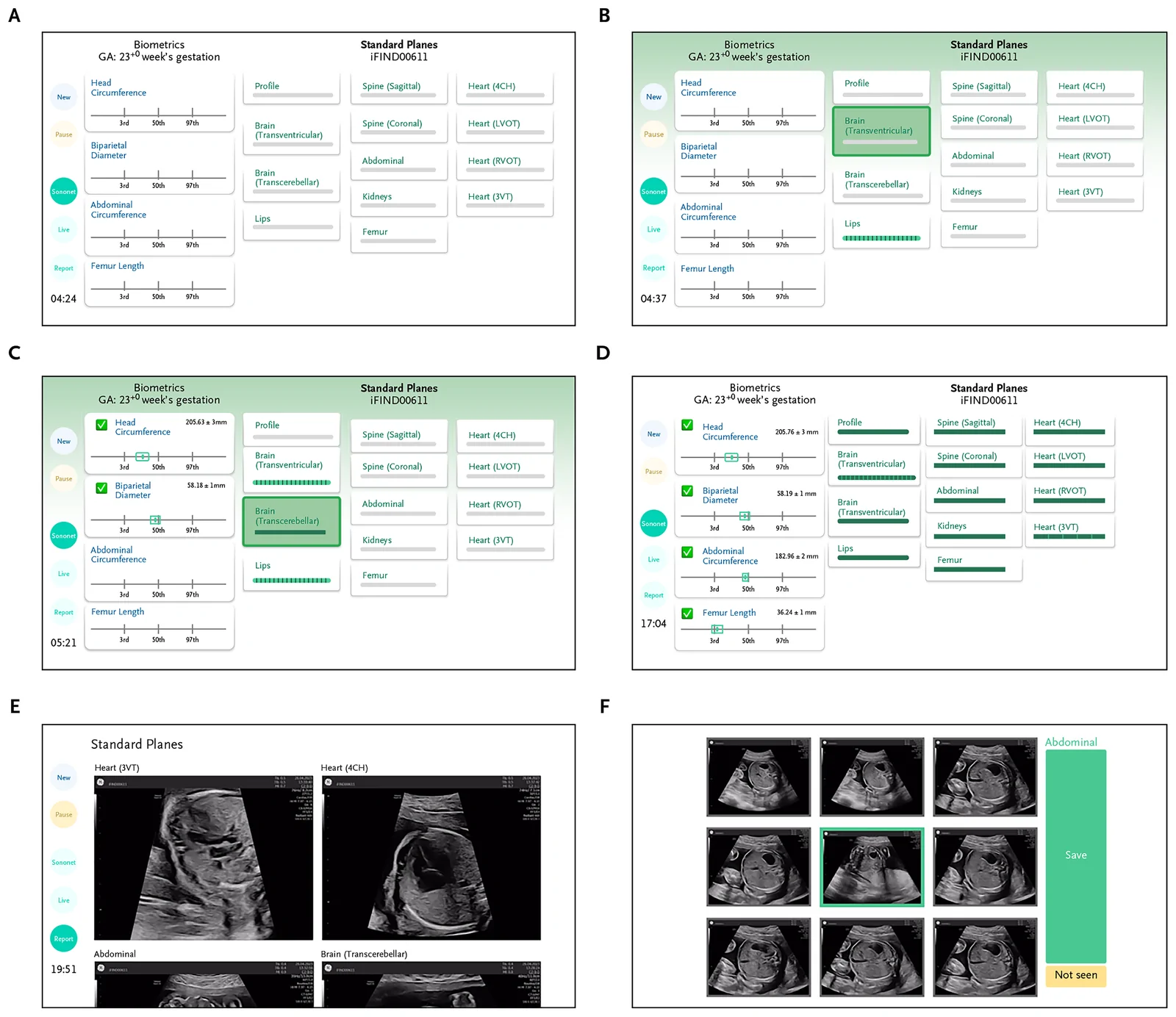
example screenshots from the tablet at different time points of the examination. A: the start of the scan, before any images have been saved; B: during the scan, the device has detected that the brain transventricular view is being imaged, indicated by an overall green colour of the upper screen, and a green highlight of that view panel. The progress bar below the lips view indicates that some images have also been saved; C: during the scan, the brain transcerebellar view is being imaged. Current estimates for the head circumference and biparietal diameter are displayed on the left of the screen, on a scale corresponding to normal range for gestation; D: at the end of the scan all biometrics are displayed and all planes have some saved images; E: the image review stage, where one candidate image per plane is displayed to the sonographer; F: if a plane is selected a further 8 candidate images are displayed to the sonographer to choose from.

Sonographers underwent a 15 minute 1:1 training session on the day of the study with an investigator and a written guide and video, shown in Supplementary Information 3. They were asked to follow a study-specific scan protocol, shown in Supplementary Information 4. Sonographers performing the manual scan were asked to save a single image for each of the 13 standard planes. They were asked to measure each of the four biometric parameters three times and select the best measurement for their report (as per published guidance).^16^ For the sonographers performing the AI-assisted scan, saving of image planes and measurement of biometrics was performed automatically by the AI tool. Feedback on these processes was shown to the AI-assisted sonographer via the tablet, with example screenshots from the tablet shown in Figure 2. During the scan, the current best estimate for each biometric was displayed in real time to the sonographer on a scale indicating the normal values for the given gestation, along with a calculated error bound. Similarly, for each standard plane, a labelled progress bar indicated how many images had been saved. For the AI-assisted scan, after completion the sonographers were shown a candidate image for each of the standard planes on the tablet. They could either accept this image or choose from a further eight images for each plane to be selected as their final best image (the “image review” stage). The sonographer remained responsible for assessing the imaging data from the scan, in order to diagnose fetal malformation. The AI tools did not perform any automated diagnosis of fetal malformation.

After each scan, sonographers completed a written report on a standard laptop using a web-based interface. They were asked to choose a final outcome of: a) standard follow up (i.e., usual antenatal care), b) a repeat scan in a screening department (e.g., due to limited visibility of an area due to fetal position), or c) referral to specialist services (e.g., because CHD had been identified). They were asked to make this decision based on the images that they had seen during the scan or image review stage. They also completed survey instruments to measure cognitive load using both the NASA-TLX scale and Paas scale.^17,18^ The NASA-TLX scale was used unweighted as previously described, and is a multidimensional instrument with six subscales (mental, physical, and temporal demands, and frustration, effort, and performance), designed to capture different aspects of cognitive load, each recorded using a visual analogue scale and then summed.^19^ The Paas scale is a 9-point Likert scale response to the question “please rate your mental effort required to perform the scan.”

After the end of the trial, the quality of each saved image was assessed by fetal medicine experts, blinded to the method used to acquire each image, with at least two experts scoring each image. Experts agreed to this quality scoring scheme by consensus, with further details in Supplementary Information 5. This resulted in two metrics for each image: a binary outcome indicating if the image was deemed ‘clinically acceptable’ or not, and a further continuous outcome indicating overall image quality, normalised to a scale from 0-1. Further AI models that could automatically discriminate the quality of saved images became available after the trial had commenced. To examine the effectiveness of these, all saved images from the AI scans were passed through these models, with the top nine images displayed to a research sonographer, as in the live trial. The best quality image from these nine was selected by an experienced research sonographer and assessed using the same quality scoring scheme as the initially selected images. This resulted in a single image being chosen and graded per plane per participant for each of the three methods (manual acquisition, AI acquisition, and AI acquisition with retrospective use of quality models).

### Outcomes

The primary outcome measures were the sensitivity and specificity of the two methods in detecting CHD. A scan was defined as positive for CHD if at least one of the cardiac views was described as abnormal or not seen in the written report and the final outcome of the scan was a referral to specialist services. All other scans were defined as negative for CHD. This was compared to the ground truth to classify all scans as true positive, false positive, true negative, or false negative for fetal CHD. If an unaffected fetus was unexpectedly identified as being suspected of having CHD an urgent repeat specialist fetal echocardiogram was performed on the same day to define whether this was a true or false positive finding. Secondary outcome measures were the time taken to complete the scan and report (measured by a clinical research fellow or research assistant during the study session with a standard stopwatch), the cognitive load of the sonographers (measured by the administration of survey instruments by a clinical research fellow to sonographers after each scan), and the repeatability and reproducibility of fetal biometrics. All pregnant participants were followed up after delivery to confirm that the antenatal diagnoses were correct. Adverse events of all severities were collected by a clinical research fellow during the scan sessions and at the follow up contact.

### Statistical analysis

The sample size gave an 80% power to demonstrate non-inferiority of CHD detection sensitivity with a target of 80% and margin of 25%. The primary outcome measures of sensitivity and specificity of the ultrasound scans were compared by calculating the 97.5% confidence intervals (corrected from 95% intervals due to multiple testing, using the Bonferroni method). Confidence intervals for proportions were calculated using the exact Clopper-Pearson method, and for differences in proportions using the Wald method. The secondary outcome measures of scan duration and sonographer cognitive load were compared by calculating 95% confidence intervals for the mean differences between groups. Since these were secondary endpoints, hypothesis testing was not performed as per journal guidelines. Statistical analysis was performed using SPSS version 29.0.0.0 (IBM Corporation, Armonk, US). The statistical analysis plan was agreed to before the commencement of data collection.

**Figure 3:**
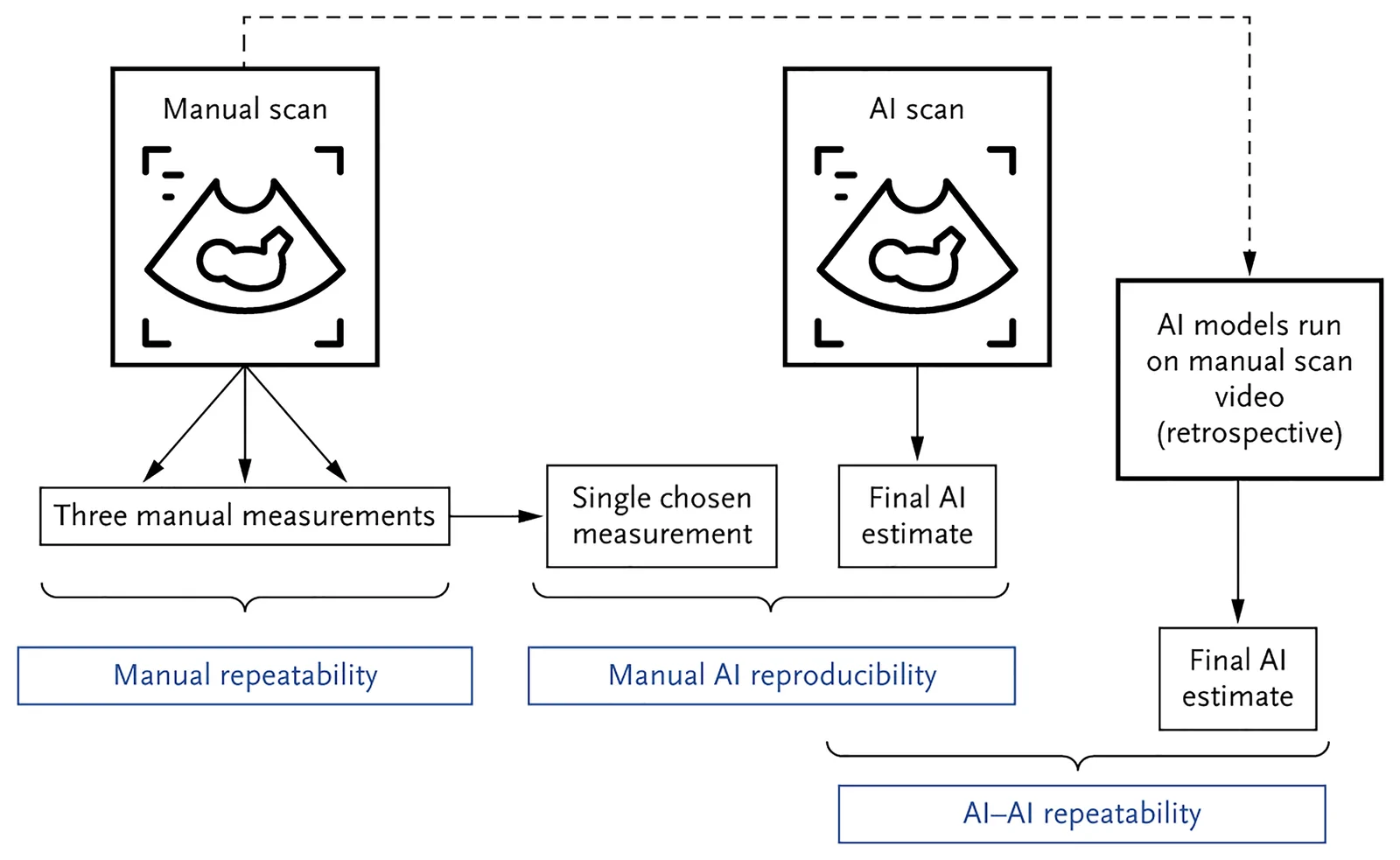
schematic diagram describing comparison of the biometric measurements.

Manual and AI biometrics were compared using Bland-Altman plots. The three measurements per biometric recorded during the manual scan were used to calculate the repeatability of the manual method (since the mean of distance between the maximum and minimum of n observations for a uniform distribution over interval (a b) is equal to ((n-1)/(n+1))*(b-a), by using the coefficient 2/3 a balance was made between these three-measurement criteria, and the other comparison which had only two measurements). The chosen ‘best’ manual measurement was compared with the final estimate from the AI-assisted scan to compare reproducibility between the two methods. The mean difference between the two methods was also subtracted from the AI measurement, so that the random error could be visualised (i.e. removing any systematic bias). Manual human reproducibility (interobserver variability) was not measured in this trial design, but this has been published previously for three of the four biometrics.^20^ Finally, the video recorded during the manual scan was analysed by the AI model retrospectively to obtain a second AI biometric measurement on the same patient on a sequential scan, so that AI-AI repeatability could be assessed. Figure 3 shows a diagram of how these measurements were compared.

To assess for possible confounders, logistic and quantile regression were performed taking into account differences in the sonographers randomised to each scan method, with primary and secondary outcomes as outputs of these models.

### Reporting

This trial has been reported according to the CONSORT-AI extension (supplementary file).^21^

## Results

**Figure 4:**
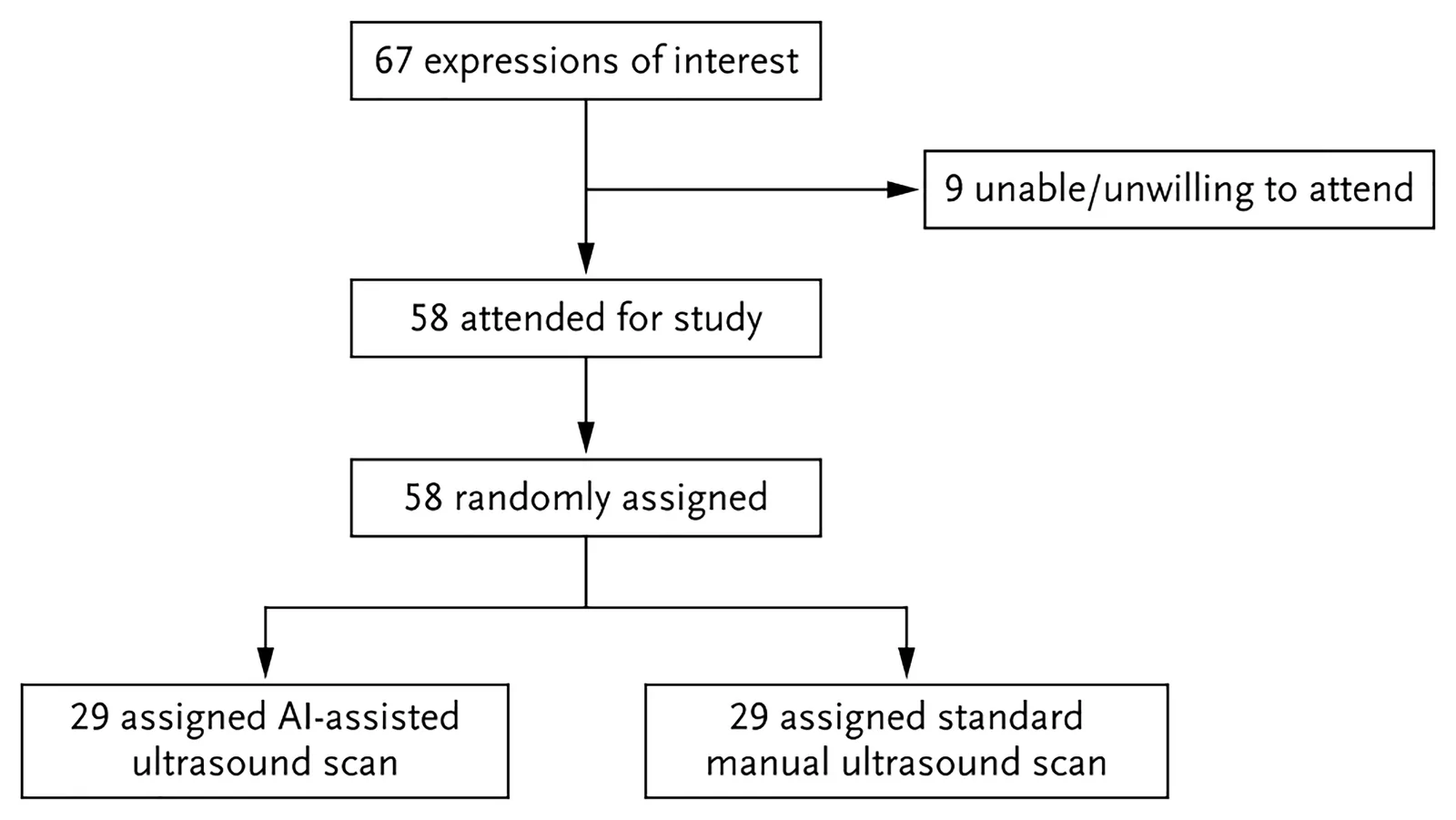
recruitment flowchart for sonographer participants. All sonographers performed the scan method they had been allocated via randomisation.

**Figure 5:**
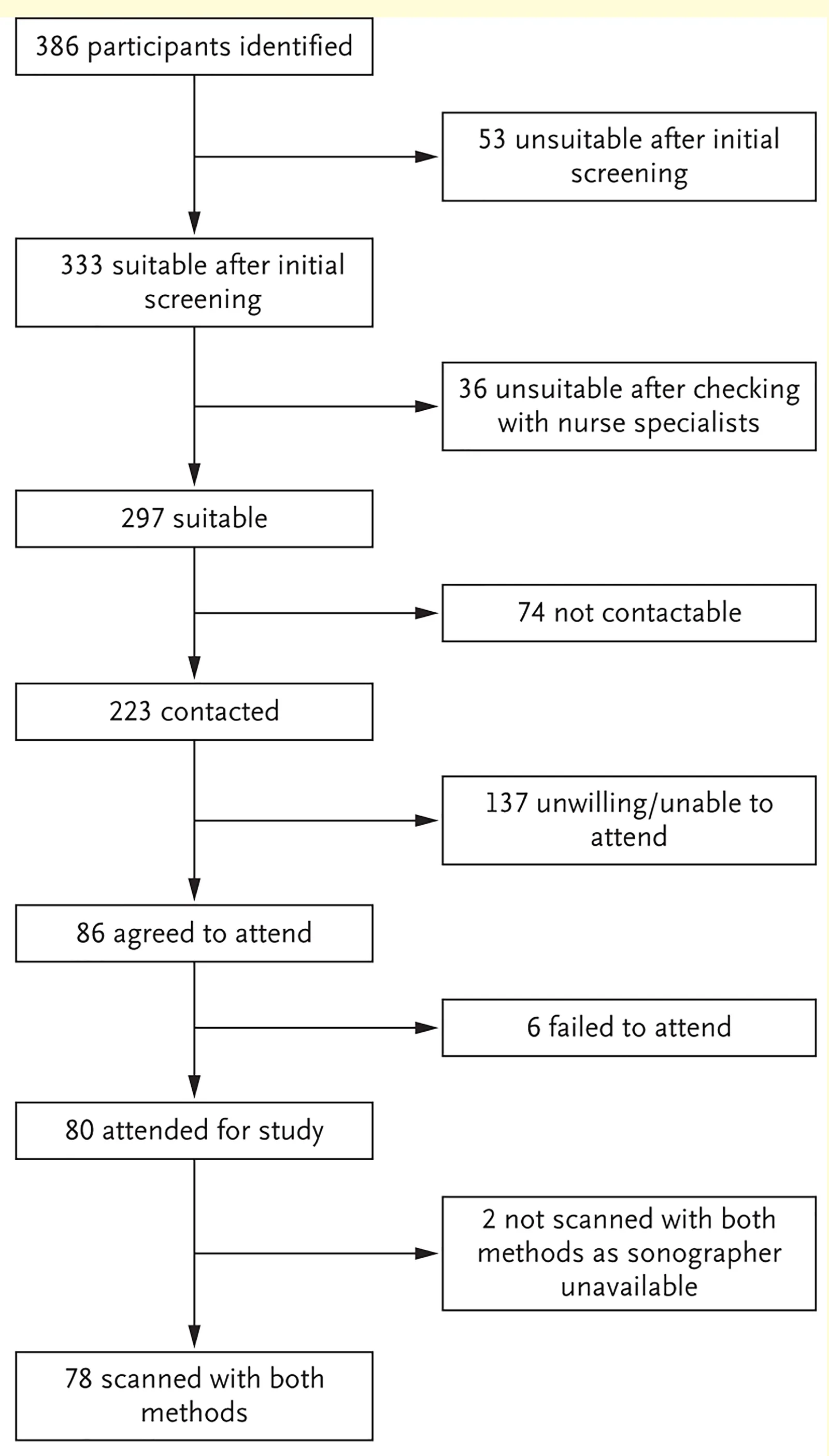
recruitment flowchart for pregnant participants.

Figure 4 shows recruitment figures for sonographers, with 58 recruited over the period 05/05/2022 – 17/07/23. 29 sonographers were randomised into each scanning method. Baseline characteristics after randomisation are shown in Table 2. Figure 5 shows recruitment figures for pregnant participants, with 78 recruited over the period 17/11/22 – 01/08/23 and included in the final analyses. Baseline characteristics are shown in Table 3, with details of the CHD lesions in cases in Table 4. The study sessions ran from 15/11/22 – 08/08/23.

Although 156 paired scans were performed, data were not available from every scan for every outcome measure because of prototype software or hardware failures during the study procedures. This is described in Supplementary Information 6.

The primary outcome measure for the trial was the diagnostic performance of the scan in detecting fetal CHD. The AI-assisted scan was non-inferior to the manual scan both in terms of sensitivity and specificity, with the lower bound of the 97.5% CI (used rather than 95% CI to correct for multiple testing) crossing zero but not crossing the pre-specified margin of 25%, as shown in Table 5.

Postnatal outcome was available for 73 out of 78 fetuses (97.3%), with five not contactable after birth (all in the unaffected group). All fetuses in the CHD group had a postnatal diagnosis that was concordant with their antenatal cardiac diagnosis. Six fetuses in the group unaffected by CHD had a postnatal echocardiogram due to either a heart murmur on routine postnatal examination, or family history of CHD. Three of these found minor abnormalities that would not be considered detectable on routine antenatal screening (one small secundum atrial septal defect, one very mild pulmonary valve stenosis not requiring treatment, and one subtle hypertrophy of the left ventricle not requiring treatment), so they remained in the unaffected group for the purposes of analysis. The other three were entirely normal.

Although known fetal extracardiac abnormalities at time of recruitment was an exclusion criterion, two pregnant participants were included with extracardiac abnormalities as they were identified after recruitment but prior to the study session (one unilateral hydronephrosis, treated with conservatively after birth, and one talipes, treated surgically after birth), one of these was also affected by CHD. Both were identified by both scanning methods. In addition, there were four suspected abnormalities identified during the study scans that were found to be not present on subsequent expert review and after birth (two suspected talipes, one suspected echogenic bowel, and one suspected cleft lip), all suspected during the AI-assisted scan only. Three of these four had coexisting CHD. Table 6 shows an alternative analysis in which all fetal structural malformations (CHD plus extracardiac anomalies) are considered as the affected group. When analysed in this way, the AI-assisted scanning method remains non-inferior to the manual scanning method in terms of diagnostic performance

**Figure 6:**
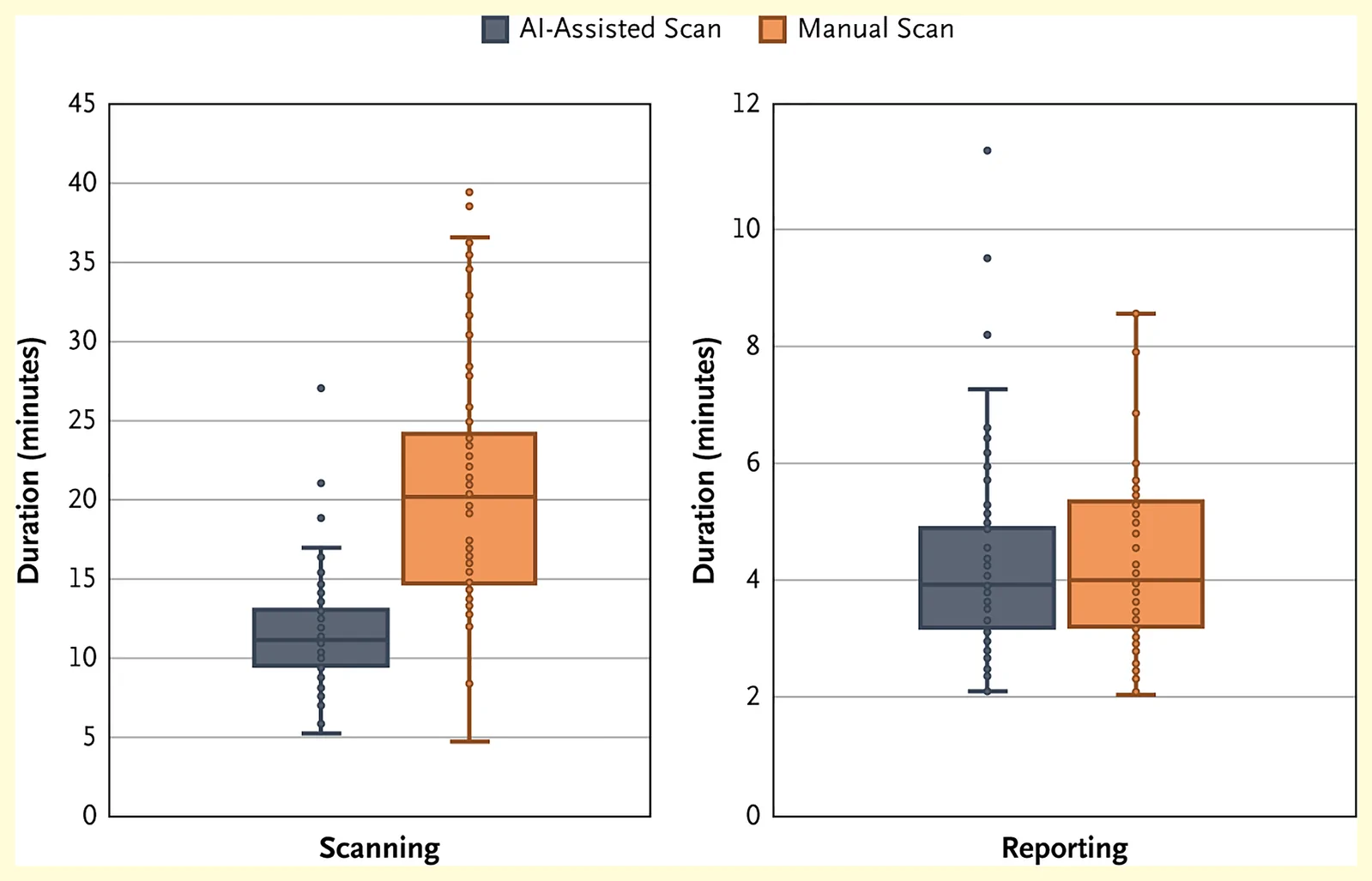
duration of scanning and reporting by both methods.

Results for scan and reporting duration are shown in Table 7 and Figure 6. The tablet-based image review stage (unique to the AI-assisted scan) was included in reporting time. The median scan duration was shorter for the AI-assisted scan, with a mean difference in scanning duration of 9.3 min (95% CI 7.4 - 11.1 min), shown in Table 7. There was no difference in reporting time between the two groups, with a 95% confidence interval that crossed zero.

**Figure 7:**
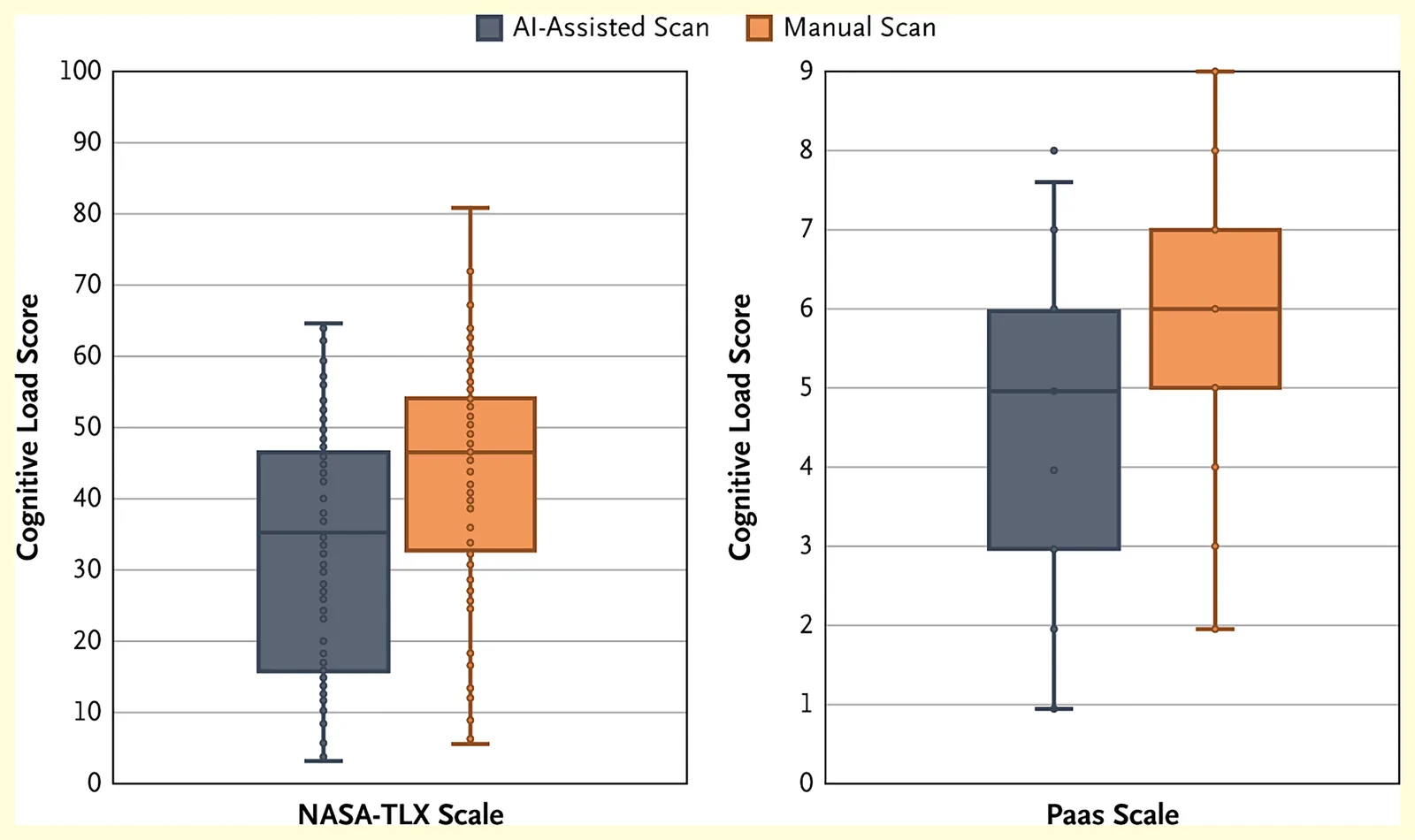
cognitive load of the sonographers compared between the two scanning methods.

The cognitive load of the sonographers was compared between the two groups using both the unweighted NASA-TLX scale and the Paas scale. By both metrics, the sonographers in the AI-assisted scan group reported lower cognitive load than those in the manual scan group (NASA-TLX median score 35.3 vs. 46.5 respectively, mean difference 10.0 (95% CI 4.6-15.4), Paas scale 5 vs. 6, mean difference 0.95 (95% CI 0.3-1.6)). This is shown in Figure 7. For both scan duration analysis and cognitive load, we have not adjusted these secondary endpoints for multiple testing.

**Figure 8:**
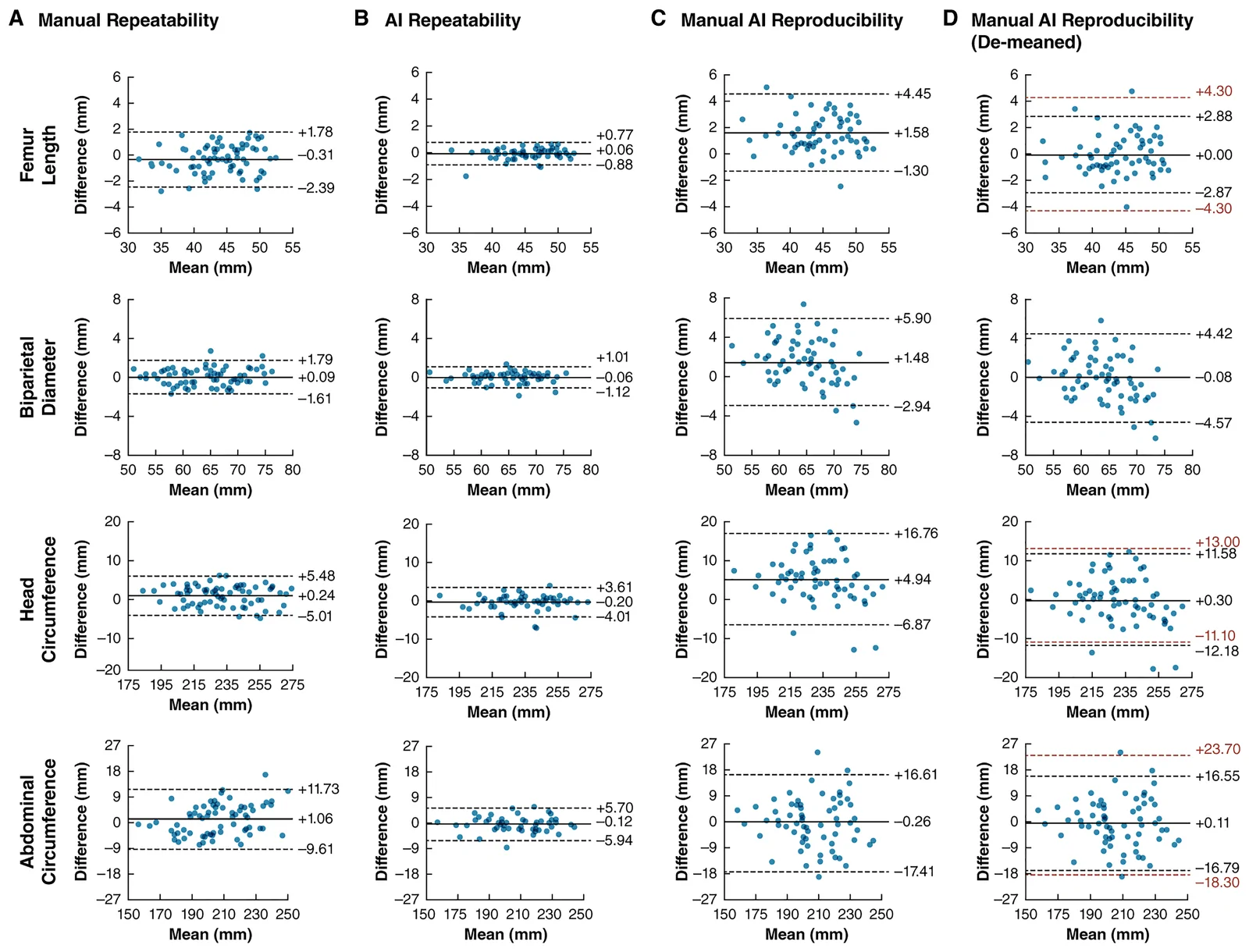
Bland-Altman plots for the four biometric measurements. Solid line: mean difference; dashed lines: the upper and lower 95% limits of agreement; A: the manual measurement repeatability, based on the three measurements taken during the manual scan; B: the repeatability of the AI measurement; C: reproducibility between the final chosen manual measurement with the measurement from the AI-assisted scan; D: as C but with the mean AI-manual difference subtracted from the AI value, to show only random and not systematic error, meaning that human interobserver variability (taken from a previous publication by Sarris et al^20^ can be directly compared (shown in red).

Figure 8 shows the results for the biometric measurements. This analysis was post-hoc. The repeatability of the AI measurements (column B) was superior to that of the manual method (column A), as shown by the tighter 95% limits of agreement for repeated measures for all four biometrics. We identified a systematic bias in the reproducibility between AI and manual measurements (i.e., a systematic difference between the two methods, in the absence of a measurable ground truth), from -0.26 (abdominal circumference) to +4.94 mm (head circumference), shown in column C. We did not measure reproducibility between two different human observers, but this has been measured previously^20^ and is shown in red in column D (in this column the systematic bias has been removed by subtracting the mean difference, to allow direct comparison of the random error between the two groups). The random error seen when comparing AI to manual measurements was less than the random error seen between two humans. However, this estimate of human interobserver variability was made using different data and may not be directly comparable to our findings.

Logistic and quantile regression models were performed to assess for possible confounders. We did not find any significant association between any of the sonographer variables (level of experience, and type of professional) and any of the outcome measures, meaning that our overall conclusions were unchanged (data not shown).

## Discussion

This randomised controlled trial is the first to assess AI-assisted fetal ultrasound screening, including both normal and abnormal fetuses. We have shown that use of AI can significantly reduce scan duration and sonographer cognitive load while maintaining scan quality in terms of disease detection. Automatically measured fetal biometric measurements were more repeatable and reproducible compared to human manual measurements. Image quality for some planes was initially inferior using the AI tools, but this was partially ameliorated by the retrospective use of AI models to automatically select the highest quality images (although further work is needed to improve this for some planes). The trial design enabled a reasonable sample size by enriching for CHD cases, and controlled for variation in both sonographers (through the use of randomisation) and pregnant participants (all were scanned using both methods).

Previous work in the field has focused on assessment of algorithm performance using retrospective curated test datasets, which may not fully reflect the performance achieved in a real clinical environment.^6–11^ A small pilot study by our group has previously suggested a significant time saving with the use of AI, and the present study expands on this by including fetuses with known structural malformations (so that diagnostic performance of the human-AI team could be assessed), and involving a large cohort of randomised and blinded sonographers.^6^ These results are encouraging and suggest that a real clinical benefit may be offered if AI is integrated into current fetal ultrasound screening programmes.

On average, sonographers with AI assistance saved around 42% of the scan duration, potentially allowing more time to improve the overall scan experience, such as communicating with the patient or spending more time imaging a particular anatomical area of concern. Shorter scans may also yield health economic benefits by lowering cost. While this study did not assess where time savings occurred, our previous pilot study analysis found that the time savings corresponded to the periods of time where in the manual scan the screen was frozen (i.e., the time taken to measure biometry and save still images).^6^ However, it is worth noting that we have not proved in the present study that these time savings would necessarily translate into actual clinical benefit. Larger, real-world trials are required to demonstrate this benefit in clinical practice.

Our findings suggest that the AI-assisted scans were not inferior to the manual scans in terms of diagnostic performance. This is reassuring, as it implies that workflow efficiencies can be achieved without an adverse effect on sensitivity and specificity. As previously demonstrated, specificity in particular is extremely important when considering the introduction of AI to screening programmes, to avoid overwhelming downstream specialist services with false positive referrals.^22^ The fact that specificity remains robustly high in the AI-assisted group offers reassurance that AI tools may prevent this issue, potentially reducing false positive referrals for CHD. However, this study was only powered to detect large differences in diagnostic performance, with a margin of 25%. There could be smaller clinically relevant differences between the two scanning methods that we were underpowered to detect, so our results should be interpreted with caution. For this reason, we are planning a larger multi-centre trial of this technology which will have a larger study power but will require a sample size of several thousand participants.

AI-measured biometrics were found to be more repeatable and had less random error than manual measurements by different sonographers. We did identify a difference between manual and AI measurements, but accuracy remains unclear without a gold standard. It would be relatively trivial to convert the AI measurement to an equivalent of the manual measurement by subtracting the detected difference, if that were desirable. The AI measurements were based on tens or hundreds of measurements per scan using a Bayesian approach^15^, rather than the traditional approach of measuring just three times (or in many cases, just once). This resulted in a final estimate that had a far higher repeatability compared to manual measurements, as well as having the advantage of real-time feedback to the sonographer of the error range around the current estimated measurement. This method could be applied to many ultrasound-based measurements, even beyond obstetrics. By reducing random human error, we can obtain measurements that are precise, even if the scan is conducted by a different operator. Such measurements are often extremely clinically important, and by reducing variability we can be more confident about thresholds used to instigate or monitor treatments.

Despite a short training period on a novel system, AI-assisted sonographers still recorded significantly lower cognitive load scores by two different metrics compared to the manual group. Cognitive load is a concept describing how mentally challenging a given task is, and reducing it by taking over specific mundane and/or distracting tasks is a potential mechanism by which the human-AI team performance might exceed that of humans alone.^23^ Limited research exists on cognitive load changes after medical interventions and the effect on clinical practice. However, looking at the published distribution of scores across multiple fields such as air traffic control, the reduction seen here in NASA-TLX score of around 11 points is similar to a reduction from the 50^th^ to the 25^th^ centile, which is likely to correspond to a substantial perceived difference in load.^24^ The combination of reduced cognitive load and reduced scan duration shows exciting potential, and one we hope will be translated into improved fetal ultrasound screening outcomes.

Image quality results were mixed. Initially, AI-acquired images were rated lower than manually-acquired ones. However, when an improved AI model was utilised retrospectively to select the highest quality images from the same recorded examinations, this problem was solved for many of the planes. This indicates that the problem for these planes was not that high quality images were not saved, it was that the candidate images presented to the sonographers were initially not the subjectively “best” ones out of all the saved images. Our current image quality models have performed well, but some image planes still require further improvement — probably via an enlargement of the training set with further labelled images — to match the quality of manually saved images. These quality models could be integrated into the overall clinical tool and used in real time for future studies.

The main limitation of this trial was that we conducted research ultrasound scans, performed in addition to the standard clinical pathway. The sonographers were self-selected, and may not reflect the broader workforce of sonographers in terms of professional background or skill level. We have included multiple professional groups operating as sonographers in the UK, which may not be representative of all international health systems (e.g., some countries would exclusively use medical doctors to undertake this task). Although they were blinded to the CHD status of each fetus, and even that CHD was the focus of the study, they were aware that a potential malformation was present. Given each sonographer only scanned a maximum of three participants, they may have been more cautious or thorough compared to their usual clinical practice. Because of the current limitations of fetal ultrasound screening, we could not recruit pregnant participants from the standard screening population as we would not have access to a reliable ground truth. For this reason, we recruited participants who had undergone detailed fetal echocardiography, a procedure that has a much higher sensitivity and specificity than standard ultrasound screening.^25^ This means that we only included cases of CHD that had already been detected; How well AI tool assistance works in an unselected population has not yet been assessed. We also used a single model of ultrasound machine, meaning that potential domain-shift problems that may be encountered in clinical use have not yet been fully explored. There are also multiple types of CHD included in this study, which will vary in their relative likelihood of antenatal detection.

We have not addressed some broader concerns regarding medical AI in this trial, such as the potential for workforce deskilling by the automation of specific tasks. If sonographers perform fewer manual scans, skills may decline, impacting patient care if AI fails. This is an important issue and will need to be addressed if AI is to be translated to the clinical environment, perhaps by ensuring sonographers still undertake some manual scans intermittently. However, some other broader concerns such as inattention to anomalies secondary to “automation bias” have been addressed in this study, and our findings are reassuring on this front. Many risks of AI are at least partially mitigated by ensuring that the AI tool and human operator work in partnership, and that the human always retains complete control over the final interpretation of the scan findings. Translation of this study to a real-world screening-level population will be key to fully explore the utility of AI tools. Given the prevalence of congenital anomalies in the general population, this will likely require a large multi-site trial, recruiting participants as they undergo their routine clinical screening ultrasound. Enlargement of the study population in this way would also address the sample size of the present study, which is a limitation in terms of detecting more subtle effects on detection rates. However, the present study was large enough to demonstrate a significant positive effect of AI assistance. We are also working on broadening our range of views that can be automatically captured, to include all the views recommended by the International Society for Ultrasound in Obstetrics and Gynecology (ISUOG).^26^

Previous work has also explored the use of AI to directly detect anomalies such as CHD on ultrasound images.^22,27–29^ The addition of such models to our current AI tool may further improve overall scan performance, but this needs to be carefully assessed due to potential risk. However, such model ensembles may be a powerful way of improving detection of fetuses with congenital malformations and will be the focus of our future work.

In summary, we have demonstrated that AI-assistance for fetal ultrasound screening is safe and effectively reduces scan duration and cognitive load, without a reduction in diagnostic performance. This is one of the relatively few randomised prospective controlled trials of AI in medicine and raises the exciting prospect of future human-AI collaboration in this field.

## Supplementary Material

appendix_1

appendix_2

data sharing

disclosures

## Figures and Tables

**Table 1:** standard fetal ultrasound image planes and associated biometric measurements used in the study.

Anatomical area	Standard Plane	Associated biometric
*Head and neck*	Brain transventricular	Head circumference
		Biparietal diameter
	Brain cerebellar	-
*Face*	Profile	-
	Coronal lips	-
*Chest*	Four chamber	-
	Left ventricular outflow tract (LVOT)	-
	Right ventricular outflow tract (RVOT) or three vessel view (3VV)	-
	Three vessel tracheal (3VT)	-
*Abdomen*	Abdomen	Abdominal circumference
	Transverse kidneys	-
*Spine*	Coronal spine	-
	Sagittal spine	-
*Limbs*	Femur	Femur length

**Table 2:** baseline characteristics of sonographer participants after randomisation. Data are n (%) or median (IQR).

	AI-assisted scan (n=29)	Manual scan (n=29)
**Experience in fetal ultrasound (years)**	4 (3-10)	6 (2-12)
**Professional background**		
Radiographer	13 (44.8%)	13 (44.8%)
Nurse / midwife	3 (10.3%)	0
Doctor	13 (44.8%)	16 (55.2%)

**Table 3:** baseline characteristics of pregnant participants. Data are n (%) or mean (SD).

	CHD affected group (n = 26)	CHD unaffected group (n = 54)
**Maternal age (years)**	32.1 (5.4)	31.2 (6.0)
**Gestational age (weeks)**	25.3 (1.9)	24.9 (1.6)
**Body mass index (kg/m^2^)**	27.9 (5.4)	27.6 (4.6)
**Ethnicity**		
White	20 (76.9%)	51 (94.4%)
Black or mixed black	3 (11.5%)	1 (1.9%)
Asian or mixed Asian	1 (3.8%)	2 (3.7%)
Any other	2 (7.7%)	0
**Reason for referral to fetal cardiology**		
Suspected CHD	24 (92.3%)	3 (5.6%)
Family history of CHD	0	34 (63.0%)
Maternal diabetes	0	5 (9.3%)
Teratogenic medications	1 (3.8%)	3 (5.6)
Raised nuchal translucency	1 (3.8%)	6 (11.1%)
Other	0	3 (5.6%)

**Table 4:** details of fetal CHD lesions in cases.

Fetal CHD lesion	Number (%)
Right aortic arch	8 (30.8%)
Transposition of the great arteries	4 (15.4%)
Tetralogy of Fallot	3 (11.5%)
Double aortic arch	3 (11.5%
Atrioventricular septal defect	2 (7.7%)
Bilateral superior venae cavae	2 (7.7%)
Hypoplastic left heart syndrome	1 (3.8%)
Double-outlet right ventricle	1 (3.8%)
Hypoplastic aortic arch	1 (3.8%)
Pulmonary stenosis, right aortic arch, interrupted inferior vena cava	1 (3.8%)
**Total**	**26 (100%)**

**Table 5:** diagnostic performance of the two methods in detecting fetal congenital heart disease (affected group n=26, unaffected group n=52).

	AI-assisted scan (n = 78)	Manual scan (n = 78)	Difference in proportions (97.5% confidence interval)
True positive (n)	21	20	-
False positive (n)	0	4	-
True negative (n)	52	48	-
False negative (n)	5	6	-
Sensitivity (95% CI)	80.8% (60.6-93.4%)	76.9% (56.4-91.0%)	3.8% (-18.9 – 26.6%)
Specificity (95% CI)	100% (93.2-100%)	92.3% (81.5-97.9%)	7.7% (-0.6 – 16.0%)

**Table 6:** diagnostic performance of the two methods in detecting all fetal structural malformations (affected group n=27, unaffected group n=51).

	AI-assisted scan (n = 78)	Manual scan (n = 78)	Difference in proportions (97.5% confidence interval)
True positive (n)	24	22	-
False positive (n)	1	4	-
True negative (n)	50	47	-
False negative (n)	3	5	-
Sensitivity (95% CI)	88.9% (70.8-97.6%)	81.5% (61.9-93.7%)	7.4% (-15.9 – 30.7 %)
Specificity (95% CI)	98.0% (89.6-100%)	92.2% (81.1-97.8%)	5.9% (-3.8 – 15.5%)

**Table 7:** scan and reporting durations. Data are medians (IQR). * Wilcoxon signed rank test.

	AI-assisted scan (n = 78)	Manual scan (n = 78)	Mean difference
Scan duration (min)	11.4 (3.7)	19.7 (9.6)	9.3 (95% CI 7.4-11.1)
Report duration (min)	3.9 (1.7)	4.0 (2.2)	0.1 (95% CI - 0.4-0.6)
Combined scan and report duration (min)	15.6 (3.9)	24.1 (10.1)	9.4 (95% CI 7.3– 11.5)

## Data Availability

Patient-level imaging data from the trial, and the imaging data used to train the AI models are not available for sharing due to ethical restrictions. The study protocol, patient information sheet, and example consent forms are available on request. The code used to train the AI models is available on request, but the model weights used in the trial are not available.
