# Supplementary material for: Artificial Intelligence to Assist in the Screening Fetal Anomaly Ultrasound Scan (PROMETHEUS): A Randomised Controlled Trial
Source: NEJM AI. Author manuscript; Available in PMC 2026 Nov 4. (PMC7619697; doi:10.1056/AIoa2400747)
Supplement: Supplementary file 1 [file EMS218126-supplement-appendix_1.pdf]

Supplementary Appendix

This appendix has been provided by the authors to give readers additional information about their work.

Thomas G. Day, Jacqueline Matthew, Samuel F. Budd, et al. AI to Assist in the Fetal Anomaly Ultrasound Scan: A Randomized Controlled Trial. NEJM Ai. DOI: 10.1056/AIoa2400747.

Supplementary information

Contents

Supplementary information 1: development of AI models 2
Dataset 2
Standard plane classification 3
Image clustering and final image selection 3
Image quality 4
Biometrics 7
Supplementary information 2: development of clinical tool 9
Backend software application 9
Frontend tablet application 10
Frontend reporting application 11
Models used 11
Supplementary information 3: training materials for sonographers 13
Supplementary information 4: scan protocols 32
Supplementary information 5: image quality scoring 34
Results 38
Supplementary information 6: data loss 41

Supplementary information 1: development of AI models

Dataset

The data used to train the AI models used for this paper were collected as part of the iFIND project[28]. The entire videos of 9,739 routine fetal anomaly ultrasound examinations were recorded prospectively as they were performed. This dataset was named iFIND1. These examinations were conducted by over 100 professional sonographers on identical ultrasound machines (GE Voluson E8) between 2015 and 2020 in a single central London teaching hospital. During these examinations, sonographers acquired and labelled standard plane images and measured biometrics in real time. To best reflect the screening population, the scans were not selected for normality. Due to operator error, technical glitches, and patient withdrawal of consent, not all these scans could be used in the dataset, meaning 7309 video recordings of prenatal ultrasound scans were used for this study. The demographic details for the dataset are shown in Table S1

Table S1: demographic information on iFIND1 dataset

	**iFIND1 dataset**
**Maternal age (years)**	32.4 (4.8)
**Gestational age (weeks)**	20.3 (0.5)
**Body mass index (kg/m^2)**	24.8 (4.9)
**Ethnicity**	
White	6,693 (68.7%)
Black or mixed black	1,344 (13.8%)
Asian or mixed Asian	1,105 (11.3%)
Any other	452 (4.6%)
Not recorded	145 (1.5%)

To maintain patient anonymity, personal information was removed from the scan recordings and each scan was labelled with a numerical study ID, numbered sequentially. We used study IDs to create consistent training/validation/test splits across the dataset: the trailing digit determined which split each scan assigned. Scans with a study ID with a trailing digit between 0-5 were

used to train our models, 6-7 were used for validation during training, and 8-9 were held aside for testing and not used at all during training of any of our models, giving a test/validation/test split of 60%/20%/20% respectively.

To find plane labels, we automatically detected any pauses and freezes in the videos, then used OCR software to extract any text labels that were added to the frames. The text labels were then associated with standard planes, and manually checked for consistency.

Biometric annotations were also performed by sonographers in real time by placing calliper markers on the structure of interest. We extracted the locations of those callipers within the image to train our biometric networks.

Standard plane classification

We used the data and annotations described above to train an AI model to classify for fetal standard planes. We used the Sononet architecture as previously described[29] and trained it on the training labels described above, as well as "background" frames corresponding to no standard plane. This classifier obtained >90% top-1 classification accuracy in the iFIND test dataset (taken from scans with trailing digit 8-9). We named this model SonoNEXT.

Image clustering and final image selection

Because of the nature of video (20-30 images per second), there will be many valid standard view images during a scan. During live scanning SonoNEXT operates on an image-per-image basis, and thus saves multiple, often several hundreds valid images for each plane. However, only one image is required for archiving purposes. This single image can be selected by the human operator, but it is not feasible to look through hundreds of images to do this, hence some

sorting or clustering step is required to reduce the number of candidate images for the human to choose from.

To do this, at the end of the scan, for each plane all valid images were sorted into 20 clusters, using a K-means clustering algorithm. This groups images based on chronological timestamp for when the image was acquired, the output feature vector of SonoNEXT, recorded pixel size, and an image sharpness metric. The image that represents the centroid of each cluster was then selected, giving 20 images. These 20 images were then ranked, based on timestamp (later images being ranked higher), pixel size (more zoomed images ranked higher), image sharpness, and a metric defining how centred the region of interest was (defined by the SonoNEXT feature attention heatmap relative to the entire image). This ranking of images was used during the “image review stage”, after the scan is complete.

Image quality

After the trial had completed, it became apparent that the images presented to the sonographer after the clustering step described above were anatomically correct but of a lower quality than the manually-acquired images. We felt that this was because the image clustering step did not fully take into account expert-perceived image quality, and so there were likely many images of better quality that were not being presented to the sonographers, meaning an inferior quality image was ultimately chosen as the ‘best’ image. Therefore, we devised a method to automatically regress a quality score for each image of each standard plane and presented the image with the highest predicted quality. We trained a CNN to predict the clinical quality of images classified as valid standard plane. This method could then be incorporated into the clustering step for future versions of the AI tool.

Quality dataset labelling

We generated training datasets for each of our models from the outputs of the SonoNEXT standard-plane detection model. We randomly selected a single image for each standard plane from the test set of the SonoNEXT model (n=1457 subjects). There was no pre-selection step: the random selection intentionally included SonoNEXT misclassifications and poor-quality views to be representative of the images that would be passed to the quality models during scanning.

A set of objective clinical criteria were devised to evaluate the quality of each standard plane, decided by a committee of clinical experts. In addition to these criteria, we also asked labellers whether each image was overall “clinically acceptable”. We also asked them to rate the subjective quality of each image on a scale of 1-10.

Quality model training

We trained models using the same SonoNEXT architecture that we used for plane-classification to evaluate the quality of images. We modified the output layer to predict each feature that had been labelled for this standard plane, turning the network into a multitask classification network. We used cross-entropy as the loss function in training.

Image scoring and presentation

After each image for a standard plane was processed through our quality models, we selected 9 images to present to the sonographer to make a manual selection of the best one. To select the images to present, we calculated two scores for each image, which we called the quality score and the diversity score.

The quality score is a single score using a composite measure derived from the output vector for each image. Not all of the features labelled and predicted by the network are equally important: some should be given a higher weight when determining an image’s quality. To quantify the importance of each feature, we calculated a multivariate logistic regression to predict whether each image was rated “clinically acceptable” based on all the other labelled features. This had a

predictive accuracy of >95% for all standard planes. The quality score was therefore a weighted sum of the predicted features, weighted by the regressed weights associated with that plane. Each image had a quality score calculated in this way.

In a single scan, the images with the highest quality score are usually visually very similar to each other and are often consecutive frames acquired very close to each other. Presenting the images with the highest quality score for the sonographer to select from would only offer a very restricted choice. To address this, we added a diversity score, designed to be higher for images that are more different from those already presented.

We defined the diversity score D_i for each sample x_i as the lowest Euclidean norm between its output vector from the quality CNN and that of images that have already been selected

$$D_i = min\left(\left\|x_i - s_0\right\|_2, \left\|x_i - s_1\right\|_2, \dots \left\|x_i - s_N\right\|_2\right),$$

where s_0, s_1, ... s_N are the CNN output vectors of the N previously selected images. An image identical to one that has already been presented will therefore have a diversity score of 0, while all other images will have a score inversely proportional to their similarity to the most similar previously selected image.

This is an iterative process: the diversity score is initially undefined. The first image to be chosen is therefore simply the one with the highest quality score. After this, the diversity score can be calculated for all images with reference to the first selected image. This score can be updated after each successive image is selected to be presented to the sonographer.

The images presented to the sonographer were chosen based on a weighted sum of the quality score and the diversity score. The choice of weight is important: a very low weight for the diversity score is likely to result in many very similar images being presented for selection, raising the chances that none of them are clinically acceptable. A very high weight for the

diversity score will prioritise images that are very different from each other, but this will often include misclassifications and poor-quality views. We empirically decided to use a weight of 0.05 for the diversity score after manual grid search of an optimal value for this hyper-parameter.

Biometrics

Additionally, we trained AI models to measure the four key fetal growth biometrics of head circumference (HC), biparietal diameter (BPD), abdominal circumference (AC) and femur length (FL). We used the coordinates of the calliper locations to generate heatmaps consisting of Gaussian kernels centred at the calliper locations. We then trained a segmentation network using the U-Net architecture[30] to predict the heatmap for every image in the training set. We trained a separate segmentation network for each of HC, AC, and FL. BPD was not measured independently: we measured the minor axis of the derived HC ellipse to calculate it. We named these models BiometricNet-Head, BiometricNet-Abdo, and BiometricNet-Femur.

There are many frames in each scan in which biometrics can be measured. With the methods described above it is possible to extract these measurements in all frames in which the biometric is visible, resulting in hundreds or thousands of measurements per biometric. These are likely to be slightly different from each other, as the views are themselves somewhat different.

We used a Bayesian framework to aggregate all measurements into a global estimate of each biometric, which was updated in real time as more frames were visualised. With every new frame, we updated an internal model of the expected distribution of measurements. This allowed us to extract a central estimate of each biometric as well as a credible interval in which

the biometric can be expected to lie. The live estimates and credible intervals were displayed to the operator during scanning. This approach is explained in more detail in Venturini *et al*.[15]

Supplementary information 2: development of clinical tool

The clinical tool used in this study was developed in three parts:

1. Backend software application
2. Frontend tablet application
3. Frontend reporting application

Backend software application

This was developed in Python and served four purposes:

1. Read and process a live stream of ultrasound images. Images are read from a video capture card, pre-processed and passed to our SonoNEXT AI model. Outputs of this model are then used to determine which (if any) biometry machine learning model to pass to. The image is also passed to a pixel size estimator class. The outputs of the biometry and pixel size estimator are combined to give measurements in millimeters. We use this measurement to update the current estimate of each biometric.
2. Convert the analysis to JSON and stream over websocket to the frontend application during live scanning. The outputs of Step 1 are converted to JSON format and streamed over WebSocket technology to the frontend application for display.
3. Save the images and analysis to a local database (MongoDB). Every image is converted to base64 jpeg encoding, paired with the JSON from step 2 and saved to a local NoSQL database solution.
4. Expose a REST API to query the local database during reporting on both the tablet and reporting applications. The main functions are to:
    - READ images from a scan for each SonoNEXT classification, and cluster and rank these to give 9 candidate images per classification.
    - WRITE a report object for the patient to the database.

- READ the report object for a patient.
- UPDATE the report object for a patient.

Frontend tablet application

This was developed in Javascript / HTML / CSS and served the following purposes through the following UI screens:

1. 'New Scan' Screen: Input patient information to start a new scan including ID, sonographer ID, EDD (+GA is calculated automatically), the ultrasound machine being used and whether the scan was to be an 'AI assisted' scan or not. (If not 'AI assisted' then the frontend application would be blank until the end of the scan in order to capture data in the background but not display this to the sonographer).
2. 'Live Scan' Screen: The application receives live stream of image analysis from the backend via websocket and displays the following:
    - A 'Plane' card for each standard plane: Name and progress bar displaying how many images have been collected and classified as that plane.
    - A 'Biometry' card for each biometric: Name, current estimate in millimeters, place on centile for that GA and indicator of statistical validity for that measurement's current estimate.
3. 'Report' Screen: Once scanning is complete, the sonographer clicks a button to navigate to the reporting stage of the scan (in non-AI assisted scanning this button ends the scan and returns to screen 1.). Here the sonographer is shown the patient details, a list (with images) of the standard plane 'candidates' and the biometrics (with images with overlays) collected during the scan. The sonographer can click of a standard plane to choose between up to 9 alternative candidate images to save in the report instead of the initially chosen image.

Finally, the sonographer indicates if the scan was 'Normal' or 'Needs further information' before finishing the scan and returning to screen 1.

Frontend reporting application

The fronted reporting application was developed in Javascript / HTML / CSS and served the following purposed through the following UI screens:

1. 'Find report' screen: The UI displays a list of recently completed scans and the sonographer selects the scan they wish to complete the report for.
2. 'Report' screen: The UI displays the fields and biometrics collected during the scan or leaves them blank for completion if it was not an 'AI assisted' scan. The sonographer completes the remaining items on the report on this screen.
3. 'Preview' screen: The UI displays a preview of the completed report. It is then uploaded to the local database. Return to screen 1.

Technology stack

Frontend Tablet: Javascript, HTML, CSS, VueJS, CapacitorJS, NodeJS

Frontend Report: Javascript, HTML, CSS, VueJS, NodeJS

Backend Software: Python, FastAPI, WebSocket, Sklearn, ONNX, OpenCV, Numpy

Models used

The final models used in this study were:

- SonoNext v1.0
- BiometricNet-Head v1.0

- BiometricNet-Abdo v1.0
- BiometricNet-Femur v1.0

Supplementary information 3: training materials for sonographers

Prometheus
Prospective Trial of Machine Learning to Help Fetal Ultrasound Screening

Training Guide for AI-assisted scanning

Thank you for taking part in our study. Today you will be asked to perform three anomaly ultrasound examinations, assisted by our AI technology.

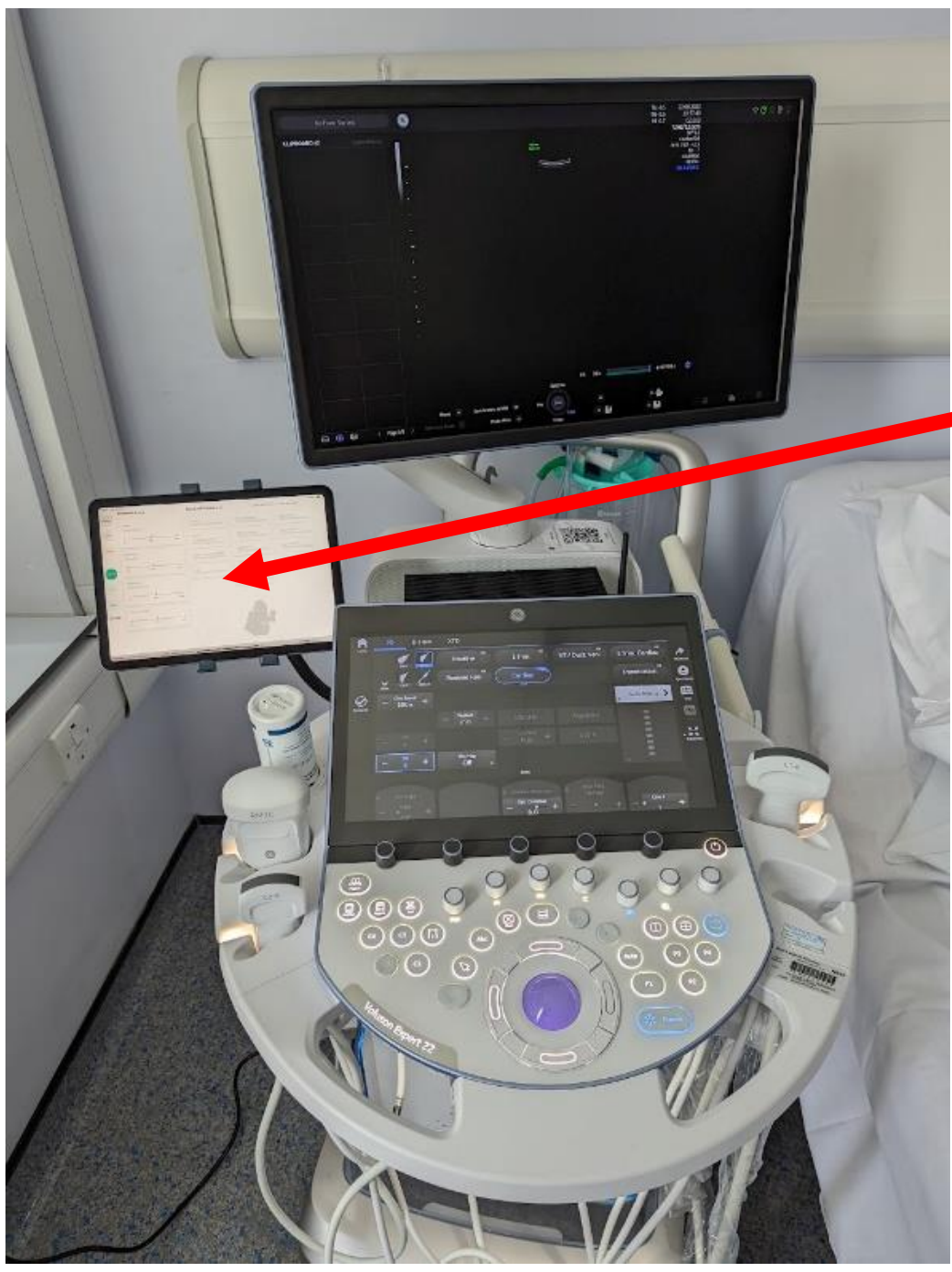

Firstly, you will see that the ultrasound has an additional iPad above the main touch screen. This is where the AI assistance will be shown to you.

The AI will do several things to help you:

1. It will automatically save standard views when you image them, meaning you do not need to freeze and save the screen.
2. It will automatically measure biometric paremeters, meaning you do not need to measure them manually.
3. It will automatically pass the standard views and biometric measurements to a report, so you do not have to type these out.

The iPad screen will look like this when you start the scan:

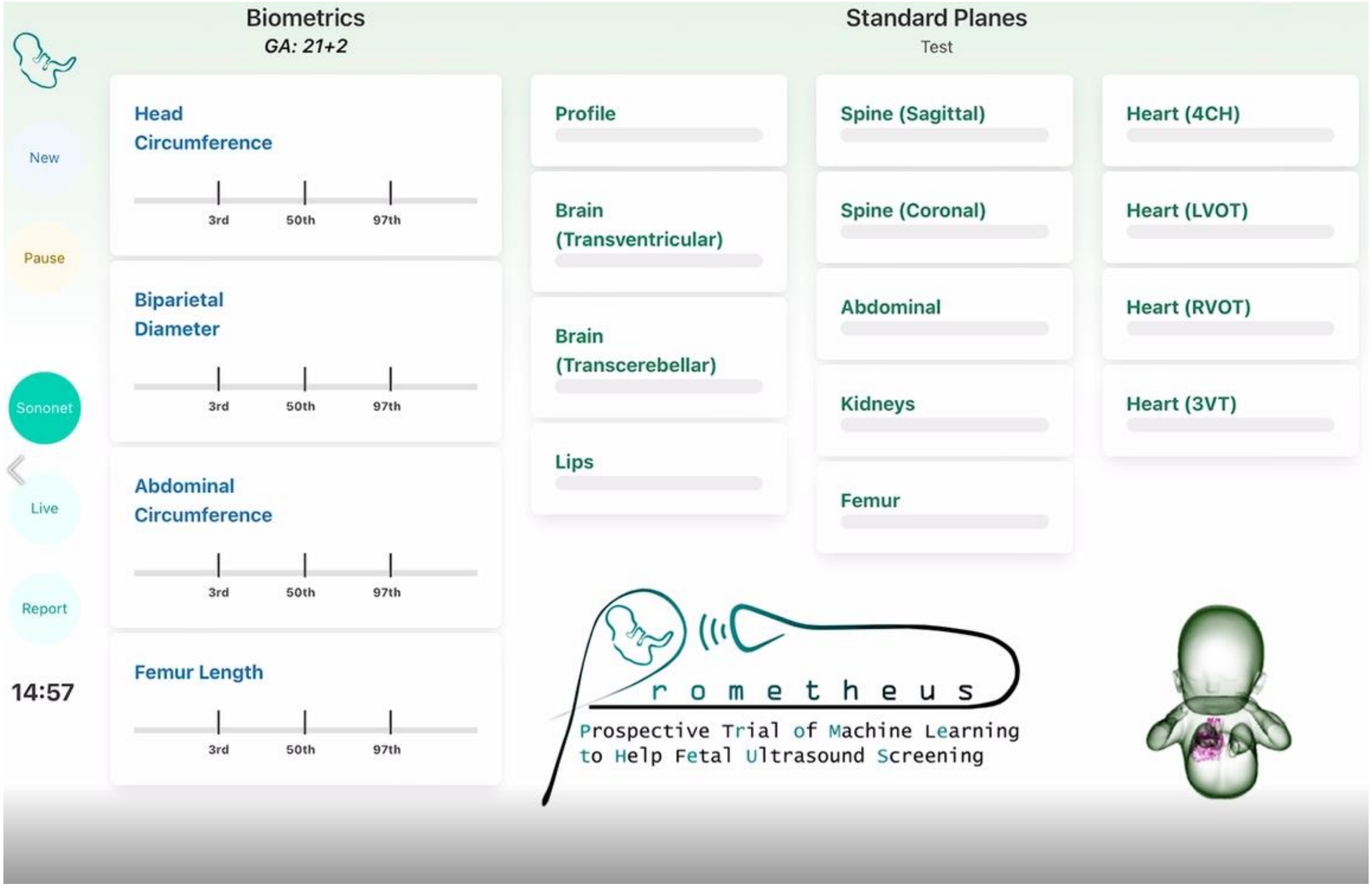

Standard images

The 13 standard images are shown on the right of the screen, with green text. Each box refers to a different standard image, for example the 4-chamber heart view:

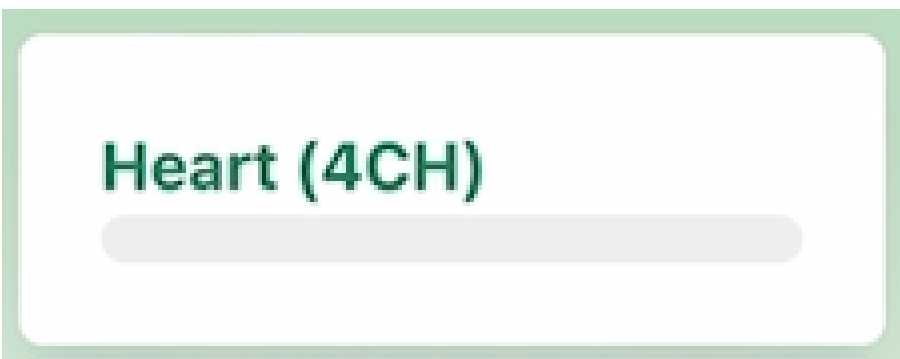

The AI is constantly analysing the stream of frames it is receiving from the ultrasound machine. As soon as it detects that you are imaging a standard view, for example the 4-chamber view, that box will change colour to green:

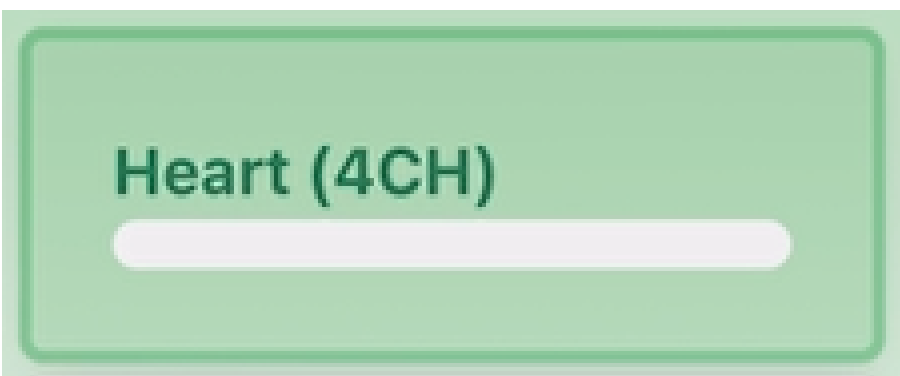

When *any* standard image is being saved, the whole iPad screen will also change to a shade of green.

The rotating baby model in the bottom right corner of the screen also indicates what image is being save, by marking the plane on the baby in light blue. For example, this shows that the femur is being imaged:

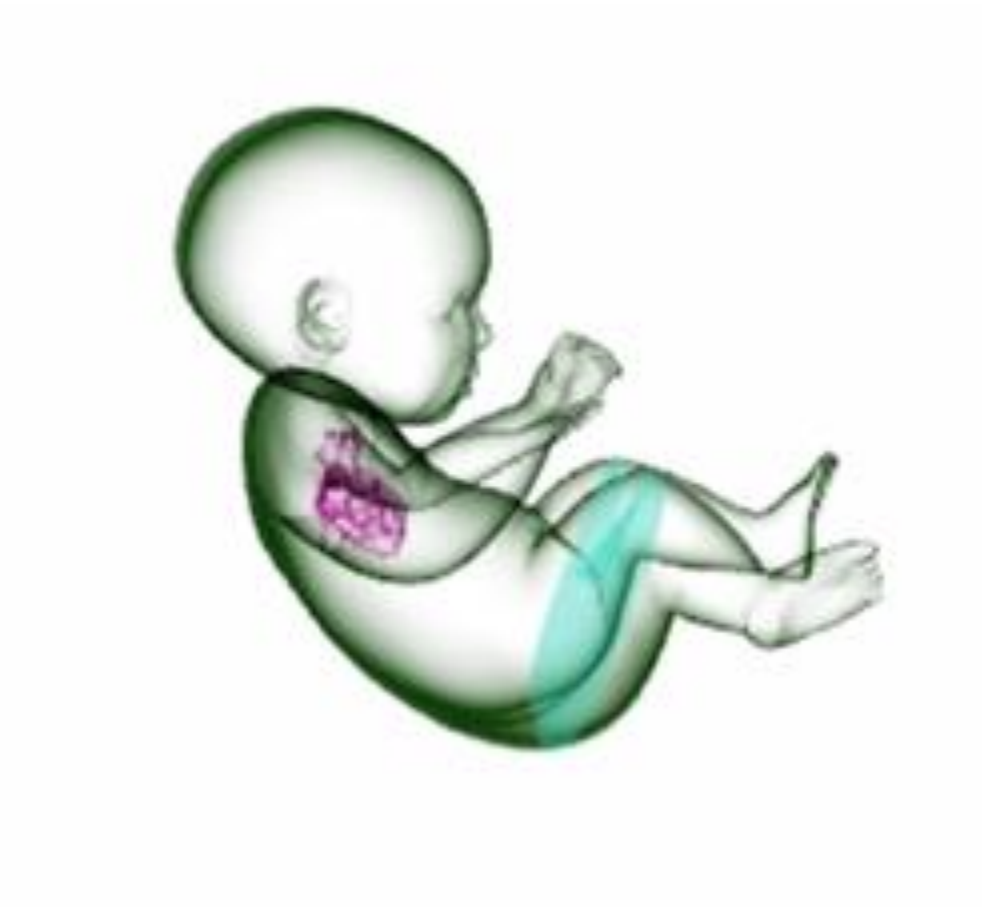

The progress bar below the name of the standard view is there to show how many images have been saved. When at least 10 images is saved, the bar will turn light green. As more images are saved, dark green lines will be drawn on the bar. The more images that are saved, the more dark green lines are drawn:

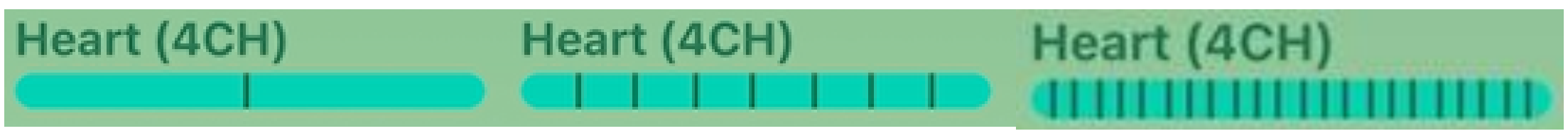

Some images saved → *more images saved*

Eventually the progress bar will turn completely dark green:

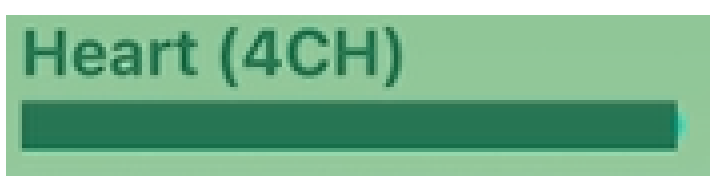

More images will **still continue to be saved** if they are detected. The progress bar is there only as a guide to you. You should keep scanning until:

- You feel you have adequately visualised all the standard views, **AND**
- The progress bar indicates that *at least some* images have been saved for each, like this:

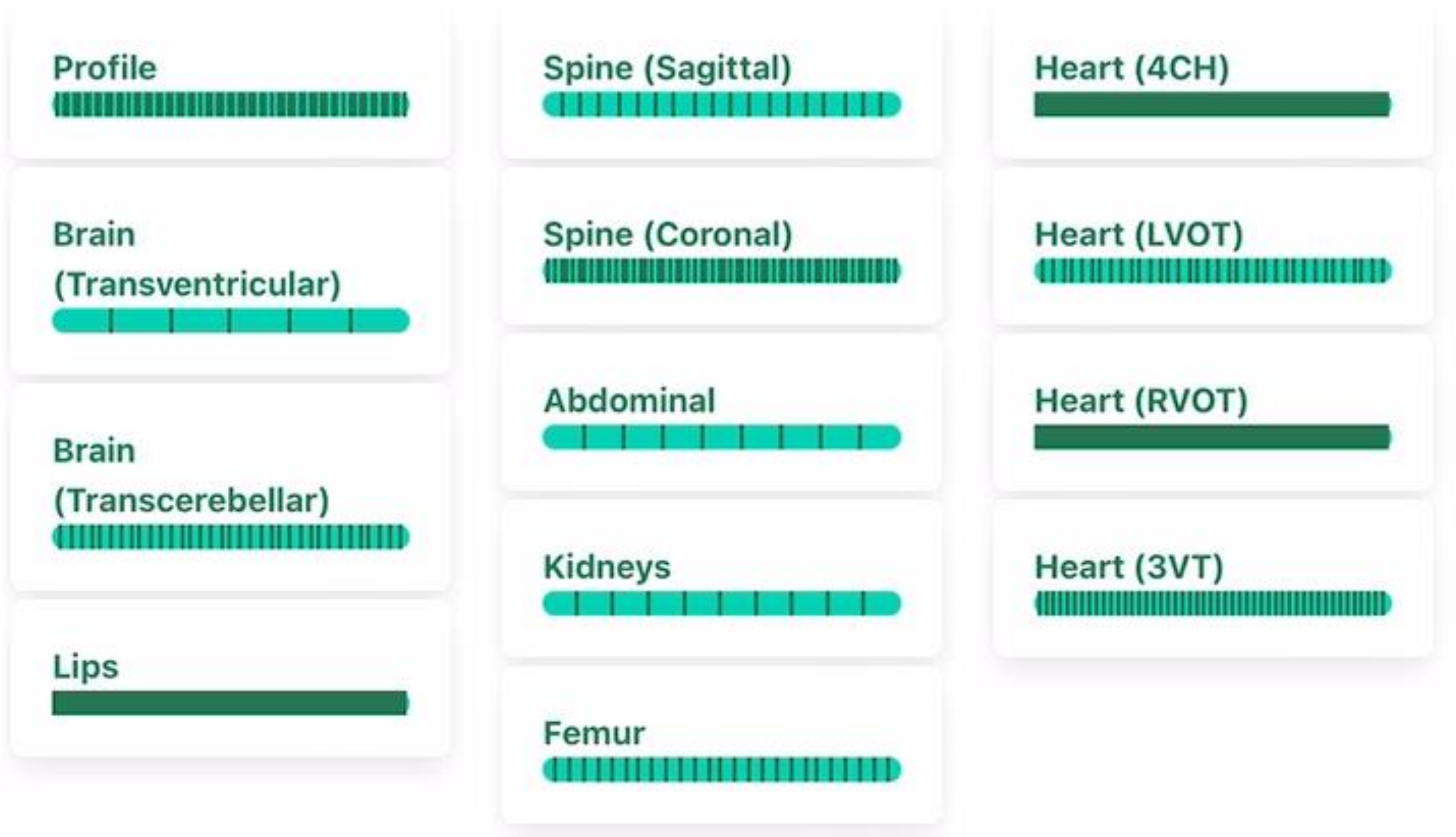

However, if you feel that it is not possible to visualise any of the standard images (for example because of difficult fetal lie), then you can stop attempting that image. There will be a chance to document this later.

Example images and ideal criteria of the 13 standard images required are outlined below.

Brain (transventricular)

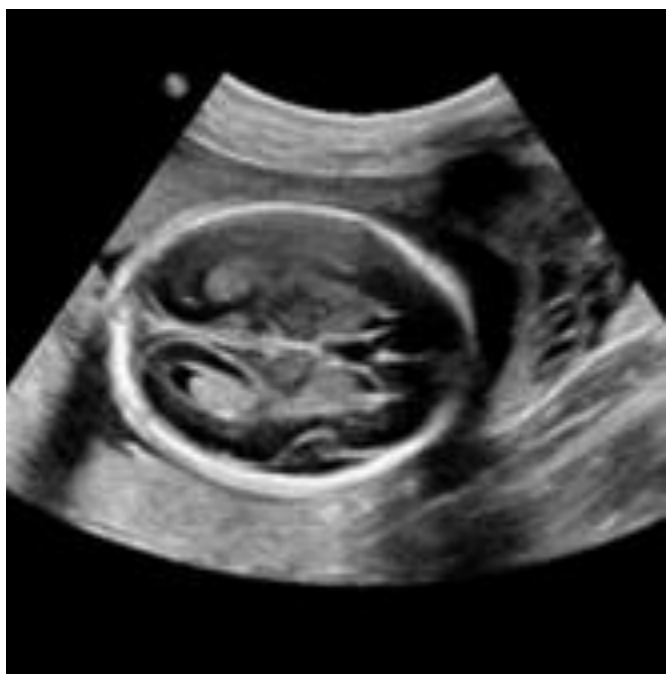

- Symmetrical plane with the midline echo of the falx dividing the skull
- Cavum septum pellucidum is visible 1/3 of the way along the midline falx
- Posterior ventricular horn visible at the level of glomus of choroid plexus
- No cerebellum visualised
- Clear delineation of intra-cranial structures
- Magnification at least 30% of the screen

Brain (transcerebellar)

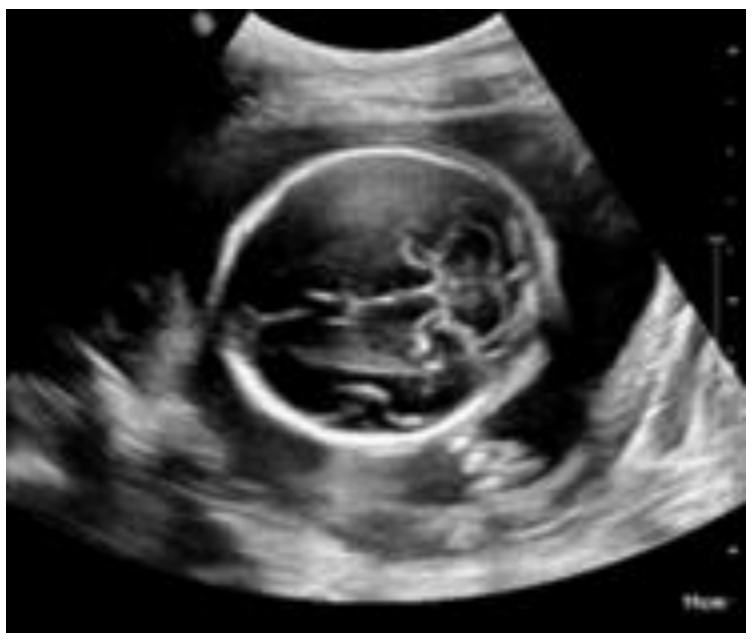

- Symmetrical plane with the midline echo of the falx dividing the skull
- Cavum septum pellucidum is visible 1/3 of the way along the midline falx
- Equal sized cerebellar hemispheres visualised
- Posterior fossa and occipital bone structures visualised
- Clear delineation of intra-cranial structures
- Magnification at least 30% of the screen

Abdomen

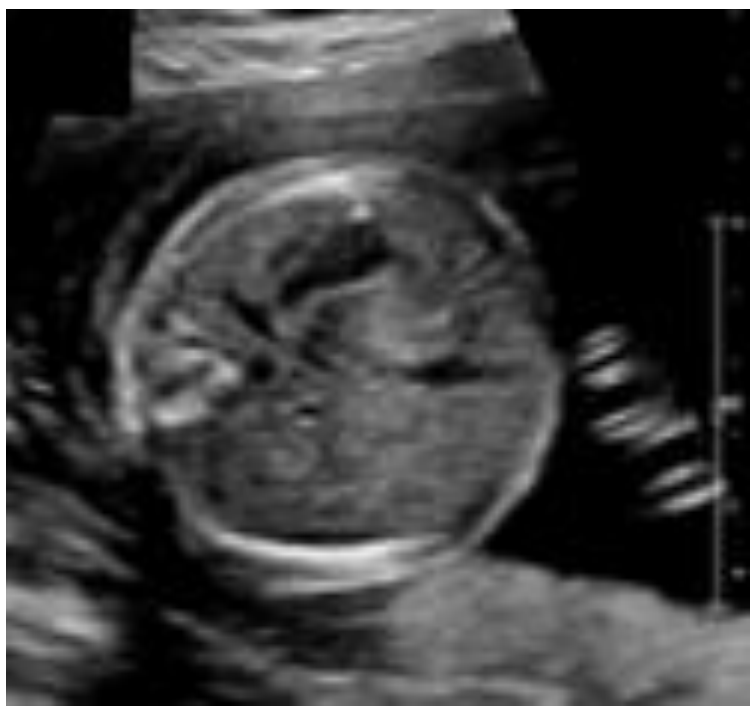

- Transverse view (spine at 3 or 9 o'clock on image)
- Stomach bubble visualised
- Anterior 1/3 of umbilical vein visualised at the level of the portal sinus (hepatic portion)
- Single complete rib included
- Kidneys/bladder not visualised
- Magnification at least 30% of the screen

Femur

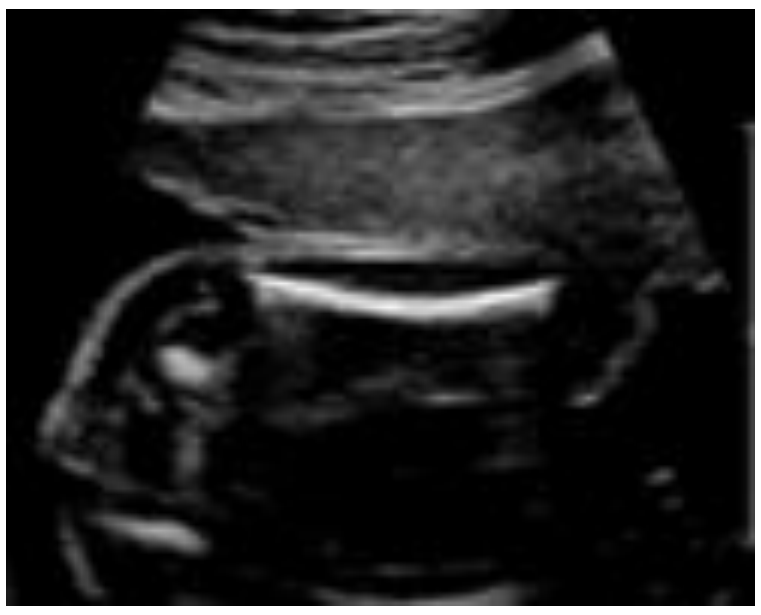

- Angle of insonation between 45 and 90 degrees
- Both ends of the ossified metaphysis visualised
- Magnification at least 30% of the screen

Spine (sagittal)

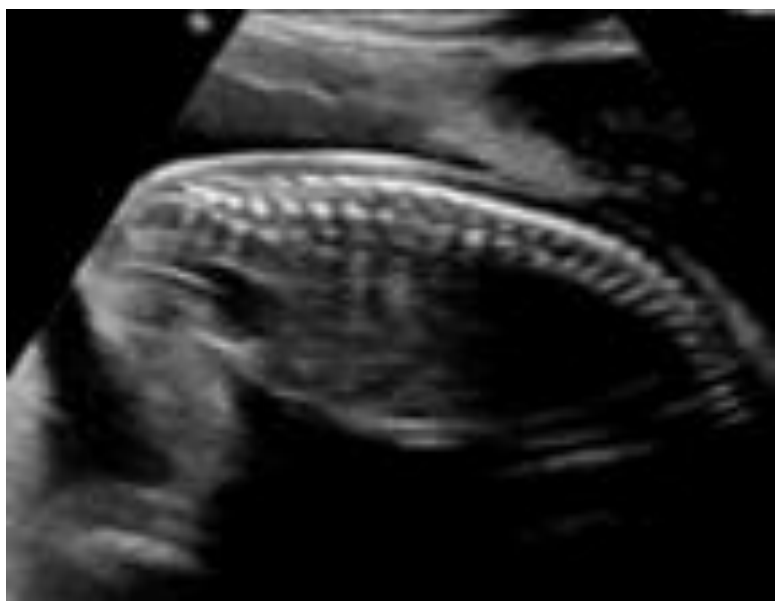

- Mid-sagittal view from neck to sacrum
- Alignment of cervical, thoracic, lumbar and sacral vertebrae visualised for assessment
- Intact posterior skin edge visible
- Amniotic fluid visible beyond the skin
- Magnification at least 30% of the screen

Spine (coronal)

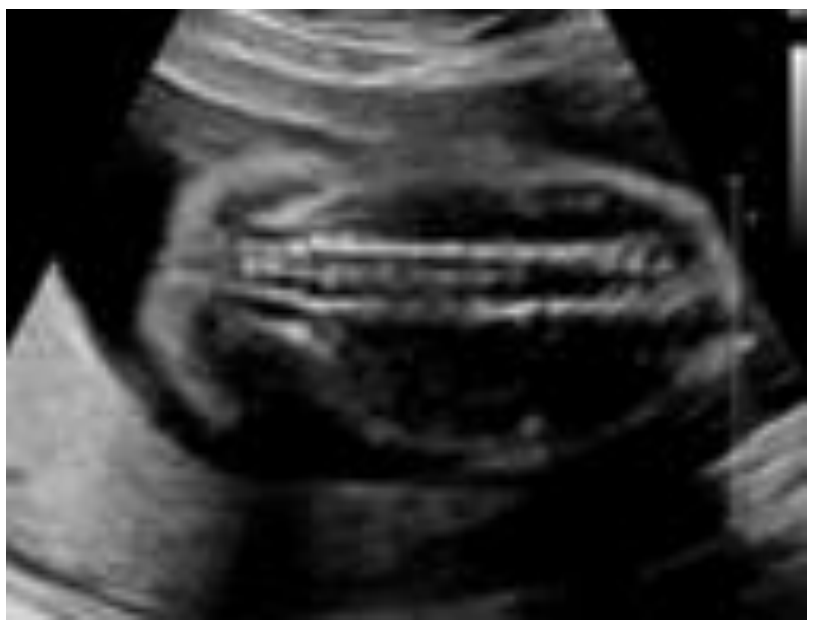

- Coronal view from cervical region to thoracic spine and lumbar region to sacrum (to include iliac bones)
- Alignment of cervical, thoracic, lumbar and sacral vertebrae visualised for assessment
- Magnification at least 30% of the screen

Lips

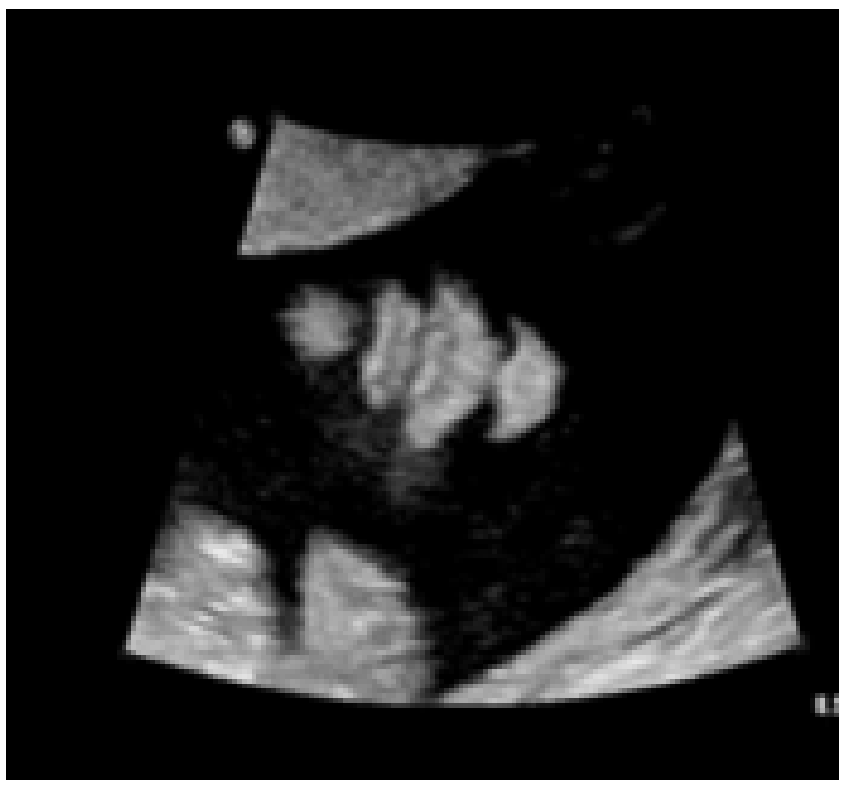

- Coronal view
- Upper lip (and ideally lower lip) visualised
- Two lip angles visualised
- Nasal tip visualised
- Both nostrils visualised
- Magnification at least 30% of the screen

Profile

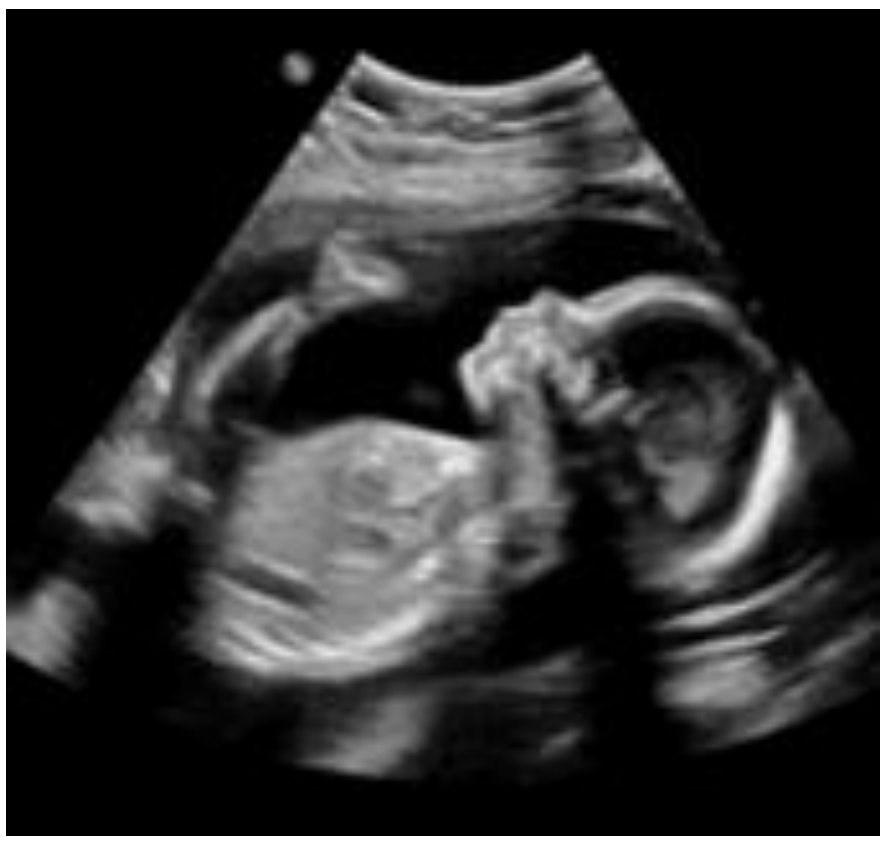

- Mid-sagittal view
- Whole fetal head and proximal 1/3 chest included within image
- Amniotic fluid visible beyond skin/beneath chin for clear delineation of facial structures
- Forehead, nasal bone, nasal tip, lips and chin visualised
- Corpus callosum/cavum septum pellucidum visualised
- Magnification at least 30% of the screen

Kidneys

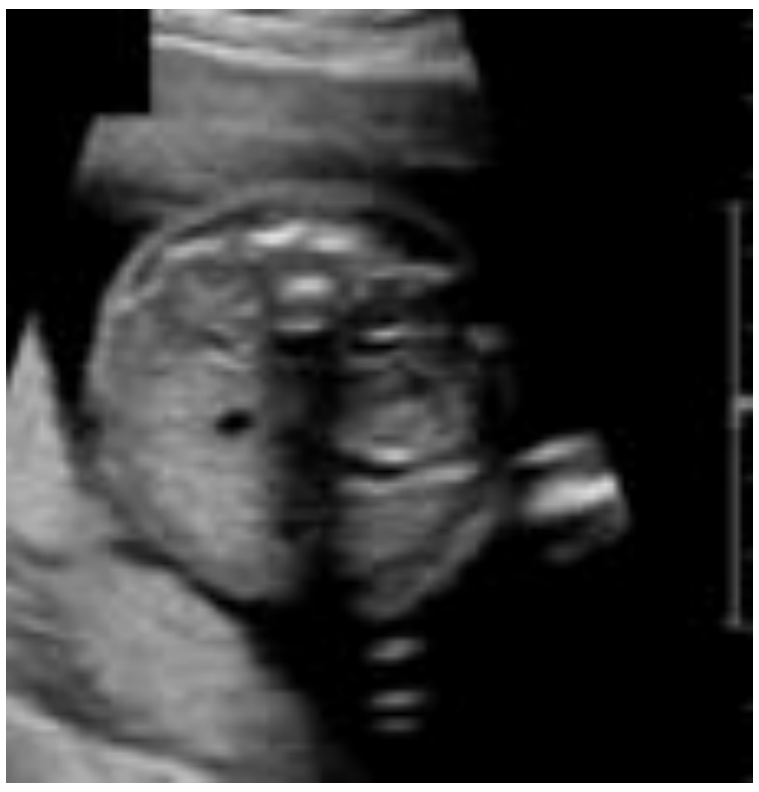

- Transverse view
- Spine at 12 o'clock
- Both kidneys visualised adjacent to spine
- Corticomedullary differentiation or renal pelvis visualised for both kidneys
- Magnification at least 30% of the screen

Heart (4-chamber)

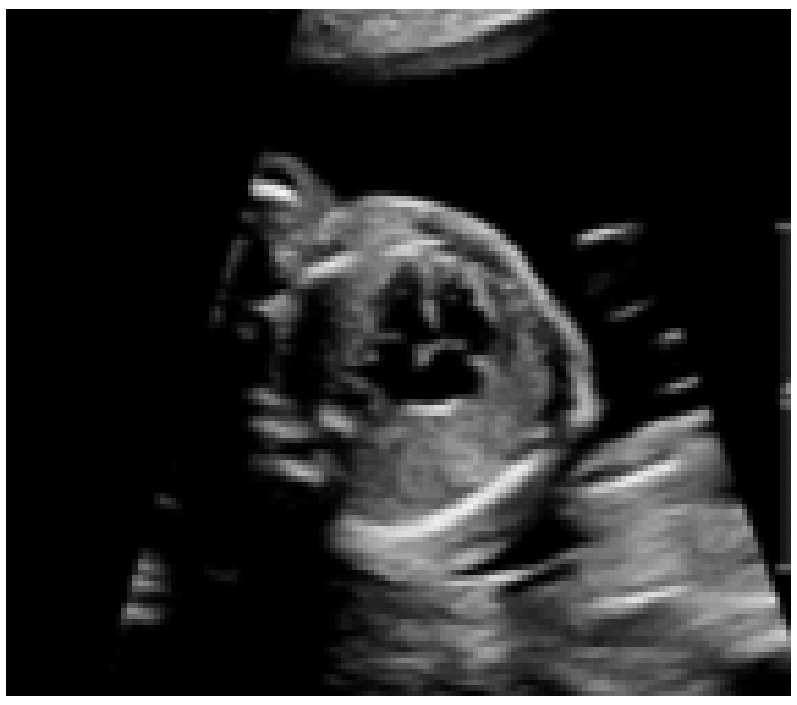

- Transverse view of heart and chest
- Equal sizes right and left atria/ventricles
- Cross-sectional view of descending aorta (and ideally one pair of pulmonary veins) visualised
- Atrio-ventricular valves visualised (open/closed), offset at crux noted
- Intraventricular septum visualised from crux to apex (no drop out artefact)
- Magnification at least 30% of the screen

Heart (left ventricular outflow tract)

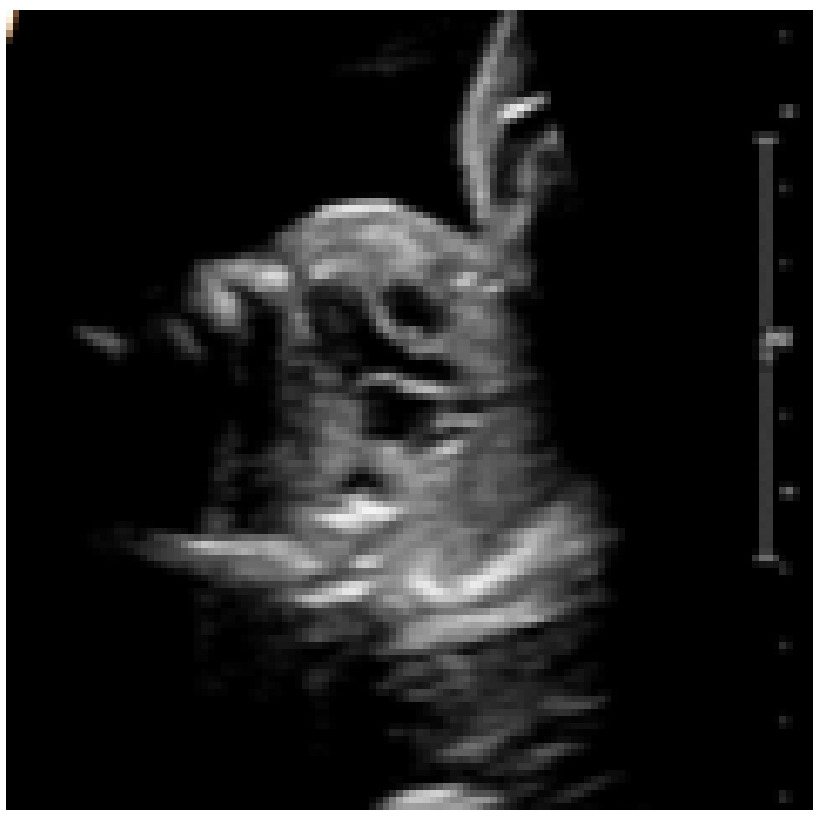

- View of heart and chest
- Aorta continuous with left anterior ventricular wall/IVS showing aortic root/aortic valve (open/closed)
- Left ventricle, right ventricle, left atrium and cross-section of descending aorta visualised
- Magnification at least 30% of the screen

Heart (right ventricular outflow tract or three vessel view)

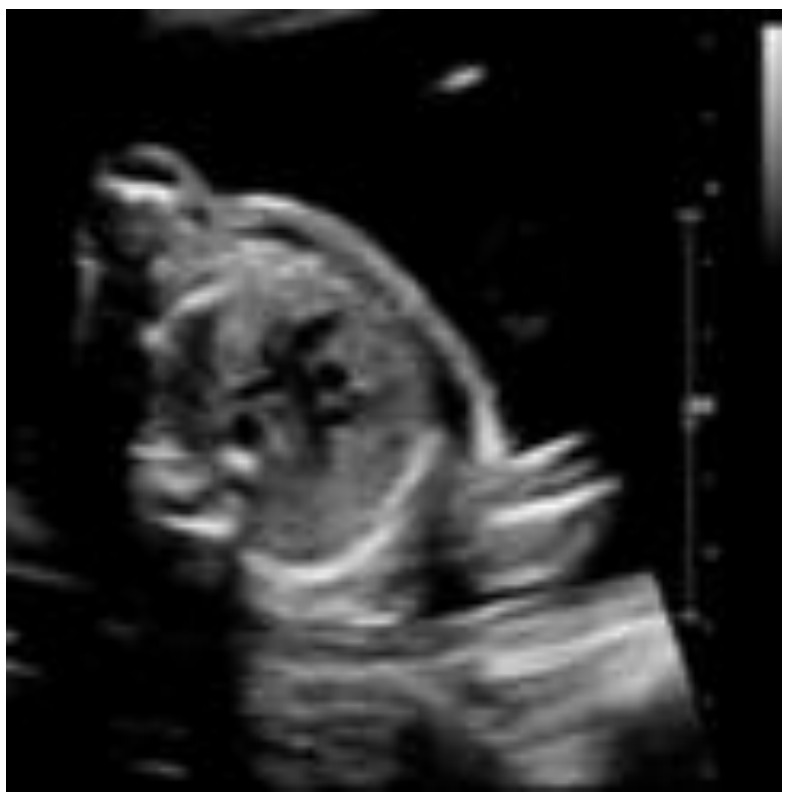

- View of heart and chest
- Pulmonary artery / duct seen in long axis
- Aorta and SVC seen in short axis

Or

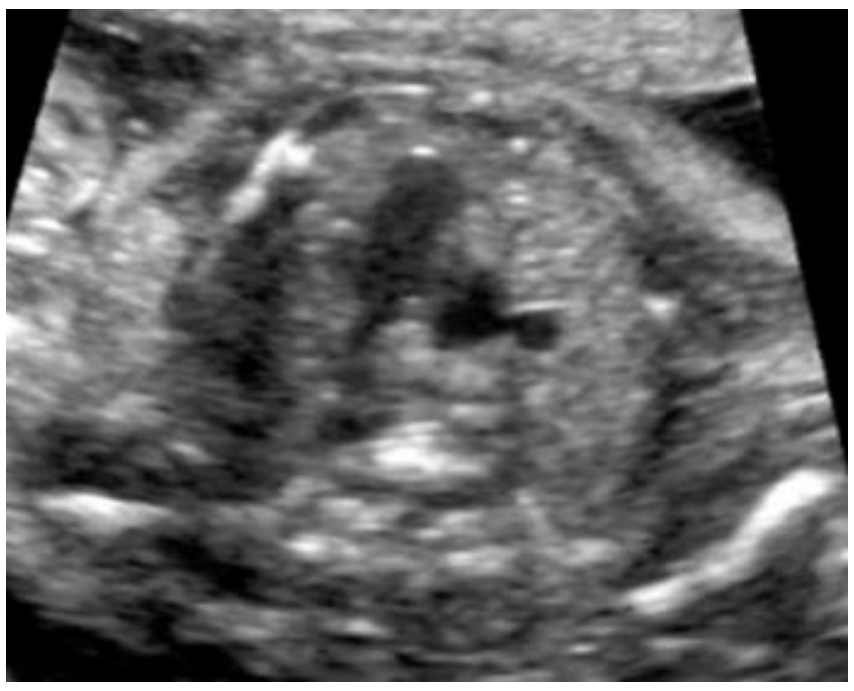

Heart (three vessel tracheal view)

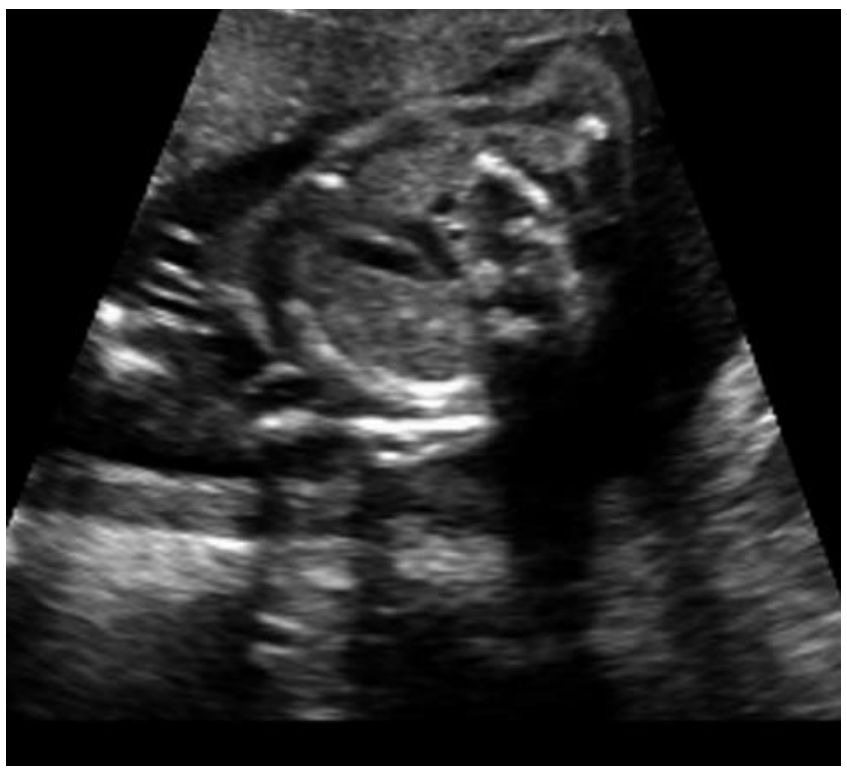

- View of heart and chest
- Pulmonary artery / duct seen in long axis
- Aorta seen in long axis
- SVC seen in short axis

Biometry

The AI automatically measures the following four biometric paramters:

1. Head circumference
2. Biparietal diameter
3. Abdominal circumference
4. Femur length

The biometrics will be measured for every relevant frame, meaning that by the end of the scan likely hundreds of measurements will be taken into account. The biometric is shown to you on the left side of the iPad screen:

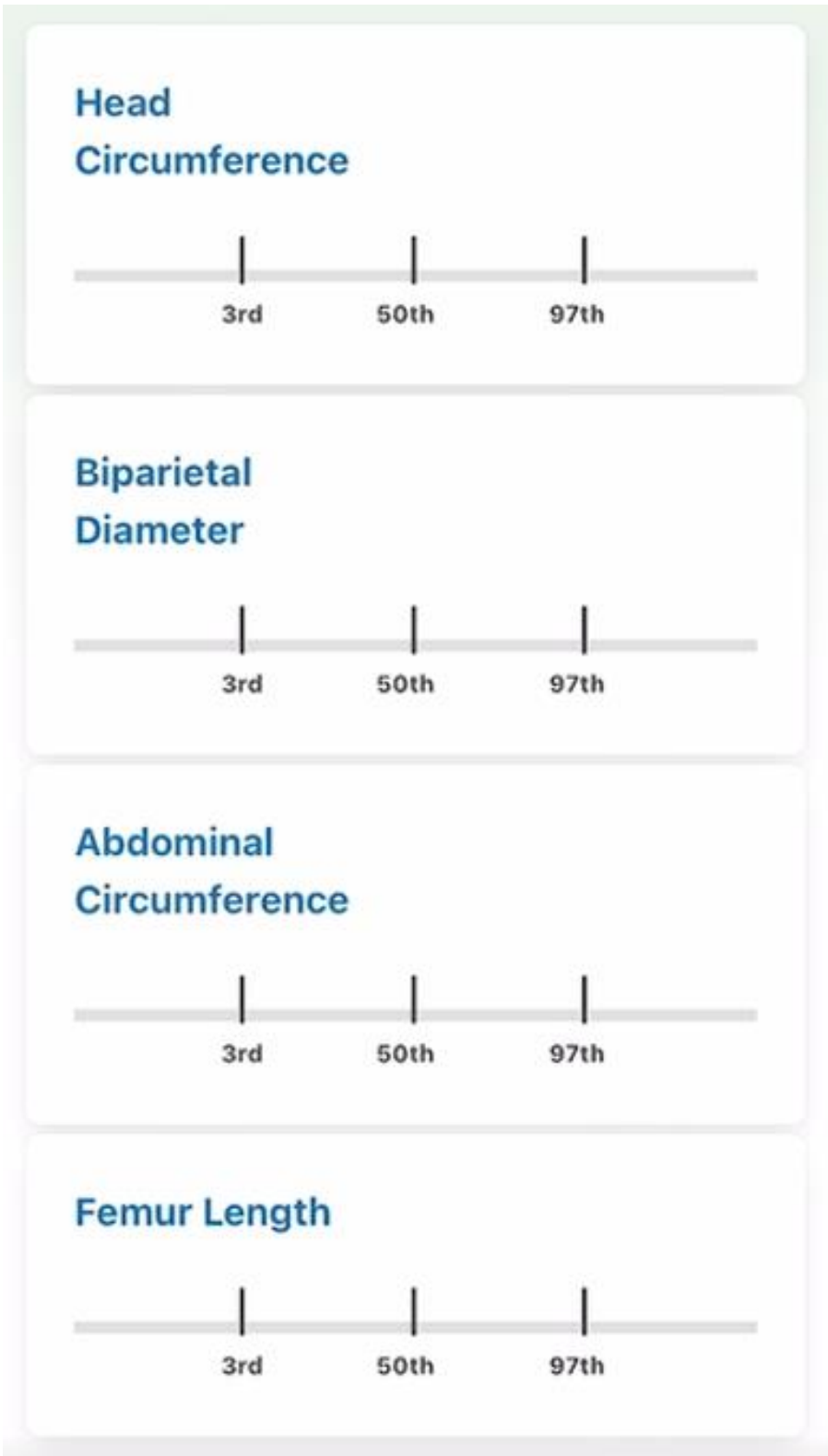

The diagrams below each biometric are there to show the normal expected range of each measurement for the given gestation. "3rd, 50th, and 97th" refer to the 3rd, 50th and 97th centile respectively. Once the AI has measured some examples of the biometric, it will put the measurements on the diagram, so you can see how they correspond to normal:

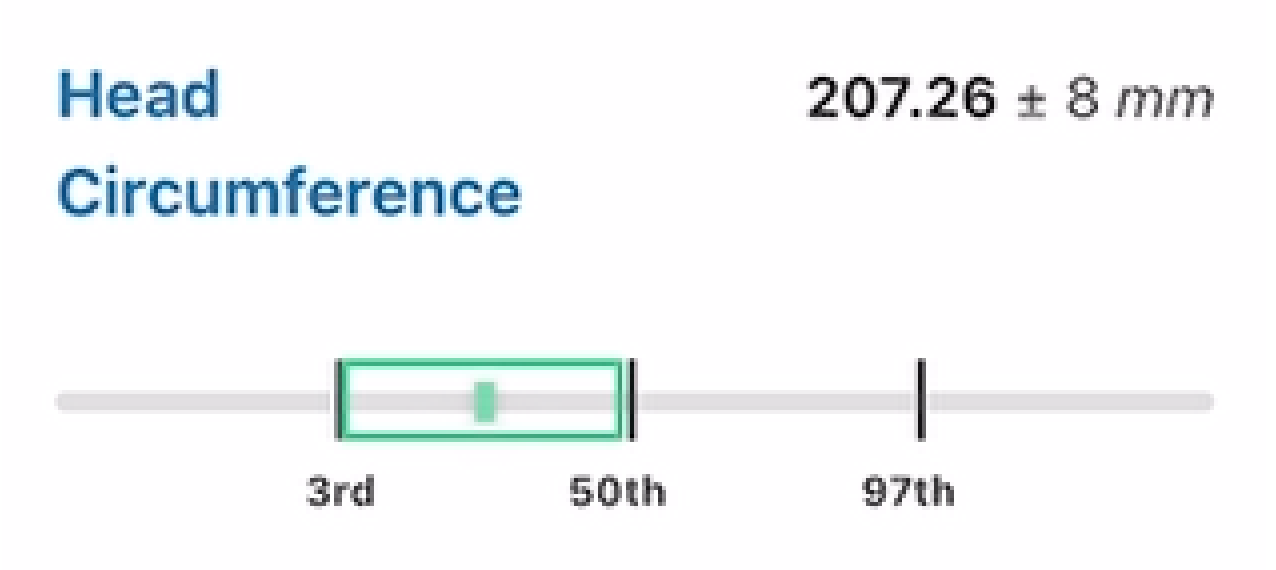

The green line in the middle of the box is the best estimate of the AI for the measurement. This is taking into account *all* the images it has seen. The green box shows how different the measurements are from each other. The bigger the green box, the more uncertain the AI is about the estimated measurement. As you scan the relevant plane for longer, the green box will get smaller (as the AI becomes more certain). Eventually, the AI will put a green tick in the corner of the measurement:

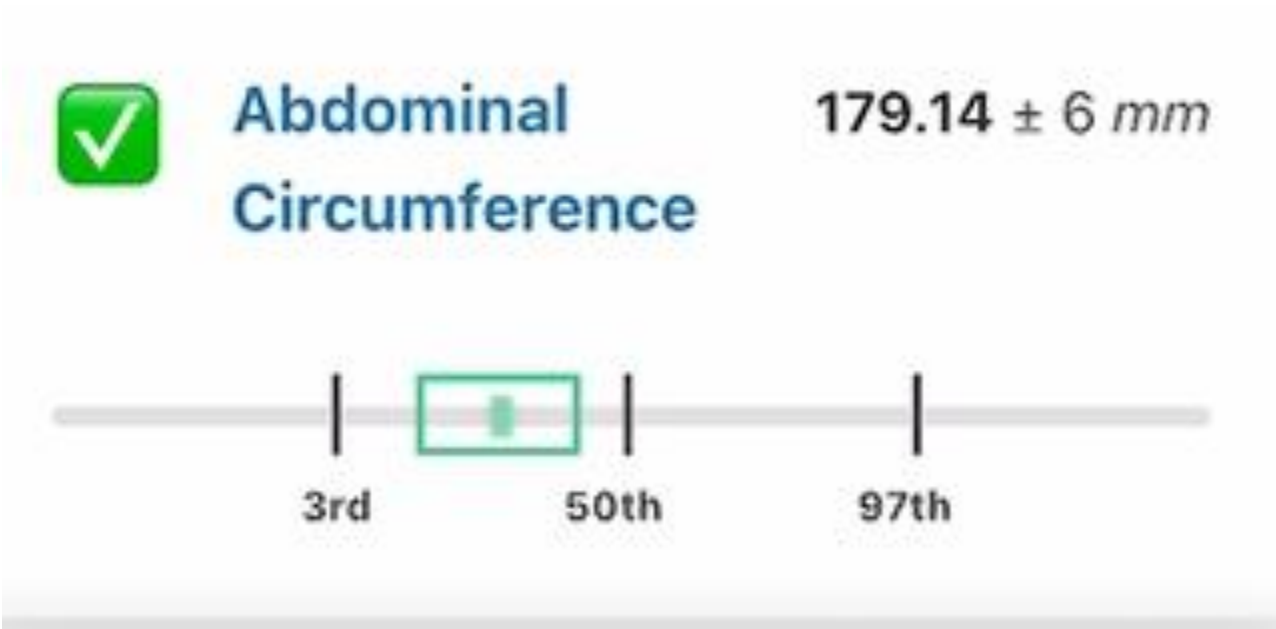

This means that it is happy it has seen enough images to make a good parameter. You should try (if technically possible) and keep scanning until all four biometric measurements have green ticks in the corner. If there are issues (such as fetal lie) that mean you can't image a certain biometric, then you can stop attempting and document this later.

Image review

When you have finished scanning, you should press the 'report' button on the left of the iPad:

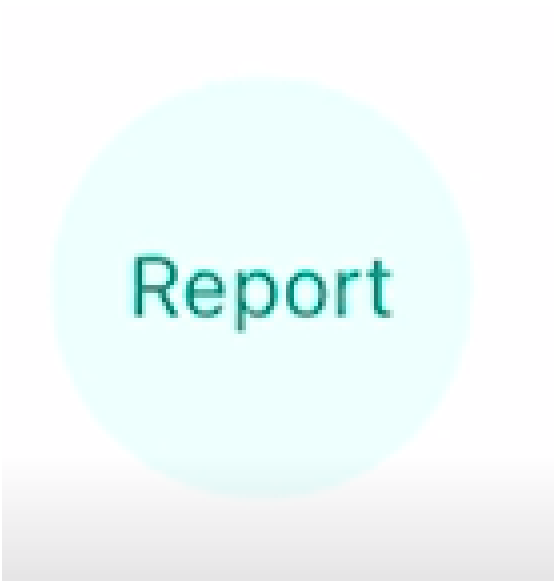

This will then take to the image review stage:

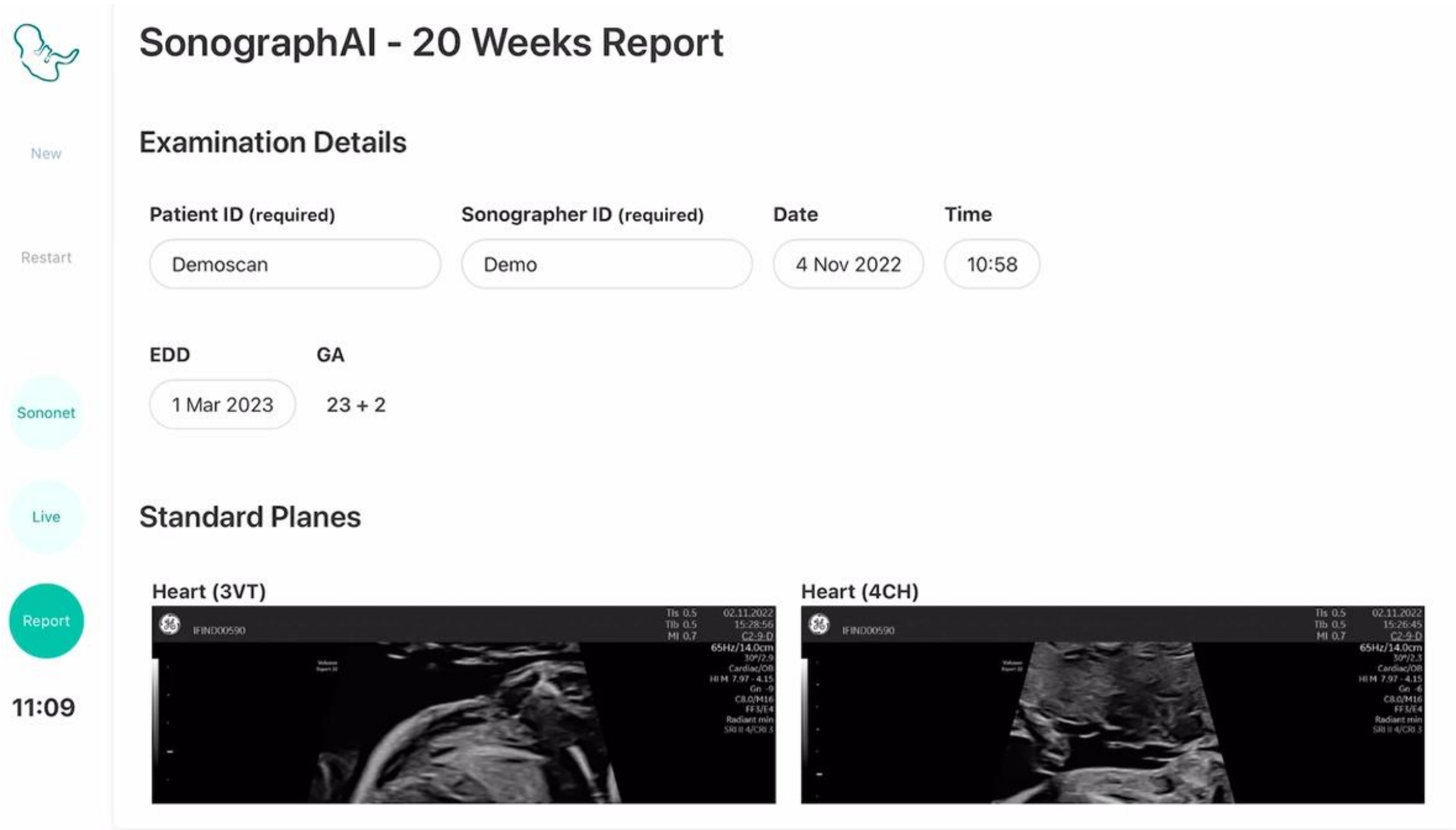

This shows you a candidate image for each of the standard images. If you feel the displayed image is adequate, you do not need to do anything. If you feel it is not adequate, you should click on the image. When you do this, more candidate images will be shown to you:

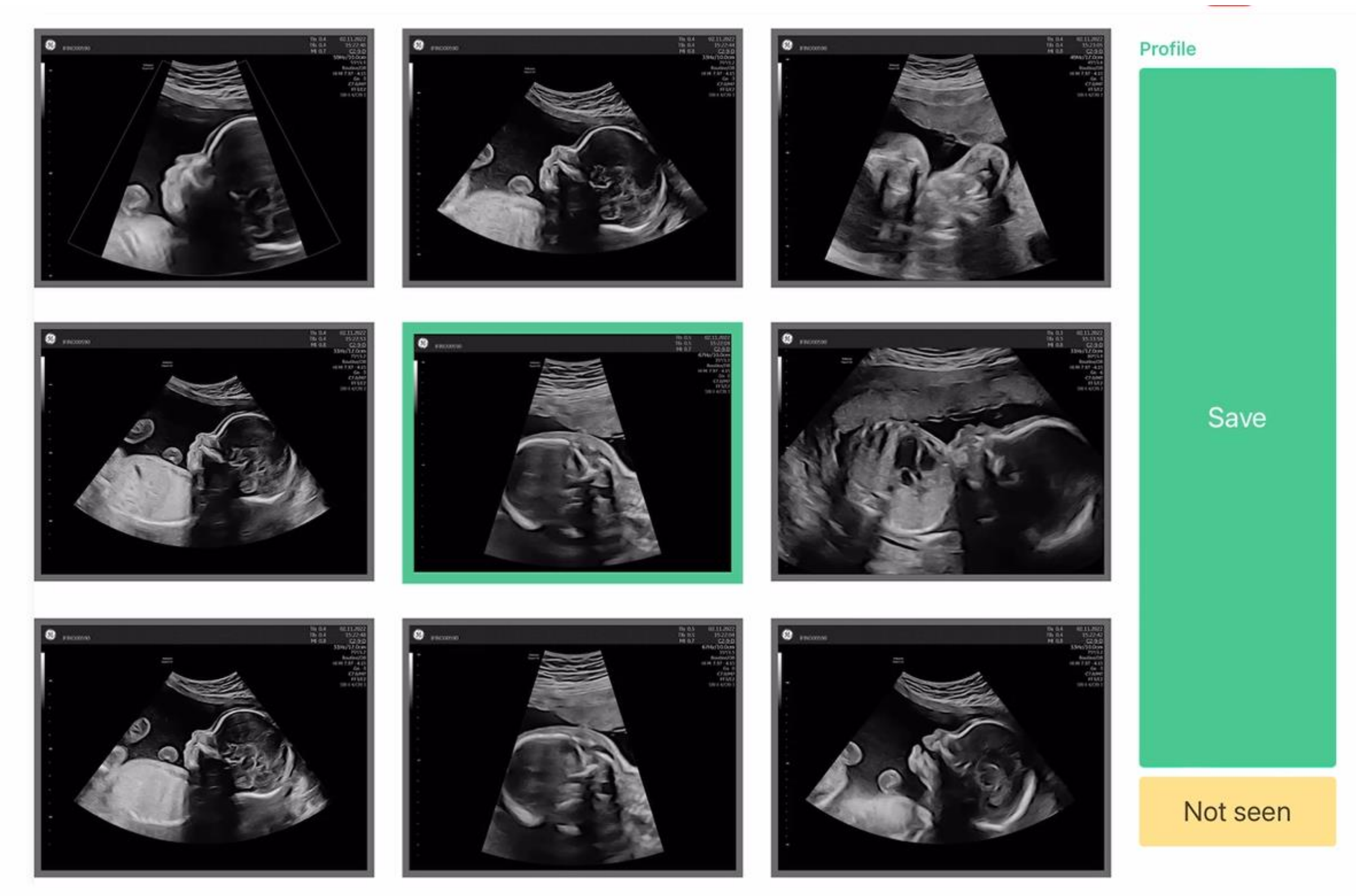

You should select the best image by clicking on it. The selected image is indicated by a green box around the image. When you have selected the best image, you should click 'save' on the right side of the screen. If there is no good candidate image, and you feel that this is because you were not able to image that area of anatomy well (for example because of fetal lie), you should click the 'Not seen' button on the right of the screen.

The image you select will be the image sent to PACS for other clinicians to review if they need to. However, **all the images** that have been saved by the AI (which will often be in the hundreds) will be stored by the AI in case they are needed later.

You will also be shown four images indicating to you how the biometric measurements have been measured, and plotted on the normal range:

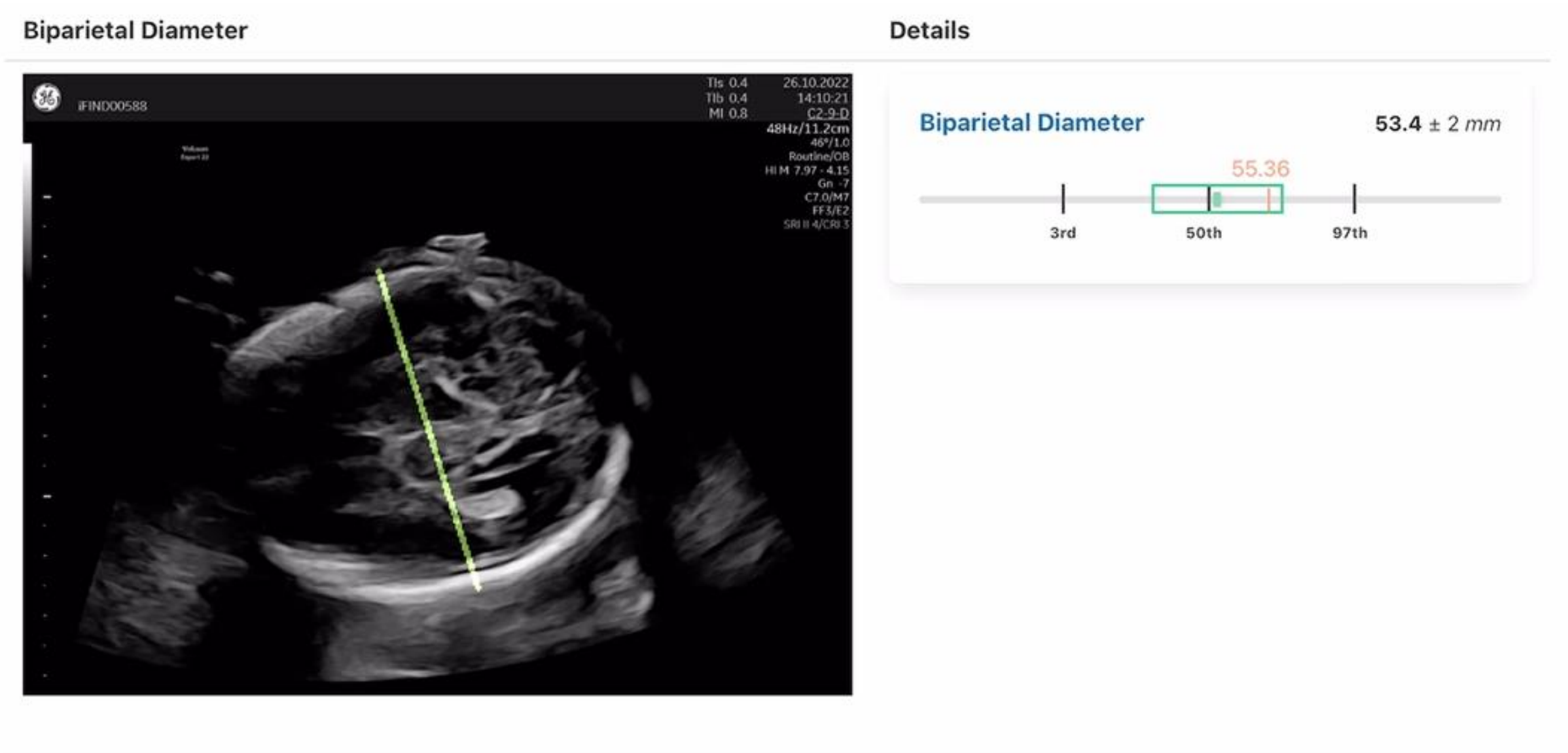

The black number at the top right shows the best estimate of that biometric parameter and the standard error (a measure of how variable the measurements are), and these are also plotted on the chart. The best estimate is shown by the vertical green line, and the standard error is shown by the green box. The orange number and vertical line show the actual measurement that corresponds to the image on the left.

After you have reviewed all the standard images, you should fill out the drop-down box at the bottom of the screen:

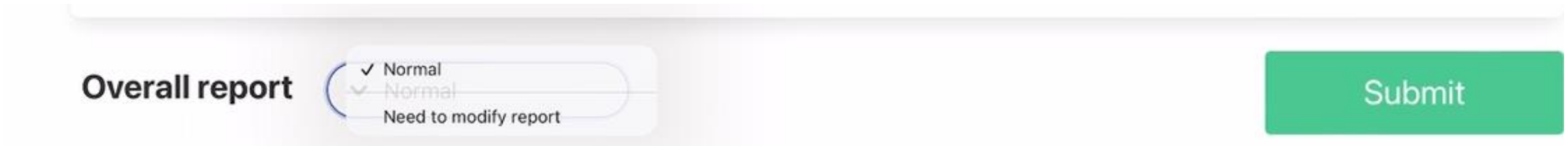

Then click ‘submit’.

Prometheus
Prospective Trial of Machine Learning to Help Fetal Ultrasound Screening

Training Guide for manual scanning

Thank you for taking part in our study. Today you will be asked to perform three anomaly ultrasound examinations, in the standard way (without using our AI technology).

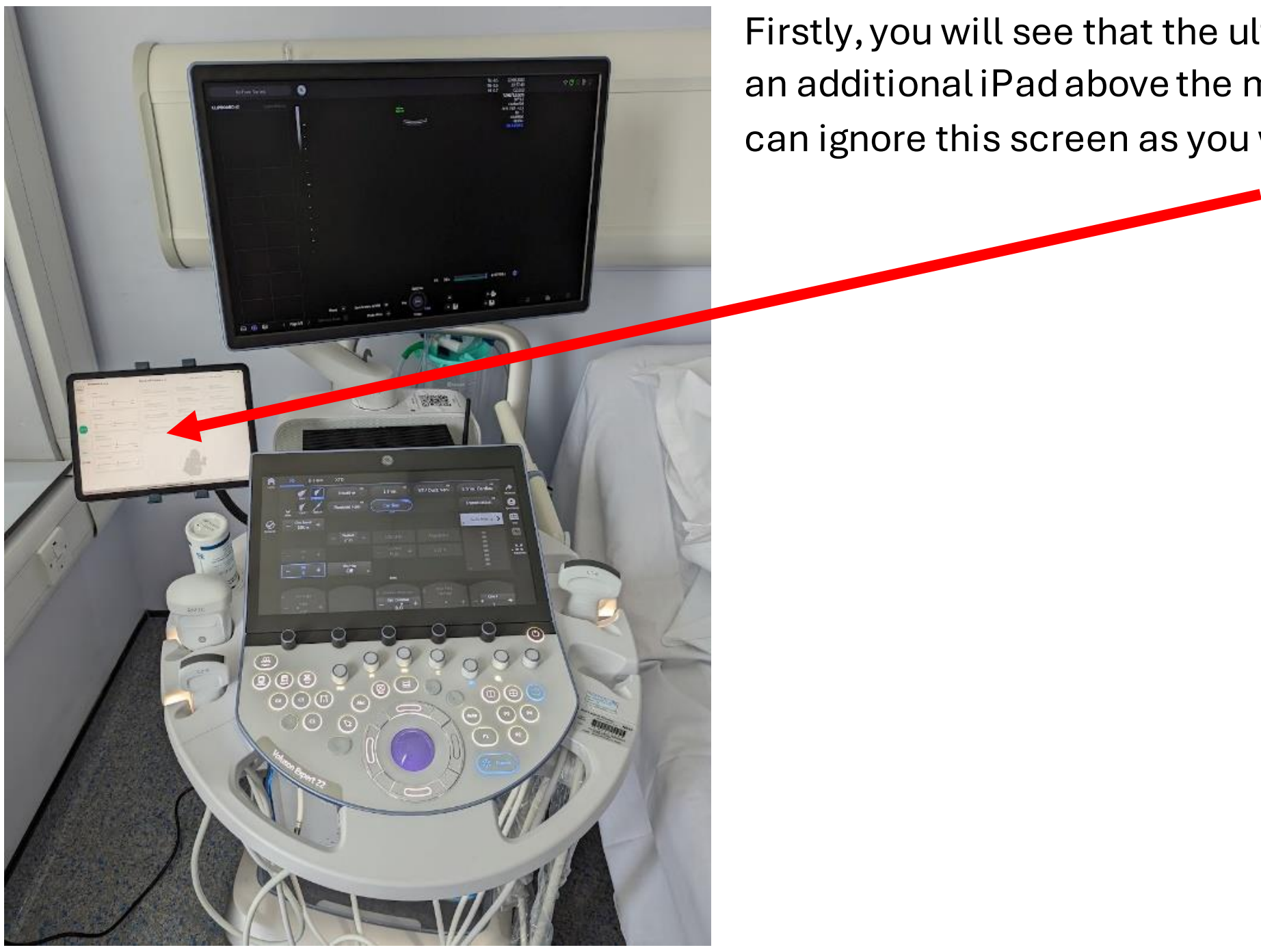

Firstly, you will see that the ultrasound machine has an additional iPad above the main touch screen. You can ignore this screen as you will not need to use it.

Standard images

As part of this research ultrasound scan, you need to record 13 standard images.

Example images and ideal criteria of the 13 standard images required are outlined below.

Brain (transventricular)

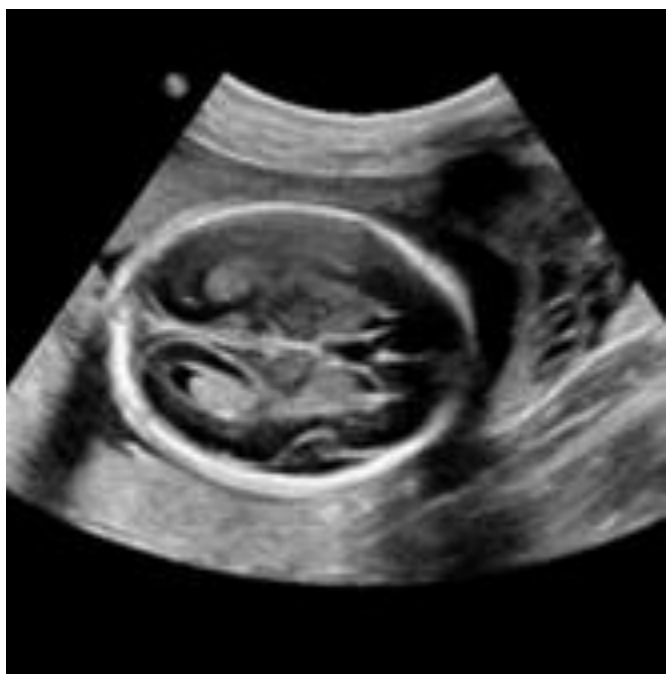

- Symmetrical plane with the midline echo of the falx dividing the skull
- Cavum septum pellucidum is visible 1/3 of the way along the midline falx
- Posterior ventricular horn visible at the level of glomus of choroid plexus
- No cerebellum visualised
- Clear delineation of intra-cranial structures
- Magnification at least 30% of the screen

Brain (transcerebellar)

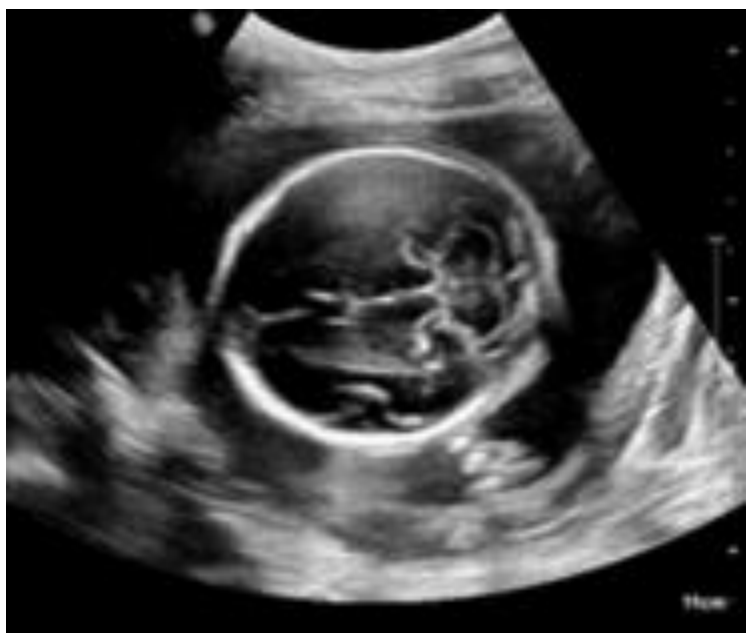

- Symmetrical plane with the midline echo of the falx dividing the skull
- Cavum septum pellucidum is visible 1/3 of the way along the midline falx
- Equal sized cerebellar hemispheres visualised
- Posterior fossa and occipital bone structures visualised
- Clear delineation of intra-cranial structures
- Magnification at least 30% of the screen

Abdomen

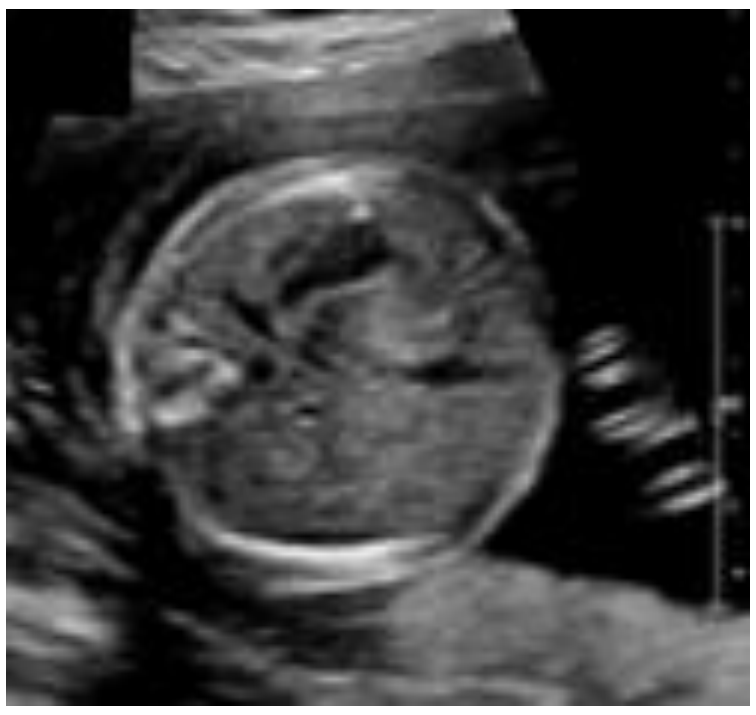

- Transverse view (spine at 3 or 9 o'clock on image)
- Stomach bubble visualised
- Anterior 1/3 of umbilical vein visualised at the level of the portal sinus (hepatic portion)
- Single complete rib included
- Kidneys/bladder not visualised
- Magnification at least 30% of the screen

Femur

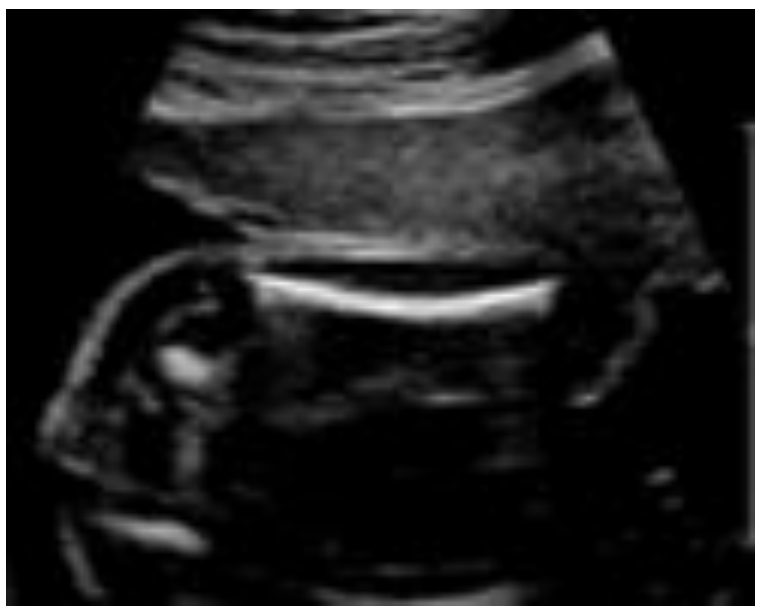

- Angle of insonation between 45 and 90 degrees
- Both ends of the ossified metaphysis visualised
- Magnification at least 30% of the screen

Spine (sagittal)

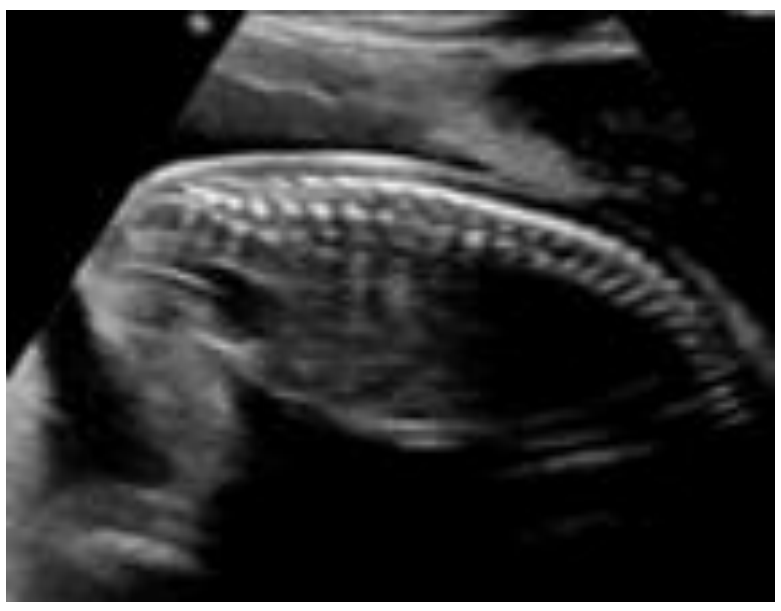

- Mid-sagittal view from neck to sacrum
- Alignment of cervical, thoracic, lumbar and sacral vertebrae visualised for assessment
- Intact posterior skin edge visible
- Amniotic fluid visible beyond the skin
- Magnification at least 30% of the screen

Spine (coronal)

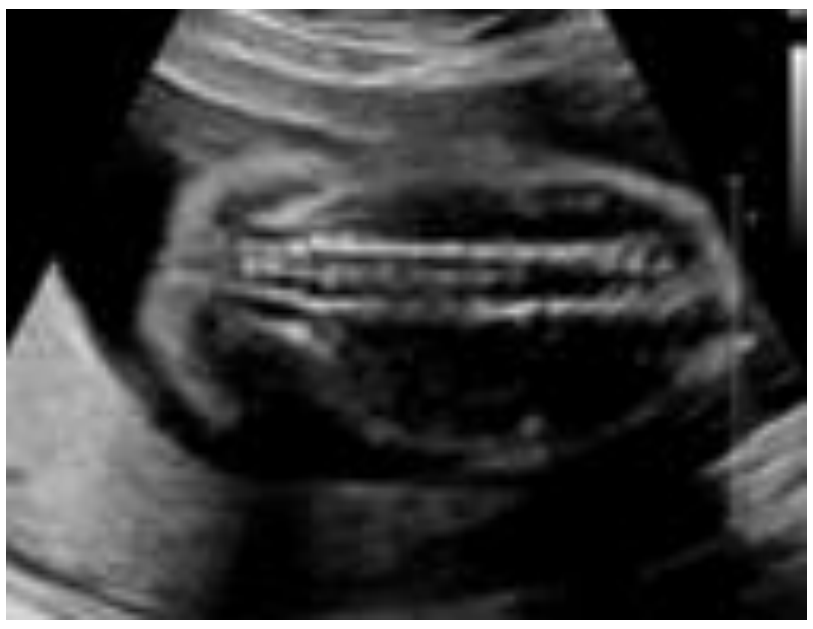

- Coronal view from cervical region to thoracic spine and lumbar region to sacrum (to include iliac bones)
- Alignment of cervical, thoracic, lumbar and sacral vertebrae visualised for assessment
- Magnification at least 30% of the screen

Lips

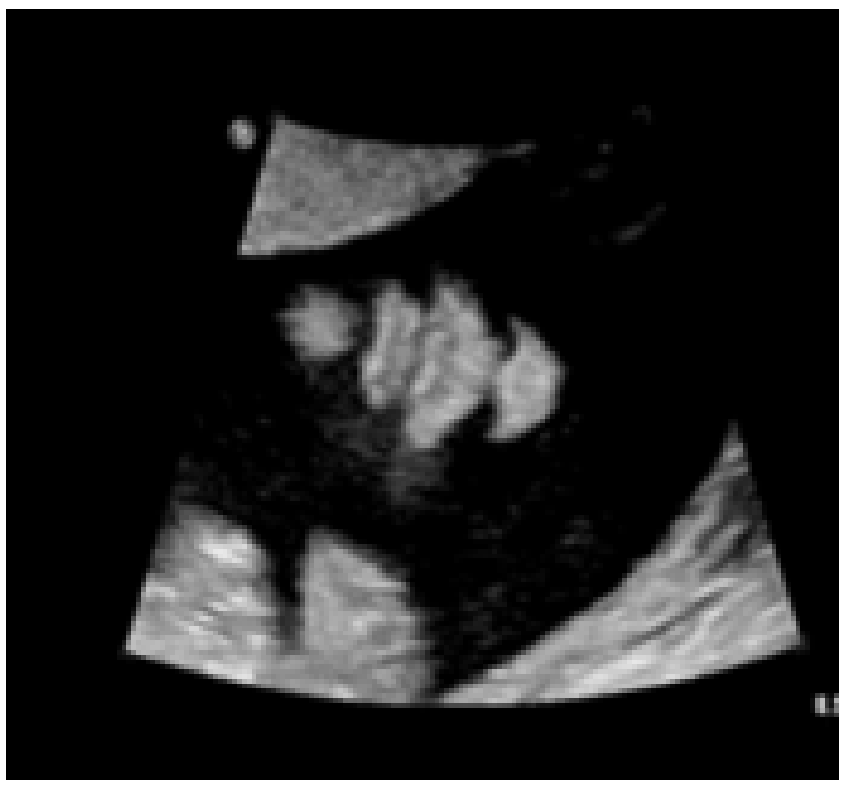

- Coronal view
- Upper lip (and ideally lower lip) visualised
- Two lip angles visualised
- Nasal tip visualised
- Both nostrils visualised
- Magnification at least 30% of the screen

Profile

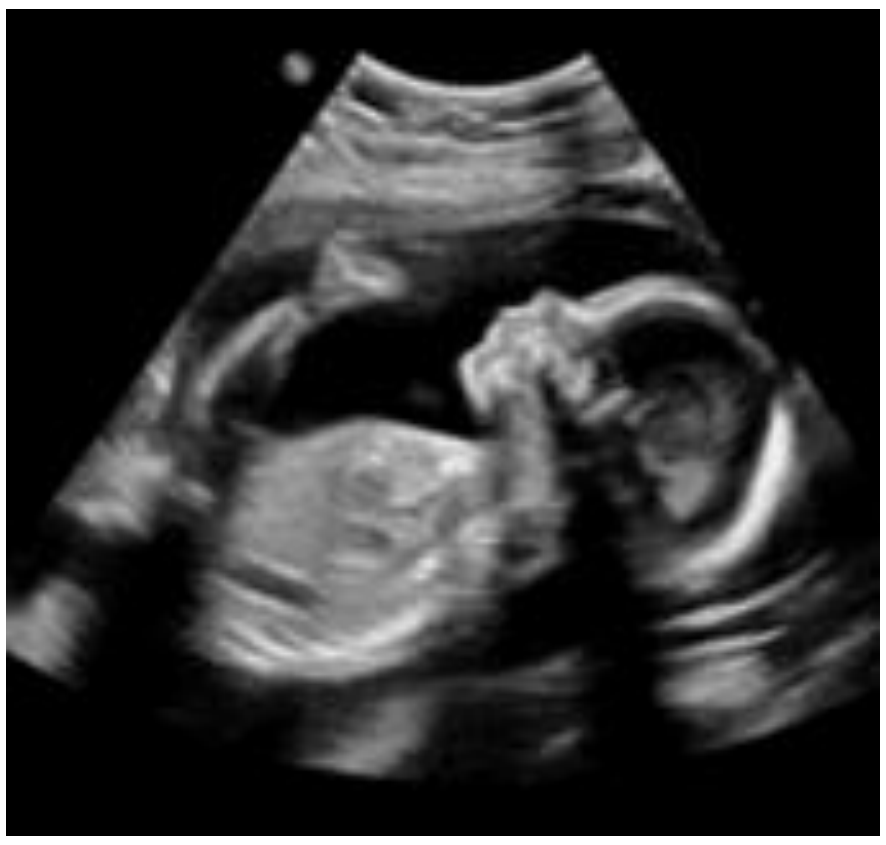

- Mid-sagittal view
- Whole fetal head and proximal 1/3 chest included within image
- Amniotic fluid visible beyond skin/beneath chin for clear delineation of facial structures
- Forehead, nasal bone, nasal tip, lips and chin visualised
- Corpus callosum/cavum septum pellucidum visualised
- Magnification at least 30% of the screen

Kidneys

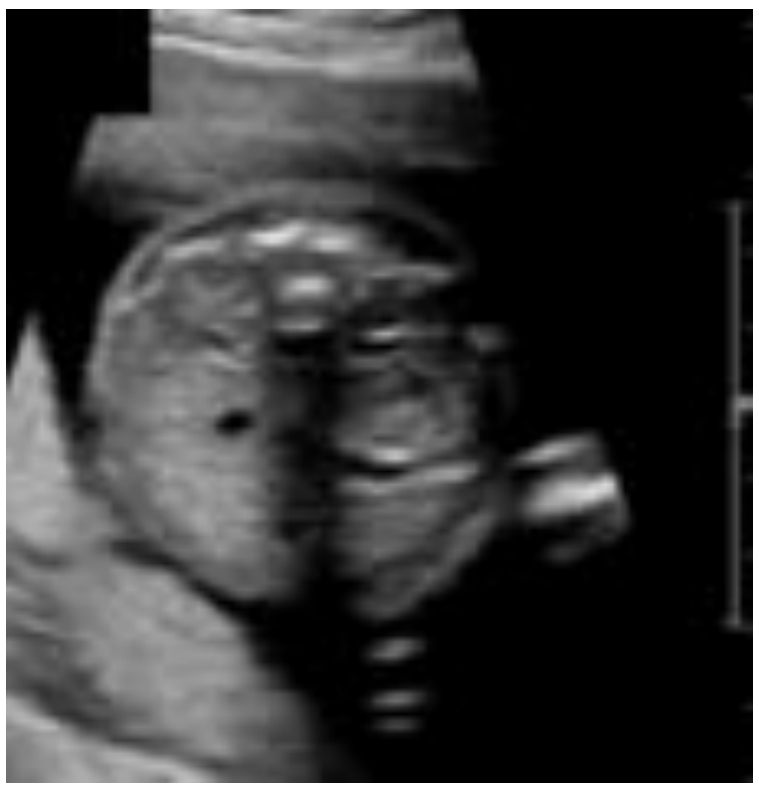

- Transverse view
- Spine at 12 o’clock
- Both kidneys visualised adjacent to spine
- Corticomedullary differentiation or renal pelvis visualised for both kidneys
- Magnification at least 30% of the screen

Heart (4-chamber)

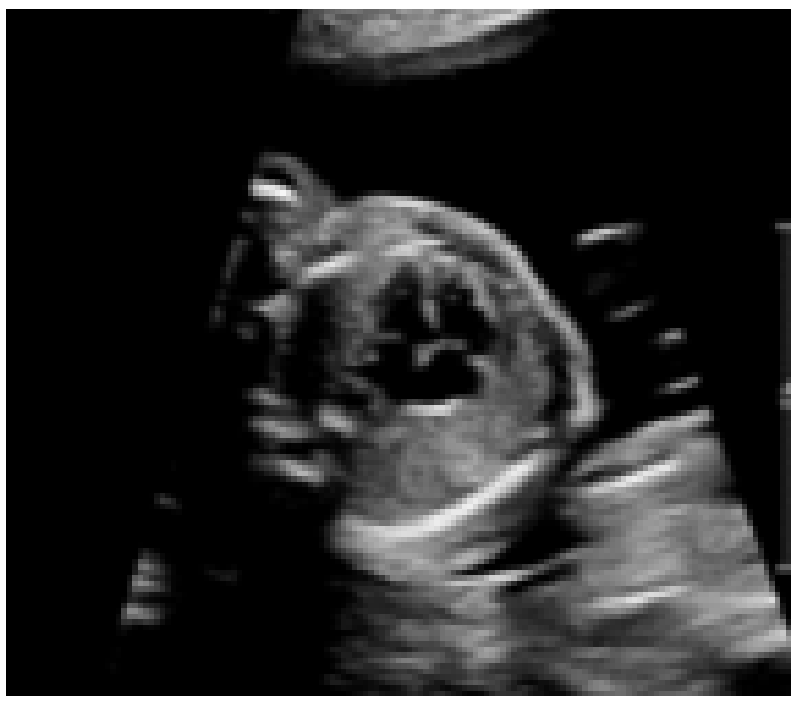

- Transverse view of heart and chest
- Equal sizes right and left atria/ventricles
- Cross-sectional view of descending aorta (and ideally one pair of pulmonary veins) visualised
- Atrio-ventricular valves visualised (open/closed), offset at crux noted
- Intraventricular septum visualised from crux to apex (no drop out artefact)
- Magnification at least 30% of the screen

Heart (left ventricular outflow tract)

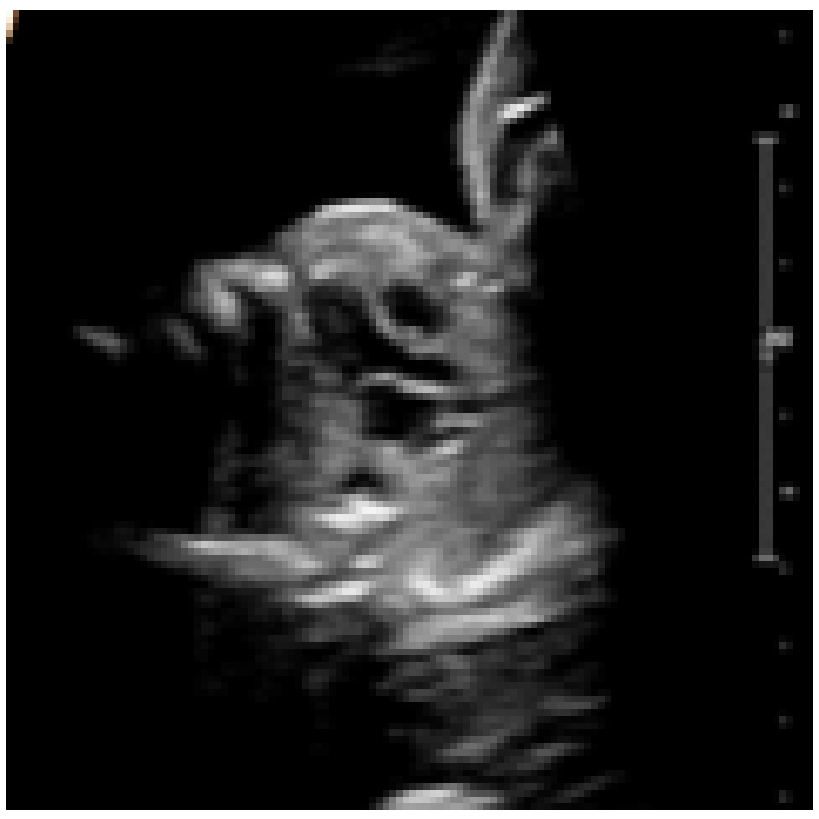

- View of heart and chest
- Aorta continuous with left anterior ventricular wall/IVS showing aortic root/aortic valve (open/closed)
- Left ventricle, right ventricle, left atrium and cross-section of descending aorta visualised
- Magnification at least 30% of the screen

Heart (right ventricular outflow tract or three vessel view)

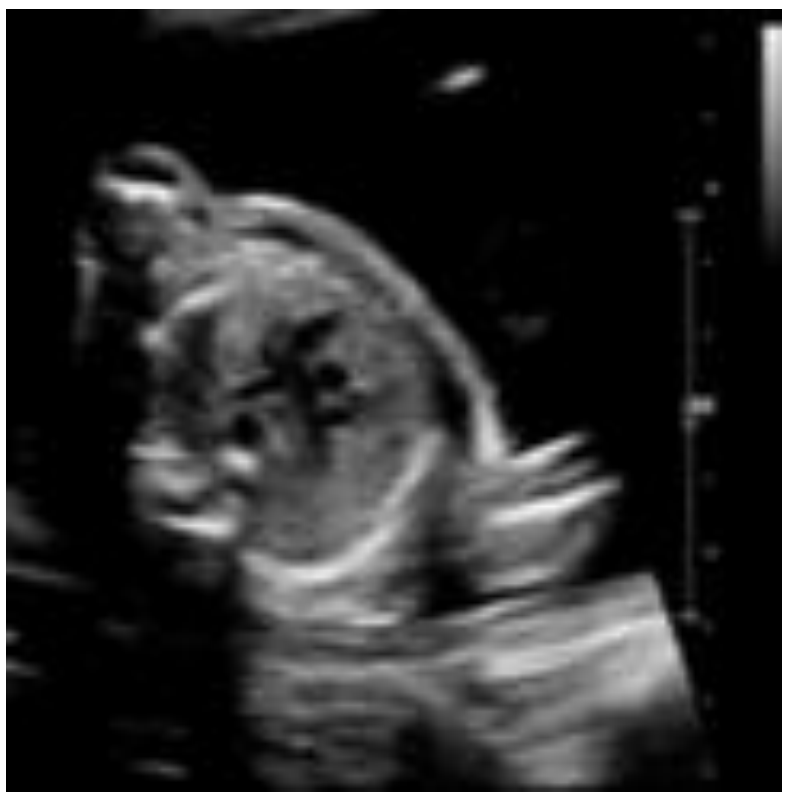

- View of heart and chest
- Pulmonary artery / duct seen in long axis
- Aorta and SVC seen in short axis

Or

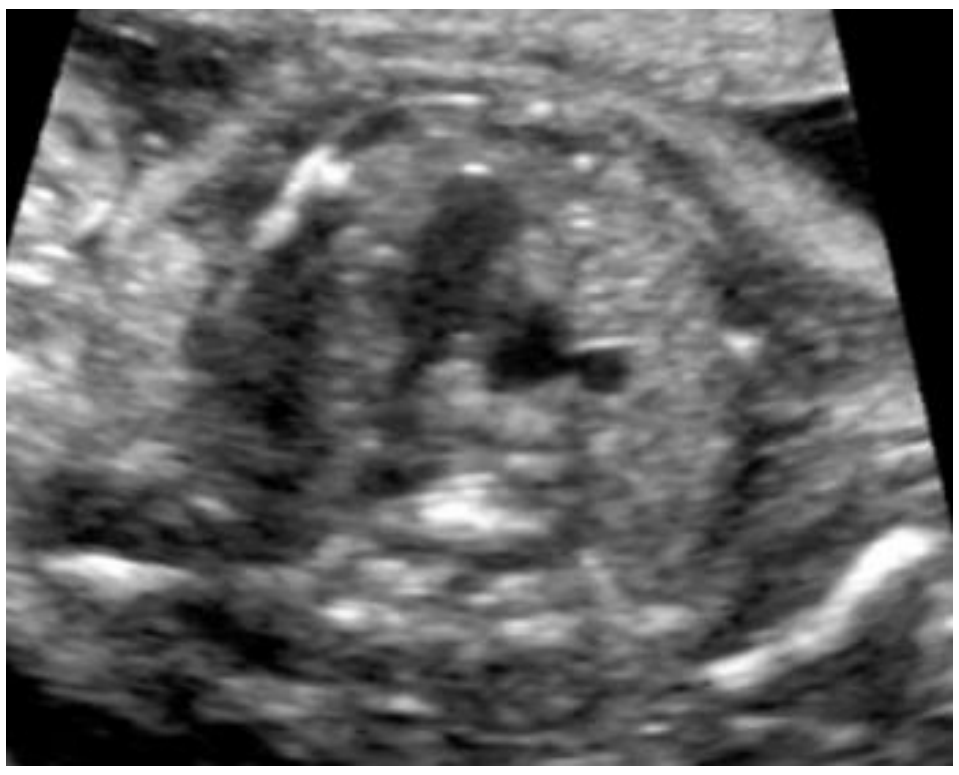

Heart (three vessel tracheal view)

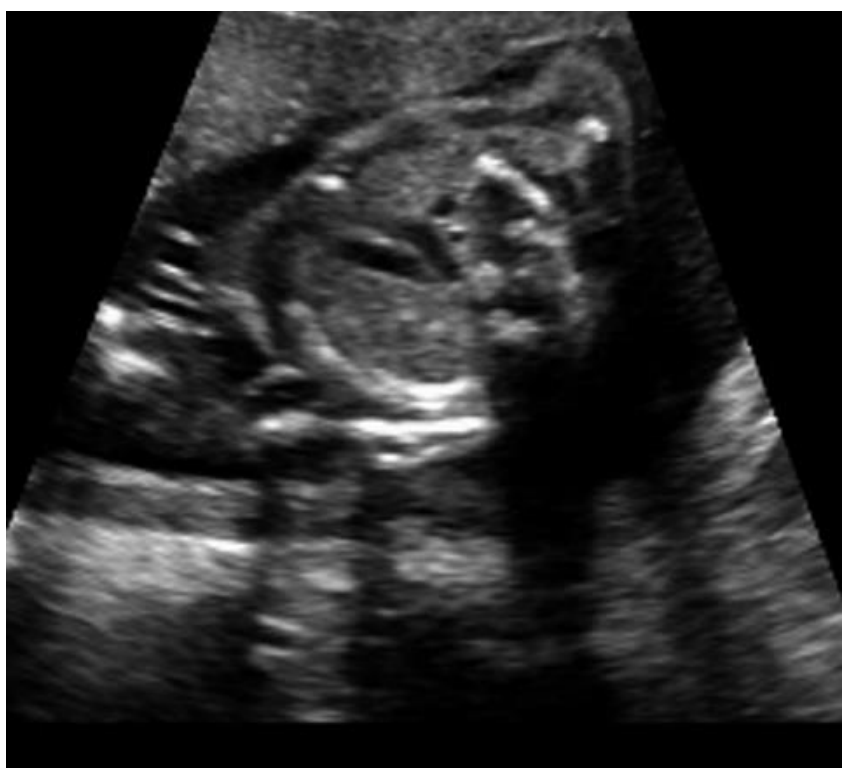

- View of heart and chest
- Pulmonary artery / duct seen in long axis
- Aorta seen in long axis
- SVC seen in short axis

Biometry

You need to measure the following four biometric parameters:

5. Head circumference
6. Biparietal diameter
7. Abdominal circumference
8. Femur length

Please measure each biometric parameter on three separate images, and select the best measurement for use in your report.

Reporting

Next you need to write your report. To do this, you will use a separate computer. Firstly, check that the details are correct on the screen, then click ‘next’.

You will firstly need to complete the biometric parameter that you have measured.

Next scroll down to the ‘Anatomy Details’ section. Here, each anatomical area has multiple choices, you should select the option that best describes what you have seen. You can write extra comments in the ‘comments’ box on the right:

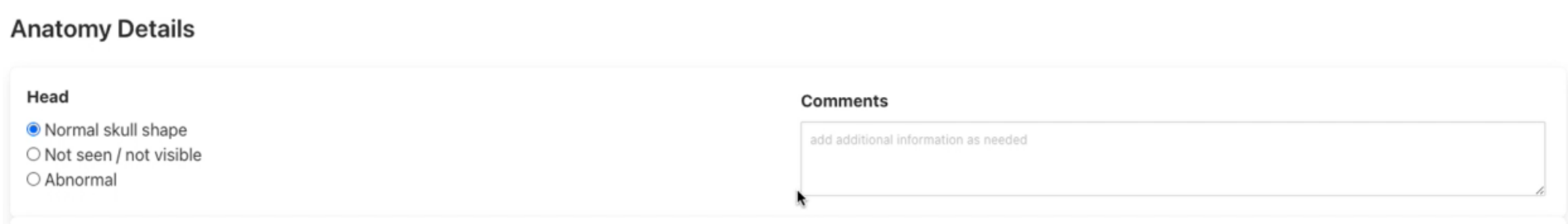

Finally you should select an ‘Overall Outcome’, which has the following three options:

- No follow up
- Repeat scan needed (same department)
- Referral to specialist clinic

Select the option that best describes what you would like to do after the scan.

You should then click ‘Preview’, where you can check you are happy with the report and then click ‘Submit’.

Supplementary information 4: scan protocols

Protocol for AI-assisted anomaly ultrasound scan

- Please perform a modified ultrasound anomaly scan, using the table below as a guide, taking a maximum of 30 minutes.
- Please observe the 6 main anatomical areas:
    - an example image for the 13 planes identified below will be saved automatically if you scan it.
    - the 4 biometric measurements below will be measured automatically and displayed to you.
- Please do not save additional images or measure additional biometric measurements.
- Do not use colour Doppler or spectral Doppler.
- Do not assess the cervix, amniotic fluid volume, placenta, cord vessels, or presentation.

**Area**	**Observe and check**	**Automatically saved image**	**Automatically measured biometry**
Head and neck	Head shape<br>Cavum septum pellucidum<br>Ventricular atrium<br>Cerebellum<br>Nuchal fold	Brain transventricular view<br>Brain cerebellar view	Head circumference<br>Biparietal diameter
Face	Lips<br>Nasal tip	Profile<br>Coronal lips	
Chest	Lungs<br>Heart	Four chamber view<br>Left ventricular outflow tract view (LVOT)<br>Right ventricular outflow tract (RVOT)<br>Three vessel tracheal view (3VT)	
Abdomen	Stomach and position<br>Abdominal wall and cord insertion<br>Diaphragm<br>Kidney<br>Bladder	Abdomen<br>Kidneys transverse view	Abdominal circumference
Spine	Vertebrae<br>Skin covering	Coronal spine<br>Sagittal spine	
Limbs	Femur, tibia and fibular (both legs)<br>Metatarsals (both feet)<br>Radius, ulna, and humerus (both arms)<br>Metacarpals (both hands)	Femur	Femur length

Protocol for manual anomaly ultrasound scan

- Perform a modified ultrasound anomaly scan, using the table below as a guide, taking a maximum of 30 minutes.
- Observe the 6 main anatomical areas:
  - save an example image for the 13 planes identified below.
  - measure the 4 biometric measurements (measure each on three separate images and choose best).
- Do not save additional images or measure additional biometric measurements.
- Do not use colour Doppler or spectral Doppler.

**Area**	**Observe and check**	**Save image**	**Measure biometry**
Head and neck	Head shape<br>Cavum septum pellucidum<br>Ventricular atrium<br>Cerebellum<br>Nuchal fold	1. Brain transventricular view<br>2. Brain cerebellar view	1. Head circumference<br>2. Biparietal diameter
Face	Lips<br>Nasal tip	3. Profile<br>4. Coronal lips	
Chest	Lungs<br>Heart	5. Four chamber view<br>6. Left ventricular outflow tract view (LVOT)<br>7. Right ventricular outflow tract (RVOT)<br>8. Three vessel tracheal view (3VT)	
Abdomen	Stomach and position<br>Abdominal wall and cord insertion<br>Diaphragm<br>Kidney<br>Bladder	9. Abdomen<br>10. Kidneys transverse view	3. Abdominal circumference
Spine	Vertebrae<br>Skin covering	11. Coronal spine<br>12. Sagittal spine	
Limbs	Femur, tibia and fibular (both legs)<br>Metatarsals (both feet)<br>Radius, ulna, and humerus (both arms)<br>Metacarpals (both hands)	13. Femur	4. Femur length

Do not assess the cervix, amniotic fluid volume, placenta, cord vessels, or presentation.

Supplementary information 5: image quality scoring

To assess the saved images in terms of quality, a committee comprising of six experts (consultants in fetal medicine with at least 10 years' experience in obstetrics). These experts had Two metrics were decided upon, a binary score to define if the image was "clinically acceptable" or not, and a continuous quality score based on specified criteria for each plane.

For the clinical acceptability score, we defined "clinically acceptable" as being of *sufficient quality to reasonably assess relevant sonographic signs that correspond to the 11 FASP conditions that are screened for at the anomaly ultrasound scan*. The 11 conditions are defined in the FASP guidelines[4]. To score each image, we asked the experts to consider whether the image was of sufficient quality to allow reasonable assessment of the signs and related conditions shown in Table S2 below. The condition did not necessarily need to be ruled out, as that is not possible for some conditions with a single image plane, but should have allowed reasonable assessment, and give the impression that the sonographer would have been able to exclude the condition during live scanning.

Table S2: FASP conditions relevant to each image plane, and the relevant signs used to consider such diagnoses.

Plane	FASP conditions to assess for (relevant sign(s) in brackets)
**4CH**	- **Major CHD** seen in 4CH view such as hypoplastic left heart or AVSD (abnormal cardiac axis, unbalanced ventricles, septal defects, abnormal offset of AV valves)<br>- **Congenital diaphragmatic hernia** (bowel / liver / stomach in thorax)
**LVOT**	- **Major CHD** seen in LVOT view such as tetralogy of Fallot (lack of continuity of outlet septum with aortic valve)
**RVOT**	- **Major CHD** seen in RVOT view such as tetralogy of Fallot (unbalanced great arteries)
**3VT**	- **Major CHD** seen in 3VT view such as TGA (unbalanced great vessels, abnormal orientation of vessels)
**Abdomen**	- **Gastroschisis** (defect in abdominal wall)<br>- **Exomphalos** (defect in abdominal wall)<br>- **Congenital diaphragmatic hernia** (absent stomach)<br>- **Bilateral renal agenesis** (absent stomach)<br>*Should also allow accurate measurement of abdominal circumference*
**Kidneys**	- **Bilateral renal agenesis** (kidneys not seen)
**Brain TV**	- **Anencephaly** (absence of calvarial bones)<br>- **Spina bifida** (lemon shaped head)<br>- **T13/18** (absent CSP / ventriculomegaly / choroid plexus cysts / strawberry shaped skull / holoprosencephaly / microcephaly)<br>- **Lethal skeletal dysplasia** (clover leaf skull)<br>*Should also allow accurate measurement of head circumference and BPD*
**Brain CB**	- **Spina bifida** (banana shaped cerebellum)<br>- **T13/18** (posterior fossa anomalies / nuchal thickening or cystic hygroma / cerebellar hypoplasia)
**Coronal spine**	- **Spina bifida** (defect in lumbosacral region, loss of normal vertebral alignment)
**Sagittal spine**	- **Spina bifida** (defect in lumbosacral region, possibly involving skin covering)
**Profile**	- **Signs of T13/18** (micrognathia)<br>- **Spina bifida** (frontal bossing)
**Lip**	- **Cleft lip** (defect in upper lip)
**Femur**	- **Lethal skeletal dysplasia** (abnormal shape / reduced echogenicity / absence of extremities)<br>*Should also allow accurate measurement of femur length*

The second metric was an overall quality score, using between three and seven criteria per image plane which were decided upon after discussion, which were felt to be the most important factors in differentiating a good-quality image from a poor-quality image. These criteria are shown in Table S3. One point was given for each criterion, and for each image the score was summed and divided by the total number of criteria for that plane, giving a normalised quality score of between 0 – 1.

Table S3: scoring criteria for each plane to give an overall quality score.

Plane	*Criterion 1*	*Criterion 2*	*Criterion 3*	*Criterion 4*	*Criterion 5*	*Criterion 6*	*Criterion 7*
**Brain TV**	Head symmetrical	Septum cavum pellucidum visible	Posterior ventricular horn visible on at least one side	Magnification such that region of interest fills > 30% of screen by area	Thalami (at level of cerebral peduncles) not visible		
**Brain CB**	Head symmetrical	Septum cavum pellucidum visible	Both cerebellar hemispheres visible and equal in size	Magnification such that region of interest fills > 30% of screen by area	Nuchal fold visible	Cisterna magna visible and measurable	
**Abdomen**	Stomach bubble visible	Abdominal course of umbilical vein or portal sinus visible	Transverse view	Kidneys not visible	Magnification such that region of interest fills > 30% of screen by area	Single length of rib visible	Spine at roughly 3 or 9 o'clock
**Femur**	Both ends of diaphysis clearly visible	Angle of insonation between 45-90 degrees	Magnification such that region of interest fills > 50% of screen by width	Acoustic shadowing seen behind bone			
**Lips**	Upper lip visible	Two nostrils visible	Magnification such that region of interest fills > 30% of screen by area	Chin visible	Oral commissure visible		
**Profile**	Forehead visible	Nasal bone visible	Mid-sagittal plane	Chin visible	Magnification such that	No overlying structures	Whole head and upper

					region of interest fills > 30% of screen by area	(e.g. limb / cord) between probe and face	1/3 chest visible
**Sag. spine**	Vertebral alignment seen from mid thoracic to sacral spine	Clear amniotic / skin boundary seen at lumbo-sacral region	Mid-sag section for lumbo-sacral region at least	Magnification such that region of interest fills > 50% of screen by width			
**Cor. spine**	Coronal lumbosacral spine visible	Both iliac bones visible	Magnification such that region of interest fills > 50% of screen by width				
**Kidney**	Transverse view	Renal cortex visible on both sides	Renal pelvis visible on both sides	Magnification such that region of interest fills > 30% of screen by area	Spine at roughly 12 o'clock		
**4-chamber**	All 4 cardiac chambers visible	Ventricular septum visible from crux to apex (small drop out near AV valves acceptable)	Crux visible (primum atrial septum, AV valves, and ventricular septum)	Magnification such that region of interest fills > 30% of screen by area	One complete rib visible		
**LVOT**	Aorta visible in continuity with ventricular septum	Left ventricle visible	Proximal ascending aorta visible	Magnification such that region of interest fills > 30% of screen by area			
**RVOT/3VV**	Main pulmonary artery / arterial duct visible in long axis	Ascending aorta visible	SVC visible	Magnification such that region of interest fills > 30% of screen by area			
**3VT**	Main pulmonary artery / arterial duct visible in long axis	Transverse aortic arch visible	SVC visible	Magnification such that region of interest fills > 30% of screen by area	Trachea visible	Aorta and arterial duct seen in join in V shape	

Each image was reviewed and scored by two experts using a secure online platform (labelbox.com)[31] using a scoring guideline (outlined below). The experts scoring each image were blinded to the method used to acquire each image, and they were presented in a random order. For the clinically acceptable variable, if the two experts did not agree (i.e. one expert graded the image as clinically acceptable, and the other as clinically unacceptable), then the image was graded a third time by an experienced research sonographer using the same platform, blinded in the same way. The final score was a majority vote between the three. For the overall quality score, the mean of the scores from the two experts was used as the final score.

Results

The proportion of images for each method deemed clinically acceptable are shown in Table S4. Based on the AI models used live in the actual trial, image quality was significantly lower for the AI-acquired images compared to the manual scans. After the retrospective use of new AI models to automatically select the highest quality image, there was a significant improvement in image quality in many planes, meaning that for eight of the 13 planes there was no significant difference between the manually and AI-acquired images. However, for five planes (left ventricular outflow tract, brain transventricular, coronal lips, sagittal spine, and transverse kidneys), the manual scan still resulted in a higher proportion of clinically acceptable images being saved.

*Table S4: image quality as expressed as proportion deemed clinically acceptable. †quality of images selected during actual trial; ‡quality of images selected retrospectively after use of automatic image quality assessment AI model; *p value for McNemar's test comparing manual scans with each of the two AI methods respectively.*

	Proportion of images deemed clinically acceptable (95% confidence intervals)				
**Plane**	**Manual**	**AI-assisted†**	**P value***	**AI-assisted with quality model‡**	**P value***
Abdomen	0.910 (0.815-0.966)	0.746 (0.625-0.845	0.03	0.806 (0.691-0.892)	0.14
Four-chamber	0.674 (0.515-0.809)	0.465 (0.312-0.623)	0.08	0.698 (0.539-0.828)	1.00
Left ventricular outflow tract	0.674 (0.515-0.809)	0.535 (0.377-0.688)	0.21	0.442 (0.291-0.601)	0.03
Right ventricular outflow tract	0.791 (0.640-0.900)	0.512 (0.355-0.667)	0.03	0.674 (0.515-0.809)	0.38
Three-vessel tracheal	0.744 (0.588-0.865)	0.512 (0.355-0.667)	0.06	0.651 (0.491-0.790)	0.45
Brain transventricular	0.925 (0.834-0.975)	0.761 (0.641-0.857)	<0.01	0.791 (0.674-0.881)	0.04
Brain cerebellar	0.806 (0.691-0.892)	0.612 (0.485-0.729)	<0.01	0.806 (0.691-0.892)	1.00
Profile	0.701 (0.557-0.807)	0.642 (0.515-0.755)	0.57	0.716 (0.593-0.820)	1.00
Coronal lips	0.881 (0.778-0.947)	0.333 (0.312-0.560)	0.01	0.537 (0.411-0.660)	<0.01
Sagittal spine	0.791 (0.674-0.881)	0.418 (0.298-0.545)	<0.01	0.388 (0.271-0.515)	<0.01
Coronal spine	0.552 (0.426-0.674)	0.149 (0.074-0.257)	<0.01	0.567 (0.440-0.688)	1
Transverse kidneys	0.806 (0.691-0.892)	0.507 (0.382-0.632)	<0.01	0.567 (0.440-0.688)	<0.01
Femur	0.896 (0.797-0.957)	0.866 (0.760-0.937)	0.77	0.940 (0.854-0.983)	0.51

*Table S5: quality of images as assessed by expert scoring using specific criteria for each plane, normalised to between 0-1. Only images deemed clinically acceptable are included in analysis. †quality of images selected during actual trial; ‡ quality of images selected retrospectively after use of automatic image quality assessment AI model; *p value for Mann-Whitney U test comparing manual scans with each of the two AI methods respectively.*

	Expert-assigned mean quality score (95% confidence intervals)				
**Plane**	**Manual**	**AI-assisted†**	**P value***	**AI-assisted with quality model‡**	**P value***
Abdomen	0.936 (0.910-0.961)	0.894 (0.858-0.931)	0.09	0.921 (0.892-0.949)	0.36
Four-chamber	0.859 (0.820-0.897)	0.865 (0.791-0.940)	0.37	0.903 (0.862-0.944)	0.06
Left ventricular outflow tract	0.784 (0.728-0.841)	0.788 (0.718-0.858)	0.92	0.882 (0.793-0.970)	0.01
Right ventricular outflow tract	0.938 (0.907-0.968)	0.949 (0.908-0.990)	0.5	0.961 (0.921-1.000)	0.06
Three-vessel tracheal	0.831 (0.785-0.877)	0.784 (0.686-0.993)	0.74	0.940 (0.903-0.978)	<0.01
Brain transventricular	0.963 (0.947-0.978)	0.951 (0.930-0.972)	0.47	0.964 (0.945-0.983)	0.75
Brain cerebellar	0.912 (0.879-0.946)	0.910 (0.882-0.940)	0.47	0.901 (0.867-0.936)	0.70
Profile	0.871 (0.834-0.907)	0.817 (0.775-0.860)	0.05	0.680 (0.609-0.751)	<0.01
Coronal lips	0.822 (0.791-0.853)	0.824 (0.762-0.887)	0.71	0.800 (0.734-0.866)	0.87
Sagittal spine	0.873 (0.837-0.909)	0.862 (0.819-0.904)	0.47	0.813 (0.739-0.886)	0.21
Coronal spine	0.639 (0.597-0.680)	0.638 (0.522-0.753)	0.95	0.546 (0.493-0.599)	0.02
Transverse kidneys	0.889 (0.862-0.916)	0.865 (0.826-0.903)	0.29	0.887 (0.823-0.951)	0.02
Femur	0.823 (0.785-0.861)	0.807 (0.765-0.848)	0.53	0.848 (0.804-0.892)	0.12

The results of the expert quality scores for each plane are shown in Table S5. Using the AI models that automatically selected the best quality images, for two planes (left ventricular outflow tract and three vessel tracheal) the experts graded the AI images as superior to the manual images, and for three planes (profile, coronal spine and transverse kidneys) the manual images as superior. The remaining planes were not different between methods.

Supplementary information 6: data loss

Due to software or hardware failures during the study procedures, some data were lost and therefore not available for analysis. For the paired analysis comparing the two methods, only data where a paired datapoint from each method were available were used, unpaired data were discarded. In total there were two pregnant participants who underwent only AI-assisted scans as the sonographer assigned to the manual scan was available, these have been removed from further analyses. The remaining 78 underwent scanning with both methods, and were included in the main outcome measure of diagnostic performance, and also scan duration and sonographer cognitive load. There were 11 pregnant participants in whom either the manual and/or AI-assisted reporting stage (including reporting and image saving) were affected by technical failures. The remaining 67 were included in further analyses. If a technical failure meant that a contemporaneous written report was not possible, a verbal report was recorded by the study team about any suspected malformations and the planned outcome so that the primary outcome measure could still be recorded for these scans. A data loss flow diagram is shown in Figure S1.

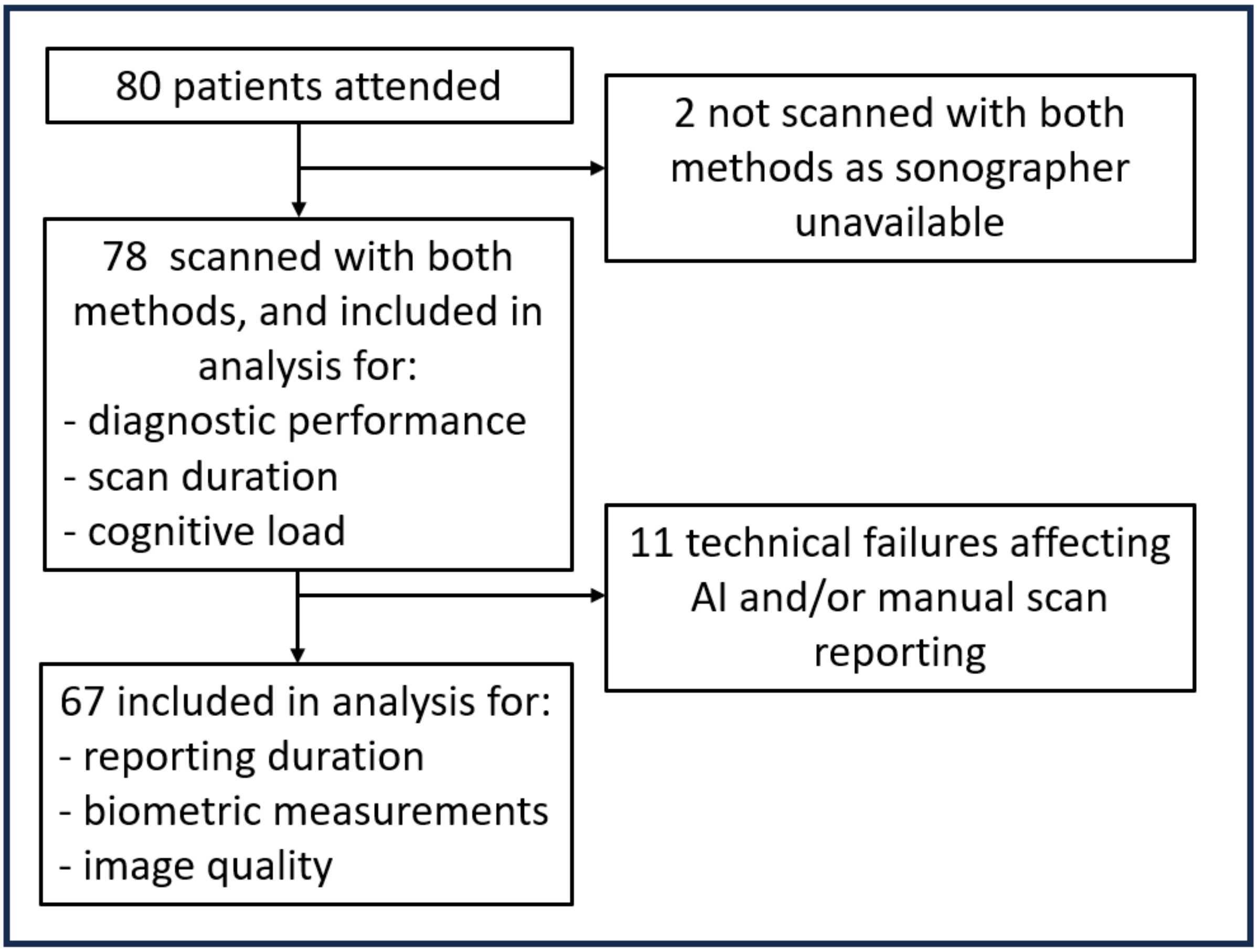

Figure S1: flowchart demonstrating data loss and inclusion in various analyses.
