# Supplementary material for: Artificial Intelligence to Assist in the Screening Fetal Anomaly Ultrasound Scan (PROMETHEUS): A Randomised Controlled Trial
Source: NEJM AI. Author manuscript; Available in PMC 2026 Nov 4. (PMC7619697; doi:10.1056/AIoa2400747)
Supplement: Supplementary file 2 [file EMS218126-supplement-appendix_2.pdf]

Supplementary Appendix

This appendix has been provided by the authors to give readers additional information about their work.

Thomas G. Day, Jacqueline Matthew, Samuel F. Budd, et al. AI to Assist in the Fetal Anomaly Ultrasound Scan: A Randomized Controlled Trial. NEJM Ai. DOI: 10.1056/AIoa2400747.

CONSORT-AI

CONSORT-AI checklist of information to include when reporting a randomised trials of AI interventions

Section	Item	CONSORT 2010 Item[a]	CONSORT-AI Item		Addressed on Page No[b]
Title and Abstract					
**Title and Abstract**	1a	Identification as a randomised trial in the title	CONSORT-AI 1a,b Elaboration	(i) Indicate that the intervention involves artificial intelligence/machine learning in the title and/or abstract and specify the type of model.	1
	1b	Structured summary of trial design, methods, results, and conclusions (for specific guidance see CONSORT for abstracts)		(ii) State the intended use of the AI intervention within the trial in the title and/or abstract.	1
Introduction					
**Background and objectives**	2a	Scientific background and explanation of rationale	CONSORT-AI 2a (i) Extension	Explain the intended use of the AI intervention in the context of the clinical pathway, including its purpose and its intended users (e.g. healthcare professionals, patients, public).	6,7
	2b	Specific objectives or hypotheses			7
Methods					
**Trial design**	3a	Description of trial design (such as parallel, factorial) including allocation ratio			8
	3b	Important changes to methods after trial commencement (such as eligibility criteria), with reasons			n/a
**Participants**	4a	Eligibility criteria for participants	CONSORT-AI 4a (i) Elaboration	State the inclusion and exclusion criteria at the level of participants.	8
			CONSORT-AI 4a (ii) Extension	State the inclusion and exclusion criteria at the level of the input data.	8
	4b	Settings and locations where the data were collected	CONSORT-AI 4b Extension	Describe how the AI intervention was integrated into the trial setting, including any onsite or offsite requirements.	12
**Interventions**	5	The interventions for each group with sufficient details to allow replication, including how and when they were actually administered	CONSORT-AI 5 (i) Extension	State which version of the AI algorithm was used.	Supp. 2
			CONSORT-AI 5 (ii) Extension	Describe how the input data were acquired and selected for the AI intervention.	12, Supp. 2
			CONSORT-AI 5 (iii) Extension	Describe how poor quality or unavailable input data were assessed and handled.	n/a
			CONSORT-AI 5 (iv) Extension.	Specify whether there was human-AI interaction in the handling of the input data, and what level of expertise was required of users.	12

Cite as:

			CONSORT-AI 5 (v) Extension	Specify the output of the AI intervention	13, supp. 2
			CONSORT-AI 5 (vi) Extension	Explain how the AI intervention's outputs contributed to decision-making or other elements of clinical practice.	13, supp. 2
**Outcomes**	6a	Completely defined pre-specified primary and secondary outcome measures, including how and when they were assessed			16
	6b	Any changes to trial outcomes after the trial commenced, with reasons			n/a
**Sample size**	7a	How sample size was determined			16
	7b	When applicable, explanation of any interim analyses and stopping guidelines			n/a
Randomisation					
**Sequence generation**	8a	Method used to generate the random allocation sequence			9
	8b	Type of randomisation; details of any restriction (such as blocking and block size)			9
**Allocation concealment mechanism**	9	Mechanism used to implement the random allocation sequence (such as sequentially numbered containers), describing any steps taken to conceal the sequence until interventions were assigned			9
**Implementation**	10	Who generated the random allocation sequence, who enrolled participants, and who assigned participants to interventions			9
**Blinding**	11a	If done, who was blinded after assignment to interventions (for example, participants, care providers, those assessing outcomes) and how			9
	11b	If relevant, description of the similarity of interventions			n/a
**Statistical methods**	12a	Statistical methods used to compare groups for primary and secondary outcomes			16
	12b	Methods for additional analyses, such as subgroup analyses and adjusted analyses			16
Results					
**Participant flow** (a diagram is strongly recommended)	13a	For each group, the numbers of participants who were randomly assigned, received intended treatment, and were analysed for the primary outcome			18, 19
	13b	For each group, losses and exclusions after randomisation, together with reasons			18, 19, supp. 6

Cite as:

**Recruitment**	14a	Dates defining the periods of recruitment and follow-up			18
	14b	Why the trial ended or was stopped			n/a
**Baseline data**	15	A table showing baseline demographic and clinical characteristics for each group			20
**Numbers analysed**	16	For each group, number of participants (denominator) included in each analysis and whether the analysis was by original assigned groups			21
**Outcomes and estimation**	17a	For each primary and secondary outcome, results for each group, and the estimated effect size and its precision (such as 95% confidence interval)			21-29
	17b	For binary outcomes, presentation of both absolute and relative effect sizes is recommended			n/a
**Ancillary analyses**	18	Results of any other analyses performed, including subgroup analyses and adjusted analyses, distinguishing pre-specified from exploratory			21-29
**Harms**	19	All important harms or unintended effects in each group (for specific guidance see CONSORT for harms)	CONSORT-AI 19 Extension	Describe results of any analysis of performance errors and how errors were identified, where applicable. If no such analysis was planned or done, explain why not.	Biometry: 25<br>Images: 28, 29
Discussion					
**Limitations**	20	Trial limitations, addressing sources of potential bias, imprecision, and, if relevant, multiplicity of analyses			32-33
**Generalisability**	21	Generalisability (external validity, applicability) of the trial findings			33
**Interpretation**	22	Interpretation consistent with results, balancing benefits and harms, and considering other relevant evidence			31-34
Other Information					
**Registration**	23	Registration number and name of trial registry			9
**Protocol**	24	Where the full trial protocol can be accessed, if available			78
**Funding**	25	Sources of funding and other support (such as supply of drugs), role of funders	CONSORT-AI 25 Extension.	State whether and how the AI intervention and/or its code can be accessed, including any restrictions to access or re-use.	78

[a] We strongly recommend reading this statement in conjunction with the CONSORT 2010 Explanation and Elaboration for important clarifications on all the items.
[b] Indicates page numbers to be completed by authors during protocol development.

Cite as:
