# Supplementary material for: Artificial Intelligence to Assist in the Screening Fetal Anomaly Ultrasound Scan (PROMETHEUS): A Randomised Controlled Trial
Source: NEJM AI. Author manuscript; Available in PMC 2026 Nov 4. (PMC7619697; doi:10.1056/AIoa2400747)
Supplement: Supplementary file 3 [file EMS218126-supplement-data_sharing.pdf]

Data Sharing Statement

Thomas G. Day, Jacqueline Matthew, Samuel F. Budd et al. AI to Assist in the Fetal Anomaly Ultrasound Scan: A Randomized Controlled Trial. NEJM AI. DOI: 10.1056/AIoa2400747.

Question	Authors' Response
Will the data collected for your study be made available to others?	No
Would you like to offer context for your decision?	Patient-level imaging data from the trial, and the imaging data used to train the AI models are not available for sharing due to ethical restrictions. The study protocol, patient information sheet, and example consent forms are available on request. The code used to train the AI models is available on request, but the model weights used in the trial are not available.
Which data?	—
Additional information about data	—
How or where can the data be obtained?	—
When will data availability begin?	—
When will data availability end?	—
Will any supporting documents be available?	—
Which supporting documents?	—
Additional information about supporting documents	—
How or where can supporting documents be obtained?	—
When will supporting documents availability begin?	—
When will supporting documents availability end?	—
To whom will data be available?	—
For what type of analysis or purpose?	—
By what mechanism?	—
Any other restrictions?	—
Additional information	—

This statement was posted on March 27, 2025, at ai.nejm.org.
