# Supplementary material for: Artificial Intelligence to Assist in the Screening Fetal Anomaly Ultrasound Scan (PROMETHEUS): A Randomised Controlled Trial
Source: NEJM AI. Author manuscript; Available in PMC 2026 Nov 4. (PMC7619697; doi:10.1056/AIoa2400747)
Supplement: Supplementary file 4 [file EMS218126-supplement-disclosures.pdf]

Sep 25, 2024 07:35:57 EDT
NEJM AI

Ranjit Akolekar

Discloser Identifier: 1199855

Disclosure Purpose: AI-24-00747.R1

Summary of Interests

I do not have any interests to disclose at this time.

Certification

I certify that the information provided in this disclosure is complete and accurate.

Convey Global Disclosure System | AAMC

Anastasija Arechvo

Discloser Identifier: 1199849

Disclosure Purpose: AI-24-00747.R1

Summary of Interests

I do not have any interests to disclose at this time.

Certification

I certify that the information provided in this disclosure is complete and accurate.

Convey Global Disclosure System
AAMC

Sep 07, 2024 01:55:26 EDT
NEJM AI

Matthew Brown

Discloser Identifier: 1199844

Disclosure Purpose: AI-24-00747.R1

Summary of Interests

I do not have any interests to disclose at this time.

Certification

I certify that the information provided in this disclosure is complete and accurate.

Convey Global Disclosure System | AAMC

Sep 09, 2024 06:08:24 EDT
NEJM AI

Samuel Budd

Discloser Identifier: 1199835

Disclosure Purpose: AI-24-00747.R1

Summary of Interests

Company or Organization

Entity	Type	Interest Held By
Fraiya Ltd	Other Business Ownership	Self

Form of Business Description: SB is a co-founder and shareholder of Fraiya Ltd, a University-NHS spinout company that is aiming to commercialise an AI tool for use in the screening obstetric ultrasound scan.
Additional Information:

Certification

I certify that the information provided in this disclosure is complete and accurate.

Convey Global Disclosure System | AAMC

Davide Casagrandi

Discloser Identifier: 1199847

Disclosure Purpose: AI-24-00747.R1

Summary of Interests

I do not have any interests to disclose at this time.

Certification

I certify that the information provided in this disclosure is complete and accurate.

Convey Global Disclosure System
AAMC

Sep 07, 2024 12:40:12 EDT
NEJM AI

Thomas Day

Discloser Identifier: 1193518

Disclosure Purpose: AI-24-00747.R1

Summary of Interests

Company or Organization

Entity	Type	Interest Held By
Fraiya Ltd	Stock	Self

Additional Information: I am a co-founder and shareholders of Fraiya Ltd, a University-NHS spinout company that is aiming to commercialise an AI tool for use in the screening obstetric ultrasound scan

Certification

I certify that the information provided in this disclosure is complete and accurate.

Convey Global Disclosure System | AAMC

Alfonso Farruggia

Discloser Identifier: 1199836

Disclosure Purpose: AI-24-00747.R1

Summary of Interests

Company or Organization

Entity	Type	Interest Held By
Fraiya LTD	Other Business Ownership	Self
**Form of Business Description:** Co-founder and shareholder of Fraiya LTD, a University-NHS spinout company that is aiming to commercialise an AI tool for use in the screening obstrectic ultrasound scan. **Additional Information:**		

Certification

I certify that the information provided in this disclosure is complete and accurate.

Convey Global Disclosure System AAMC

Gavin Guy

Discloser Identifier: 1199845

Disclosure Purpose: AI-24-00747.R1

Summary of Interests

I do not have any interests to disclose at this time.

Certification

I certify that the information provided in this disclosure is complete and accurate.

Convey Global Disclosure System | AAMC

Sep 23, 2024 04:04:04 EDT
NEJM AI

Joseph Hajnal

Discloser Identifier: 1199865

Disclosure Purpose: AI-24-00747.R1

Summary of Interests

Company or Organization

Entity	Type	Interest Held By
FRAIYA Ltd	Stock	Self
**Additional Information:**		

Certification

I certify that the information provided in this disclosure is complete and accurate.

Convey Global Disclosure System | AAMC

Sep 16, 2024 06:46:30 EDT
NEJM AI

Babak Jamshidi

Discloser Identifier: 1199839

Disclosure Purpose: AI-24-00747.R1

Summary of Interests

I do not have any interests to disclose at this time.

Certification

I certify that the information provided in this disclosure is complete and accurate.

Convey Global Disclosure System | AAMC

Bernhard Kainz

Discloser Identifier: 1199861

Disclosure Purpose: AI-24-00747.R1

Summary of Interests

Company or Organization

Entity	Type	Interest Held By
Fraiya Ltd.	Stock Option	Self
**Additional Information:** BK is co-founders and shareholders of Fraiya Ltd, a University-NHS spinout company that is aiming to commercialise an AI tool for use in the screening obstetric ultrasound scan.		

Certification

I certify that the information provided in this disclosure is complete and accurate.

Convey Global Disclosure System | AAMC

Sep 18, 2024 05:27:24 EDT
NEJM AI

Jonathan Lai

Discloser Identifier: 1199842

Disclosure Purpose: AI-24-00747.R1

Summary of Interests

I do not have any interests to disclose at this time.

Certification

I certify that the information provided in this disclosure is complete and accurate.

Convey Global Disclosure System | AAMC

Hua Zen Ling

Discloser Identifier: 1199841

Disclosure Purpose: AI-24-00747.R1

Summary of Interests

I do not have any interests to disclose at this time.

Certification

I certify that the information provided in this disclosure is complete and accurate.

Convey Global Disclosure System | AAMC

Sep 17, 2024 05:26:03 EDT
NEJM AI

David Lloyd

Discloser Identifier: 1199851

Disclosure Purpose: AI-24-00747.R1

Summary of Interests

I do not have any interests to disclose at this time.

Certification

I certify that the information provided in this disclosure is complete and accurate.

Convey Global Disclosure System | AAMC

Sep 23, 2024 10:09:34 EDT
NEJM AI

Jacqueline Matthew

Discloser Identifier: 1199834

Disclosure Purpose: AI-24-00747.R1

Summary of Interests

Company or Organization

Entity	Type	Interest Held By
Fraiya LTD	Stock	Self

Additional Information: I am a co-founder and shareholders of Fraiya Ltd, a University-NHS spinout company that is aiming to commercialise an AI tool for use in the screening obstetric ultrasound scan

Certification

I certify that the information provided in this disclosure is complete and accurate.

Convey Global Disclosure System | AAMC

Owen Miller

Discloser Identifier: 1199854

Disclosure Purpose: AI-24-00747.R1

Summary of Interests

I do not have any interests to disclose at this time.

Certification

I certify that the information provided in this disclosure is complete and accurate.

Convey Global Disclosure System | AAMC

Surabhi Nanda

Discloser Identifier: 1199860

Disclosure Purpose: AI-24-00747.R1

Summary of Interests

I do not have any interests to disclose at this time.

Certification

I certify that the information provided in this disclosure is complete and accurate.

Convey Global Disclosure System | AAMC

Sep 23, 2024 06:00:08 EDT
NEJM AI

Kypros Nicolaides

Discloser Identifier: 1133802

Disclosure Purpose: AI-24-00747.R1

Summary of Interests

I do not have any interests to disclose at this time.

Certification

I certify that the information provided in this disclosure is complete and accurate.

Convey Global Disclosure System | AAMC

Reza Razavi

Discloser Identifier: 1199871

Disclosure Purpose: AI-24-00747.R1

Summary of Interests

Company or Organization

Entity	Type	Interest Held By
Fraiya Ltd	Fiduciary Officer	Self

Official Title: Chief Executive Officer
Position Description:
Additional Information:

Certification

I certify that the information provided in this disclosure is complete and accurate.

Convey Global Disclosure System | AAMC

Sep 17, 2024 04:18:05 EDT
NEJM AI

John Simpson

Discloser Identifier: 1199864

Disclosure Purpose: AI-24-00747.R1

Summary of Interests

I do not have any interests to disclose at this time.

Certification

I certify that the information provided in this disclosure is complete and accurate.

Argyro Syngelaki

Discloser Identifier: 1199850

Disclosure Purpose: AI-24-00747.R1

Summary of Interests

I do not have any interests to disclose at this time.

Certification

I certify that the information provided in this disclosure is complete and accurate.

Convey Global Disclosure System | AAMC

Min Yi Tan

Discloser Identifier: 1199843

Disclosure Purpose: AI-24-00747.R1

Summary of Interests

I do not have any interests to disclose at this time.

Certification

I certify that the information provided in this disclosure is complete and accurate.

Convey Global Disclosure System AAMC

Meekai To

Discloser Identifier: 1199840

Disclosure Purpose: AI-24-00747.R1

Summary of Interests

I do not have any interests to disclose at this time.

Certification

I certify that the information provided in this disclosure is complete and accurate.

Convey Global Disclosure System | AAMC

Sep 09, 2024 05:58:29 EDT
NEJM AI

Lorenzo Venturini

Discloser Identifier: 1199837

Disclosure Purpose: AI-24-00747.R1

Summary of Interests

Company or Organization

Entity	Type	Interest Held By
Fraiya Ltd	Other Business Ownership	Self

Form of Business Description: I am a co-founder and shareholders of Fraiya Ltd, a University-NHS spinout company that is aiming to commercialise an AI tool for use in the screening obstetric ultrasound scan.
Additional Information:

Certification

I certify that the information provided in this disclosure is complete and accurate.

Convey Global Disclosure System AAMC

Trisha Vigneswaran

Discloser Identifier: 1199853

Disclosure Purpose: AI-24-00747.R1

Summary of Interests

I do not have any interests to disclose at this time.

Certification

I certify that the information provided in this disclosure is complete and accurate.

Convey Global Disclosure System | AAMC

Sep 17, 2024 07:11:54 EDT
NEJM AI

Robert Wright

Discloser Identifier: 1199838

Disclosure Purpose: AI-24-00747.R1

Summary of Interests

Company or Organization

Entity	Type	Interest Held By
Fraiya Ltd	Stock	Self
**Additional Information:** I am a co-founder and shareholders of Fraiya Ltd, a University-NHS spinout company that is aiming to commercialise an AI tool for use in the screening obstetric ultrasound scan		

Certification

I certify that the information provided in this disclosure is complete and accurate.

Convey
Global Disclosure System
AAMC

Sep 14, 2024 03:49:04 EDT
NEJM AI

Vita Zidere

Discloser Identifier: 1199852

Disclosure Purpose: AI-24-00747.R1

Summary of Interests

I do not have any interests to disclose at this time.

Certification

I certify that the information provided in this disclosure is complete and accurate.

Convey Global Disclosure System | AAMC
